# Click-Functionalized Biochar as a Spacer-Engineered Interface for Electrochemical Biosensing

**DOI:** 10.1021/acsomega.6c01575

**Published:** 2026-08-14

**Authors:** Antonio Licheri, Veronica Mattarelli, Lorenzo Bartolucci, Rocco Cancelliere, Claudia Mazzuca, Pietro Mele, Silvia Orlanducci, Laura Micheli, Riccardo Salvio

**Affiliations:** † Department of Chemical Science and Technologies, Tor Vergata University of Rome, Via Della Ricerca Scientifica 1, 00133 Roma, Italy; § Department of Industrial Engineering, Tor Vergata University of Rome, Via del Politecnico 1, 00133 Roma, Italy

## Abstract

Biochar, a carbon-rich material produced through biomass pyrolysis, is gaining significant attention in electrochemical sensor development. This material offers dual advantages: it enhances electrode performance while providing functional groups for binding with recognition elements such as antibodies, enzymes, and aptamers. Despite growing interest, practical applications encounter several challenges. The biochar heterogeneous surface could lead to random immobilization of interaction sites and fouling, both of which limit sensor performance, particularly in complex matrices. In addition, surface modification strategies specifically designed to introduce molecular spacers on biochar for electrochemical biosensing remain comparatively underexplored. This work introduces a multistep chemical modification strategy designed to add reactive functional groups to the biochar surface and, in particular, is focused on the development of a molecular spacer on spent coffee ground-derived biochar (pristine biochar) by introducing a covalently bound molecular moiety through azide–alkyne cycloaddition (clicked biochar). Each reaction intermediate was characterized through in-depth chemical–physical characterization, confirming successful functionalization at each step. Crucially, the molecular spacer extends recognition elements further into solution while limiting large protein adsorption. To validate this approach, the performance of an enzymatic biosensor using alkaline phosphatase (AP) and a model substrate was evaluated. The functionalized biochar exhibits improved antifouling properties and better analytical performance compared to that of its unmodified counterpart. A kinetic study conducted using chronoamperometry (CA) shows an approximately 2.2-fold increase in the apparent maximum catalytic current for clicked biochar compared with pristine biochar, while the apparent Michaelis–Menten constants remained comparable, confirming improved enzyme accessibility and enhanced catalytic efficiency of the covalently immobilized enzyme. Furthermore, electrochemical impedance spectroscopy (EIS) confirmed the antifouling ability of this material, resulting in a higher reduction in nonspecific adsorption compared to pristine biochar, as highlighted by a change in charge transfer resistance (*R*_ct_).

## Graphical abstract

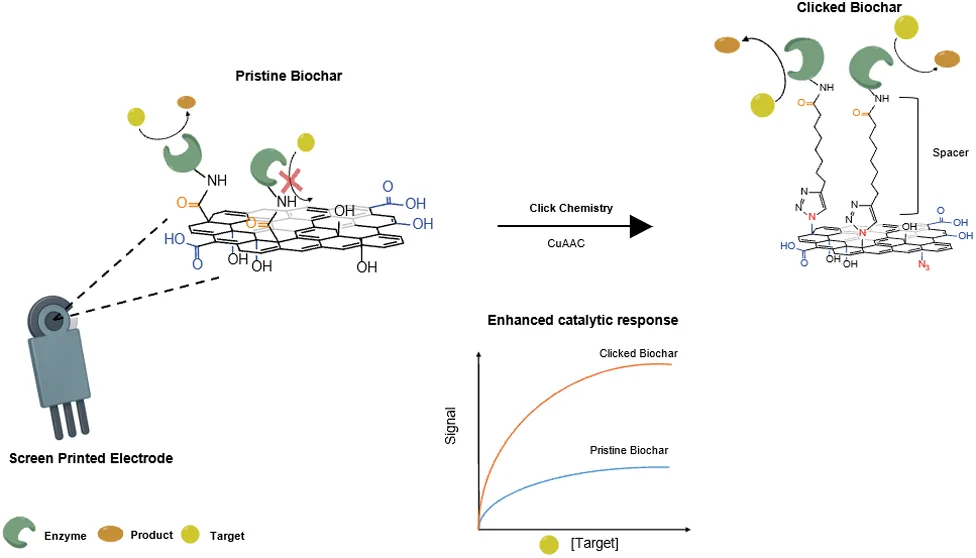

## Introduction

In the last two decades, carbon-based materials^1^ have impressively attracted the attention of the scientific community as they find applications in a variety of fields such as energy storage,^2–5^ biomedicine,^6–8^ catalysis,^9–12^ and many others.^1,13^

Among the various carbon-based materials, biochar,^14–17^ produced through the pyrolysis of biomass feedstock, has recently gained attention in agriculture and environmental remediation.^15,18^ Indeed, its use is not limited to this field as it has been proven that it can be successfully applied in electroanalytics.^15,19^ Biochar possesses a peculiar surface characterized by abundant oxygen-containing functional groups including carboxyl, hydroxyl, carbonyl, and epoxide groups, which arise from the pyrolysis process^17,20,21^ and provide multiple anchor points for chemical modification.^17,21,22^ This material exhibits high surface areas (typically 100–500 m^2^/g),^20,21,23,24^ excellent biocompatibility,^25–27^ tunable porosity,^20,21,23^ and good chemical stability,^21,23,28^ making it particularly attractive for electrochemical applications.^20,21,27^

Electroanalytical techniques are extremely promising due to their rapid response, high sensitivity, and low operational costs. Biochar, in this context, is extremely interesting as a material to modify screen-printed electrodes (SPEs)^29–31^ and improves their properties through enhanced electron transfer kinetics and increased electroactive surface area.^19,29,32^

Other carbon-rich nanomaterials such as graphene and carbon nanotubes have been widely employed in electroanalytical systems, including energy storage, electrocatalysis, and environmental remediation. However, despite their excellent conductivity, their surface functionalization still relies largely on defect engineering, oxidative treatments, or π–π interactions, which generate heterogeneous and nondeterministic distributions of reactive sites. Recent analyses of graphene-based biointerfaces highlight persistent challenges in achieving uniform functional group density and reproducible molecular architectures,^33^ while similar limitations have been reported for CNT-based biosensing platforms, where probe immobilization often depends on randomly distributed oxidation sites.^34^

Biochar and its derivatives offer a fundamentally different and complementary set of advantages. First, biochar originates from abundant, low-cost, and renewable biomass feedstocks, providing an environmentally sustainable alternative to conventional carbon nanomaterials. Second, its physicochemical properties can be precisely tailored by adjusting pyrolysis parameters—such as temperature, residence time, and heating rate—and by selecting specific biomass precursors (e.g., corncob, rice straw, peanut shell). This tunability enables controlled modulation of porosity, surface chemistry, and functional group density, allowing optimization for specific sensing applications.

Moreover, unlike classical spacer-based immobilization strategies that typically yield statistical distributions of probe spacing and orientation, recent work has shown that nanoscale control over the probe density is essential for enhancing hybridization efficiency and signal transduction.^35^ The structural versatility of biochar provides a platform capable of supporting more controlled and application-specific immobilization environments, thereby addressing limitations associated with both carbon nanomaterial-based and conventional spacer-based approaches.^17,20,36^ A comparative summary of the main features of biochar, graphene/CNT nanomaterials, and conventional spacer-based immobilization strategies for biosensing is provided in Table S1.1.

Despite these advantages, several challenges limit the widespread application of all biochars in biosensing platforms because they depend on the biomass origin and pyrolysis temperature.^37^ Higher pyrolysis temperatures improve the electrochemical properties of biochar by creating larger graphitic domains and better electrical conductivity.^28,36,38^ However, higher temperatures simultaneously reduce the abundance of oxygen-containing functional groups, which are crucial for the covalent immobilization of biorecognition elements, such as enzymes and antibodies.^39^ This introduces an inherent trade-off between achieving an optimal electrochemical performance and maintaining sufficient surface functionalization capacity for effective biosensor applications.

Recent electrochemical biosensors that require immobilizing enzymes onto biochar surfaces have been made possible by the utilization of biochar functional groups as active sites to bind biomolecules through cross-linking processes.^21,40–42^ However, when biomolecules such as enzymes or antibodies are directly immobilized onto biochar surfaces, their proximity to the carbon matrix often results in several detrimental effects: (i) random immobilization of biorecognition elements, often leading to nonoptimal orientation and reduced binding efficiency, (ii) steric hindrance of active sites or binding domains due to surface constraints, and (iii) limited accessibility for target analytes due to restricted conformational freedom.^21^ Biochar’s heterogeneous surface leads to nonspecific adsorption of protein organic compounds and fouling (not to be confused with the electrochemical fouling or passivation of the screen-printed electrode) due to hydrophobic and electrostatic interactions. Its high surface area and porous structure make it an effective low-cost sorbent for water and soil remediation, engaging in adsorption, ion exchange, and microbial interactions with various contaminants, including heavy metals and nutrients. Biochar is often modified to enhance its effectiveness against specific contaminants, such as short-chain PFAS and antibiotic-resistant bacteria.^43–45^

This nonspecific adsorption negatively impacts the signal-to-noise ratio and reduces the biosensor sensitivity.

At the present time, only a few limited and recent examples of chemical modification of biochar have been reported,^17,46–49^ and this research area remains largely unexplored. Most biochar biosensors rely on oxidation/activation and generic cross-linkers rather than engineered spacer layers on the biochar itself.^19–21,50^ Additionally, challenges such as reproducibility, electrical conductivity, and long-term stability need to be addressed to facilitate widespread adoption in biosensing technologies. To overcome these issues, strategic chemical functionalization can enable the covalent immobilization of biorecognition elements while addressing the proximity-related limitations described above.

Some of them rely on treatment with common laboratory reagents such as sodium hydroxide,^46^ inorganic acids,^47,51^ and mild oxidizing agents, including hydrogen peroxide,^46^ whereas stronger oxidants, including potassium permanganate^47^ or nitric acid,^46,49^ can induce more extensive structural and surface alterations. In addition, *doping* strategies^19^ have been explored to introduce heteroatoms—most commonly nitrogen—into the carbon framework, typically via annealing of biochar with nitrogen-rich precursors such as urea, thiourea, melamine, or ammonia, thereby broadening the range of materials available for electroanalytical applications. Beyond molecular functionalization, incorporation of metallic nanoparticles (e.g., Au, Pt, or Pd),^21,51^ metal oxide nanoparticles (e.g., TiO_2_ or ZnO),^21^ and metal salts (such as Fe^III^, Al^III^ and Hg)^48^ has also been pursued; in these cases, impregnation of the precursor followed by pyrolysis leads to the embedding of metal salts, oxides, or nanoparticles within the carbon matrix, yielding composite biochars with modified surface chemistry, enhanced conductivity, and substantially altered electrochemical properties that can be exploited in electroanalytical and electrocatalytic applications.

Further chemical functionalization has been achieved through the use of *N*-hydroxysuccinimide (NHS) and 1-ethyl-3-(3-(dimethylamino)­propyl)­carbodiimide (EDC), which activate carboxylic groups present on the biochar surface,^20,40–42^ as well as through the use of dialdehydes capable of forming imine linkages with nitrogen atoms present in the material.^50^ However, these latter procedures are constrained by the relatively low abundance of reactive surface functionalities and, in the case of dialdehydes, by the reversible nature of imine bond formation. The use of nitric acid to activate biochar and related carbon materials prior to their application in sensing and biosensing is documented.^46,49^ Acid oxidation removes inorganic impurities and ash, increases surface area, and introduces oxygen-containing functional groups, e.g., carboxyl and hydroxyl moieties, which provide anchoring sites for biomacromolecules and improve interfacial properties. In this context, the present work introduces a distinct and complementary modification route.^52–55^

In this study, the authors report a novel stepwise procedure for the preparation of multiple biochar derivatives, in which molecular spacers are introduced to physically separate the biorecognition elements from the biochar surface. The proposed strategy employs azide–alkyne cycloaddition, i.e., click chemistry, to covalently attach alkyl chains that function as molecular arms, extending immobilized biomolecules into solution, thereby mitigating the effects of random immobilization, reducing steric hindrance, and improving substrate accessibility while maintaining stable covalent attachment. The derivatives were characterized using a comprehensive array of techniques, including FT-IR and Raman spectroscopy, scanning electron microscopy (SEM) and energy-dispersive X-ray (EDX), thermogravimetric analysis (TGA), cyclic voltammetry (CV), chronoamperometry (CA), and electrochemical impedance spectroscopy (EIS). The experimental results clearly demonstrate the effectiveness of this approach for overcoming the inherent limitations of direct biochar surface modification and indicate the significant potential of these derivatives for electroanalytical applications and chemical sensor fabrication. Finally, this strategy leads to biochar modification at the bulk level prior to electrode deposition, ensuring high reproducibility and reducing batch-to-batch variability.

## Results and Discussion

### Biochar Modification Strategy and Characterization

The nonmodified biochar (called pristine biochar, SCG) produced through biomass pyrolysis is essentially a graphitic or a graphene-like material characterized by a low amount of oxidized carbon atoms and large regions of conjugated sp^2^ carbon atoms.^17,56,57^ Such a structure hardly undergoes effective and extensive functionalization under pristine conditions. In this study, biochar SCG550 (called only SCG550), obtained in previous research from the pyrolysis of spent coffee grounds at 550 °C,^58^ has been selected to be oxidized before the functionalization.^59^ The pyrolysis temperature of the starting material strongly influences its properties and composition. Lower pyrolysis temperatures (350–550 °C) are associated with a higher presence of oxidized functional groups,^60,61^ resulting in potentially greater reactivity, whereas higher pyrolysis temperatures lead to the formation of larger graphitic domains. SCG550, pyrolyzed at 550 °C, was chosen as it may represent a suitable compromise between chemical reactivity and electrochemical properties. The starting material (SCG550) underwent harsh oxidation in two different conditions, i.e., nitric acid and aqueous permanganate, leading to **Ia** and **Ib**, respectively. After each oxidation process, material (**I**) is likely to present a relevant number of hydroxyl units, epoxides and carboxylates, similarly to graphite oxide.^62,63^ A postulated structure for oxidized biochar is given in Scheme 1(**Ia**, **Ib**). Both oxidation procedures were carried out in a water mixture under 1–4 h of sonication. The infrared modified materials revealed significant differences in the oxidation level, attributable to the stronger oxidizing power of the permanganate mixture (Figure 1a–c).

**Figure 1:**
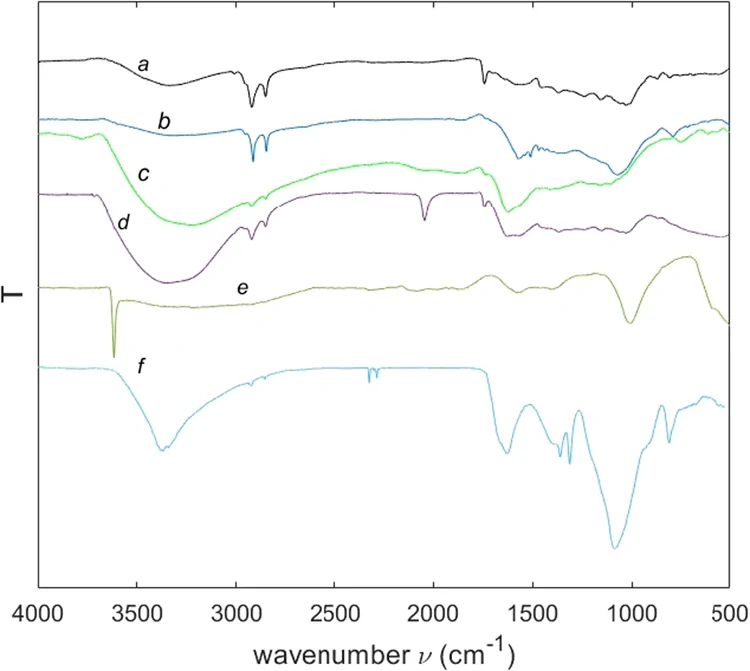
FT-IR spectra of the following materials: biochar SCG550 (a), **Ia** (b), **Ib** (c), **II** (d), **III** (e), and **IV** (clicked biochar) (f).

**Scheme 1:**
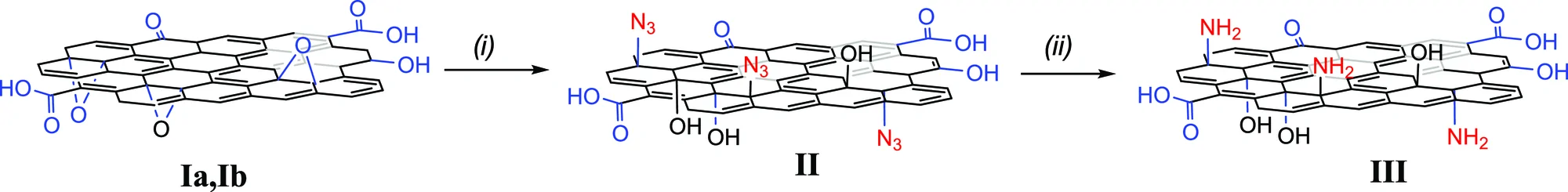
Reaction Steps for the Preparation of the Indicated Materials^a^

a Conditions: (i) sodium azide, AcCN/H_2_O 50 °C, 7 days; (ii) NH_2_NH_2_, H_2_O reflux, and 1 d.

In particular, the FT-IR spectrum of SCG550 shows weak bands primarily linked with carbonyl and carboxylate functionalities, in the 1700–1500 cm^–1^ area, along with a broad band in the 3300–3600 cm^–1^ region attributed to hydrogen-bonded hydroxyl groups; furthermore, bands attributable to C–O stretching and OH deformation of COOH (about 1200–1100 cm^–1^) and CH stretching (at about 2900 cm^–1^) are present. These features are consistent with literature reports on biochar produced at similar temperatures.^64^

Upon oxidation with permanganate, material **Ib** exhibits a marked increase in the intensity and breadth of the 3300–3600 cm^–1^ band (see above) and the appearance of more pronounced absorptions in the 1700–1600 cm^–1^ region, which can be ascribed to both conjugated C=C and C=O stretching modes. These spectral changes are indicative of a higher density of oxygenated functionalities and closely resemble those observed for strongly oxidized biochars and graphite oxide-like materials reported in the literature,^63,65^ confirming that the permanganate treatment induces a deeper oxidation of the carbon framework than nitric acid. Conversely, material **Ia** only shows minor variations with respect to SCG550, supporting the conclusion that the nitric acid protocol yields a more limited functionalization under the conditions adopted.

The scanning electron microscopy (SEM) imaging confirmed a significant change in the chemical nature of material **Ib** by revealing clear morphological changes from the initial one (see Figures S2.1–S2.12 in the Supporting Information). More specifically, SEM micrographs of SCG550 reveal aggregates with relatively smooth particle surfaces and limited microporosity. After permanganate oxidation, material **Ib** displays a markedly rougher surface, with the emergence of microcracks, fissures, and a more open, fragmented texture, along with an apparent increase in surface porosity. These morphological features are consistent with partial disruption of the original carbon domains and with the introduction of a higher density of edge sites and oxygenated groups on the particle surface. Significant changes in surface roughness and porosity have been reported for biochars subjected to different chemical and thermal treatments.^66–68^ Therefore, the alterations observed for **Ib**, as well as its further modified derivatives, directly reflect the underlying chemical modification of the biochar matrix and rationalize its distinct spectroscopic and thermogravimetric behavior compared to SCG550 and **Ia**. Moreover, the thermogravimetric analysis (TGA) plot of **Ib** highlights a markedly different behavior whenever compared with the pristine biochar and **Ia**.

In the second step, **Ia** and **Ib** were separately reacted in the presence of an excess of sodium azide in a 2/1 mixture v/v of acetonitrile/water (Scheme 1). The mixture was sonicated and heated at reflux for 2–7 days. After this treatment, the FT-IR spectrum of the reaction on **Ia** does not show any change compared with the starting material, suggesting that no effective functionalization occurred. On the other hand, material **Ib** afforded **II**, as its FT-IR spectrum significantly changed compared to that of the precursor material **Ib**; an intense stretching at 2100 cm^–1^ appeared with a clear indication of an effective functionalization of the surface with the −N_3_ unit (see Figure 1d). The azide functionalization can be attributed to an epoxide ring opening, as postulated in Scheme 1. Elemental analysis performed by energy-dispersive X-ray spectroscopy (EDX) confirms the presence of azide groups, as evidenced by a significantly higher nitrogen content in **II**, i.e., 10.0 wt %, compared to the pristine biochar used as a starting material (3.7 wt %, see Section S3 in the Supporting Information), which is known to contain only trace amounts of nitrogen according to literature reports.^69,70^

The azide derivative **II** can be further treated either through a proper reduction procedure to afford material **III** (see Scheme 1) or modified through a “click chemistry”^71^ approach (Scheme 2). In the first case, **II** was reacted in the presence of an excess of hydrazine in water at room temperature for 20 h. Evidence of the reduction is apparent in both the IR spectrum (Figure 1e) and the thermogravimetric analysis (TGA, Figure 2). In the latter, the results indicate that the reduced material **III** exhibits remarkable thermal stability under pyrolytic conditions, retaining approximately 85% of its weight up to 1050 °C.

**Figure 2:**
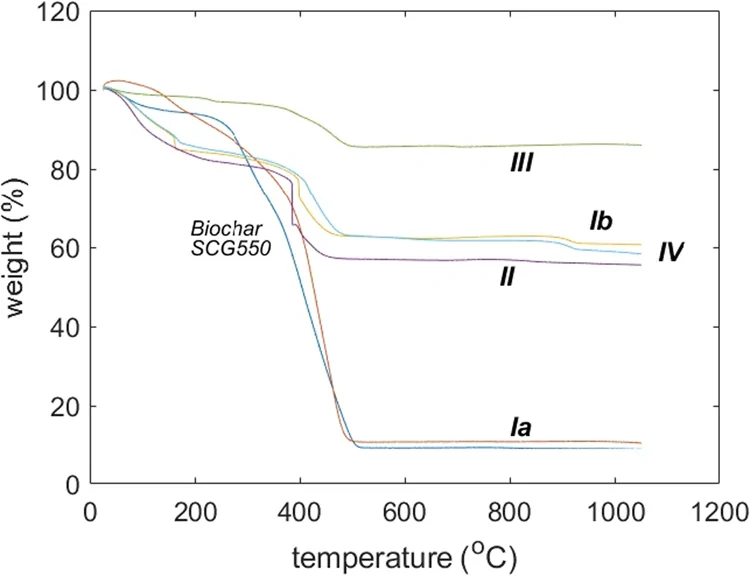
Thermogravimetric analysis of pristine biochar SCG550 in comparison with functionalized materials **Ia**, **Ib**, **II**, **III**, and **IV** (clicked biochar). Measurements carried out under air at a heating rate of 10 °C/min.

**Scheme 2:**
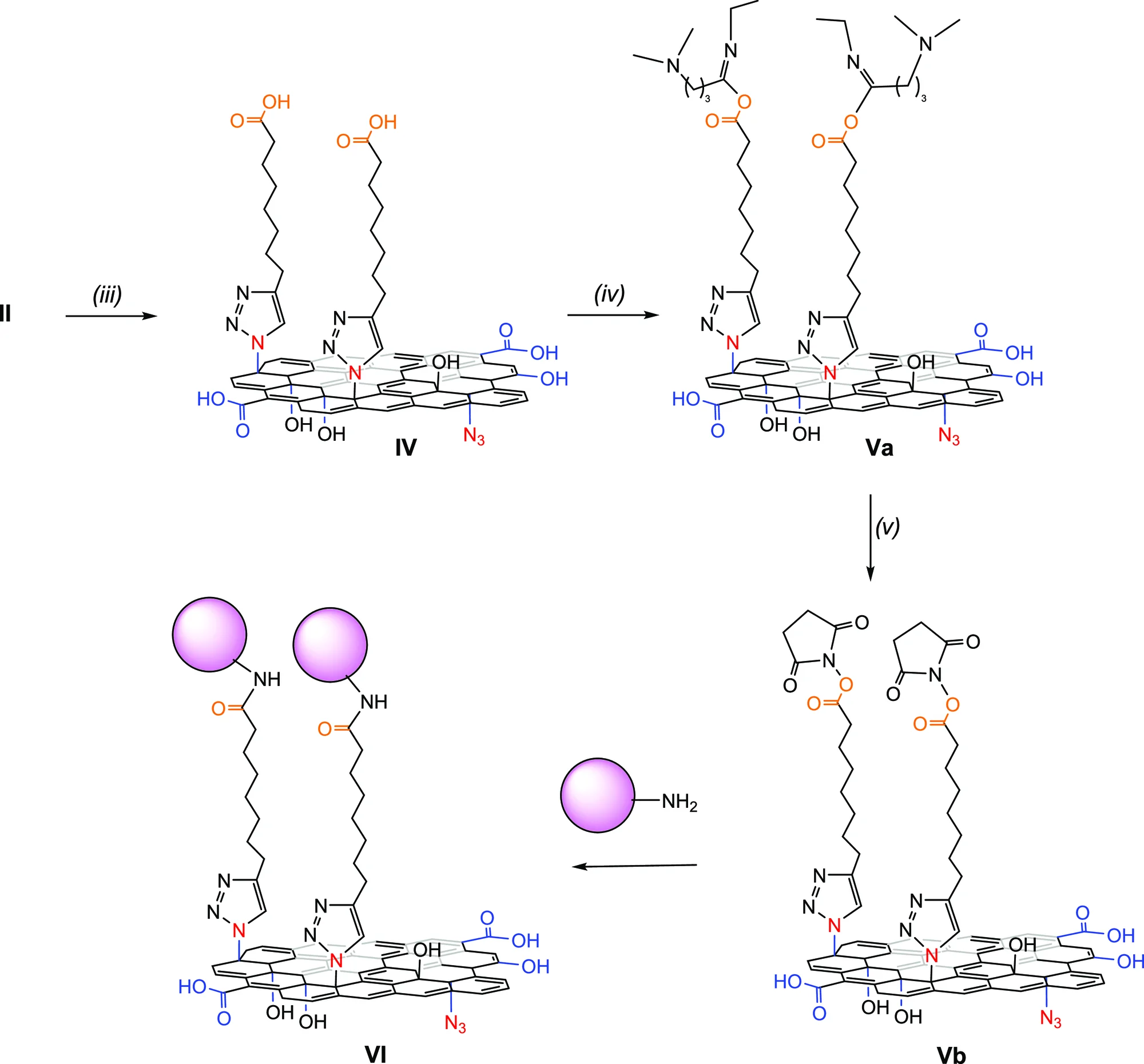
Preparation of Material **IV** (Clicked Biochar) Provided with a C_10_ Alkyl Chain and Strategy for the Immobilization of Biological Macromolecules Affording Material **VI** ^a^

a Conditions: (iii) 10-undecynoic acid, sodium ascorbate, CuSO_4_; (iv) *N*-(3-dimethylaminopropyl)-*N*′-ethylcarbodiimide; and (v) *N*-hydroxysuccinimide. The violet ball represents the alkaline phosphatase (AP) enzyme.

In the last preparative procedure, material **II**, after exhaustive sonication, was dispersed into a 2:1 (v/v) mixture of water and *tert*-butanol. After the addition of 10-undecynoic acid, copper­(II) sulfate, and sodium ascorbate, useful to generate Cu^I^ *in situ*, the mixture was stirred for 14 days at room temperature. The selection of 10-undecynoic acid was based on the need to optimize the compromise between enhanced biorecognition performance and efficient electrochemical communication. Extended molecular spacers can significantly reduce steric hindrance around immobilized biomolecules and improve substrate accessibility but also increase the thickness of the organic layer, which slows down product diffusion and apparent electron transfer efficiency. This fundamental limitation necessitates careful consideration of spacer length to maintain an adequate electrochemical response. The C10 alkyl chain represents a practical compromise that provides sufficient extension to project biorecognition elements away from the biochar surface while preserving reasonable electron transfer rates. This choice is further supported by previous studies demonstrating improved enzymatic activity with decamethylene-based linkers in electrochemical biosensor applications.^72–75^ The FT-IR spectrum indicates the successful completion of the reaction by the disappearance of the azide stretching band and the presence of the aliphatic stretching signals in the 2700–2900 cm^–1^ region (see Figure 1f)

Thermogravimetric analysis (TGA), whose results are reported in Figure 2, further supports the different oxidation levels and subsequent chemical transformations. SCG550 shows an initial minor mass loss below ca. 150 °C, attributed to adsorbed moisture, followed by a broad main decomposition step extending up to 800–900 °C, in line with previous reports for biochars obtained at 500–600 °C. The biochar oxidized with nitric acid (material **Ia**) does not display significant modifications in the TGA analysis. In contrast, material **Ib** exhibits an increased low-temperature weight loss and a more pronounced multistep profile, consistent with the presence of thermally labile oxygen-containing groups such as carboxylates introduced during permanganate oxidation. The conversion of material **Ib** into the azide derivative **II** does not show a very different profile, indicating a similar molecular structure. On the other hand, in reduced material **III**, the onset of the major mass loss is shifted to higher temperatures and the residual mass at 1000–1050 °C becomes significantly larger. This trend suggests that the postoxidation processes preserve enough functional groups for additional derivatization while partially eliminating the most labile oxygenated moieties and promote the formation of a more thermally robust carbon framework. The evolution of the TGA profiles therefore mirrors the chemical pathway and is coherent with the behavior reported for other oxidized and subsequently reduced biochar-based materials.^63,66^

In addition, a micro-Raman analysis was performed on the materials prepared in this study (see Figure 3 and Section S4). Raman spectroscopy provides information that is complementary to FT-IR spectroscopy. In the present case, the most relevant information concerns the G and D bands, at about 1590 and 1350 cm^–1^, respectively. These bands serve as a primary indicator of the material structural integrity and quality, which may be significant in relation to the electrochemical properties.^76^ In particular, the D band is associated with the extent and number of defects within the graphitic regions. For materials **Ib**, **II**, and **IV** (clicked biochar), no significant changes in the appearance of these bands were observed (Figure 3), indicating that the reactions did not significantly affect the biochar structure by increasing its defectiveness. In the 1100–1400 cm^–1^ region, of the spectrum of the material **IV** (clicked biochar), bearing an alkyl chain, small peaks are observed at 1160 and 1330 cm^–1^, which are consistent with the C=N stretching mode of the triazole ring,^77,78^ further confirming the successful covalent immobilization of the alkyl chain via the alkyne unit.

**Figure 3:**
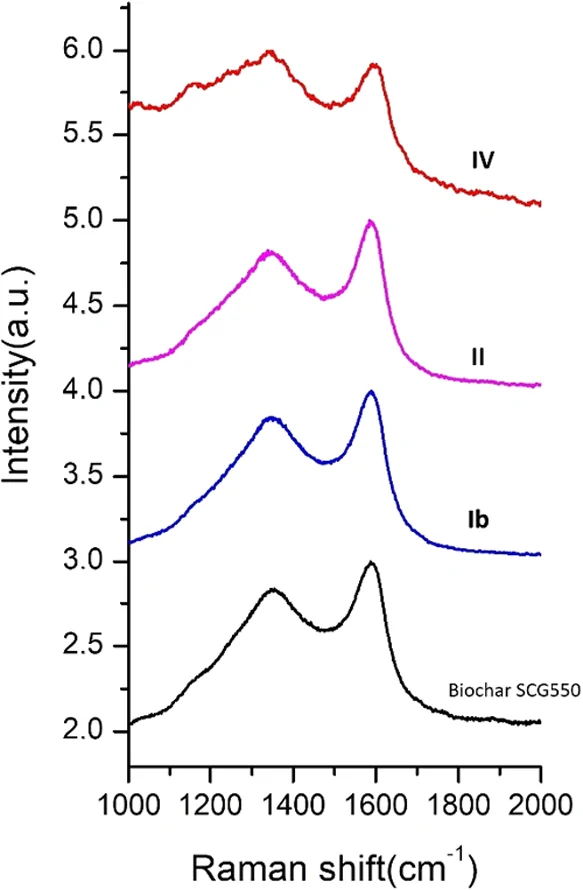
Raman spectra of the indicated materials showing the region of the D and G bands, typically associated with sp^2^ carbon materials.

Scheme 2 shows the introduction of the C_10_ alkyl chain on material **IV** (clicked biochar), selected among all modified materials, to be used to immobilize an enzyme (in this study, alkaline phosphatase, AP, was selected as an example).

### Electrochemical Measurements

The electrochemical performance of SCG550 (pristine biochar) and clicked biochar has been assessed through cyclic voltammetry (CV) and electrochemical impedance spectroscopy (EIS) using the ferri/ferrocyanide couple as a redox probe. Before biochar deposition, the SPCEs were electrochemically activated applying a constant potential of 1.7 V for 180 s in PBS. The measurements show that the electrodes modified with pristine SCG550 and clicked biochar showed comparable electrochemical responses. Pristine SCG550 displayed anodic and cathodic peak currents of 57.0 ± 0.6 and −51 ± 1 μA, respectively, with a peak-to-peak separation of 0.340 V. Clicked biochar showed slightly higher peak currents, 64 ± 2 and −55 ± 2 μA, and a lower peak-to-peak separation of 0.260 V. The EIS results showed similar charge transfer resistance values for the two materials, with *R*_ct_ = 3.3 ± 0.1 kΩ for pristine SCG550 and *R*_ct_ = 3.02 ± 0.08 kΩ for clicked biochar (see Figure 4).

**Figure 4:**
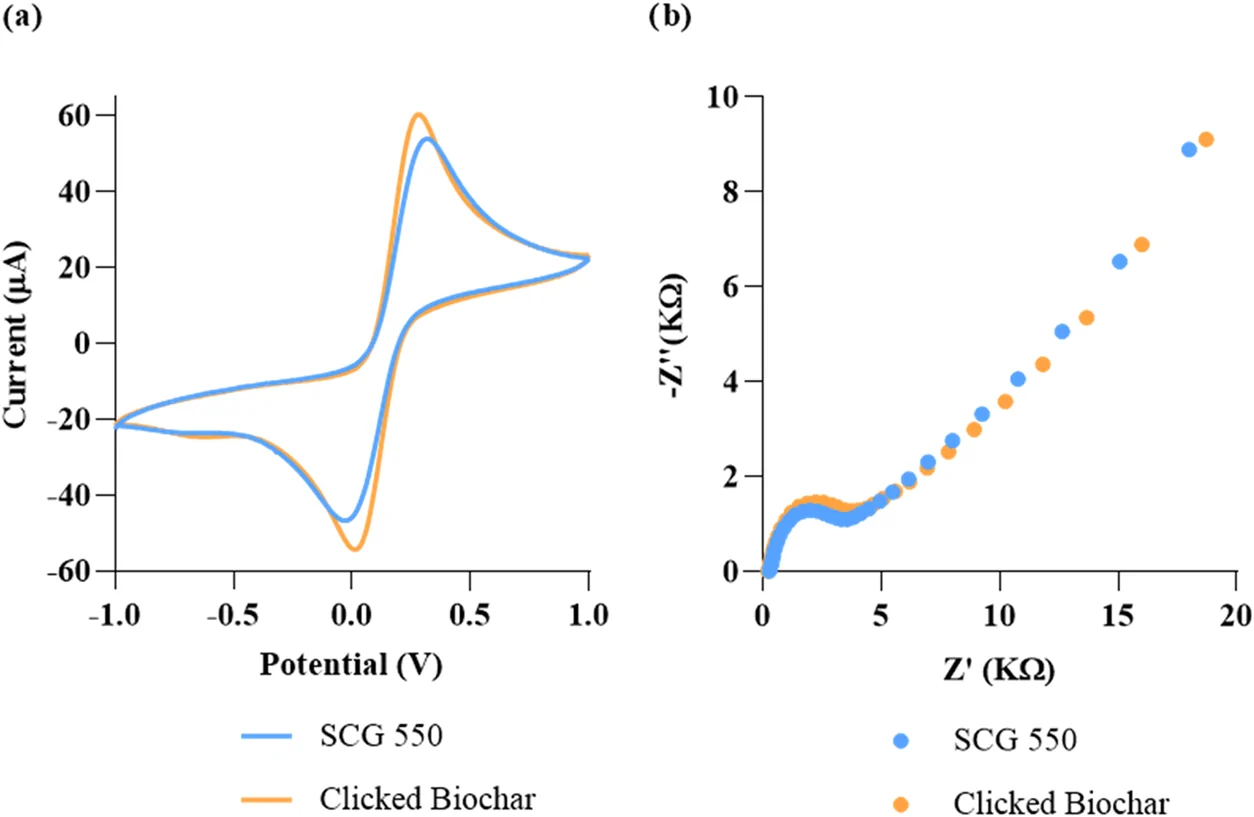
Electrochemical characterization of electrodes treated with biochar SCG550 and clicked biochar. Cyclic voltammograms (a) and electrochemical impedance spectroscopy (b) Nyquist plots recorded in 10 mM ferri/ferrocyanide redox probe and 0.1 M KCl electrolyte.

These results indicate that click functionalization does not change the intrinsic electrochemical performance of biochar. Rather, the clicked material preserves an electrochemical response comparable to that of pristine SCG550, with a slightly improved voltammetric behavior while introducing carboxyl-terminated molecular spacers suitable for subsequent enzyme immobilization. Therefore, the main role of the proposed functionalization strategy should not be interpreted as the preparation of a superior conductive carbon substrate but as the development of a chemically engineered biochar interface for improved biofunctionalization.

The electrochemical performance of SCG-derived biochar-modified SPEs, including the influence of pyrolysis conditions, surface morphology, dispersion medium, and electrode preparation, has been investigated in detail in a previous dedicated study.^59^ In the present work, the focus is instead placed on the comparison between pristine SCG550 and its click-functionalized derivative under identical electrode preparation and measurement conditions.

### Evaluation of Enzyme Immobilization

Following electrode modification with clicked biochar, i.e., material **IV**, the electrodes were further treated, as depicted in Scheme 2, through enzyme immobilization to evaluate the improved performance and potential advantages of this material in providing a more accessible interfacial environment for the immobilized enzyme. It is important to note that due to the use of EDC/NHS chemistry, enzyme immobilization in this system is inherently random, as it occurs through multiple accessible amine groups on the protein surface. Therefore, the role of the spacer is not to control enzyme orientation but to mitigate its effects by increasing enzyme–surface separation and conformational freedom. To assess bioreceptor immobilization efficiency, which is the covalent attachment of the alkaline phosphatase (AP) enzyme, electrochemical impedance spectroscopy (EIS) was employed. A range of enzyme concentrations of 0, 0.5, 1, 3, 5, and 10 U/mL in solution (using an AP stock solution with a supplier-declared activity of 37 U mg^–1^) was evaluated on both unmodified biochar (SCG) and clicked biochar. Using [Fe­(CN)_6_]^3–/4–^ as a redox probe, by extracting charge transfer resistance (*R*_ct_) values through Randles equivalent circuit fitting (Figure S5.1) and normalizing them against each electrode baseline (corresponding to 0 U/mL enzyme concentration) resistance (*R*_ct_/*R*_ct,0_), enzyme binding effects on surface resistance could be directly compared across different materials. It should be noted that EIS does not provide an absolute quantification of enzyme loading. Rather, normalized charge transfer resistance values were used to compare the AP-induced interfacial response of pristine SCG550 and clicked biochar under identical immobilization conditions. All the impedance spectra used were checked by linear Kramers–Kronig validity tests, and representative Nyquist plots are reported in the Supporting Information (Figures S5.2 and S5.3).

Following AP immobilization, the two materials showed noticeably distinct EIS trends, as shown in Figure 5. *R*_ct_/*R*_ct,0_ for pristine SCG550 very slightly increased at low AP doses, reaching a modest plateau at 3 U/mL before declining at 10 U/mL. This trend implies that increasing the AP concentration in the immobilization solution does not result in a corresponding increase in the interfacial enzyme-related reaction on the unmodified biochar surface. This can be explained by the restricted accessibility and availability of reactive surface sites, steric crowding, or inefficient enzyme layer organization.

**Figure 5:**
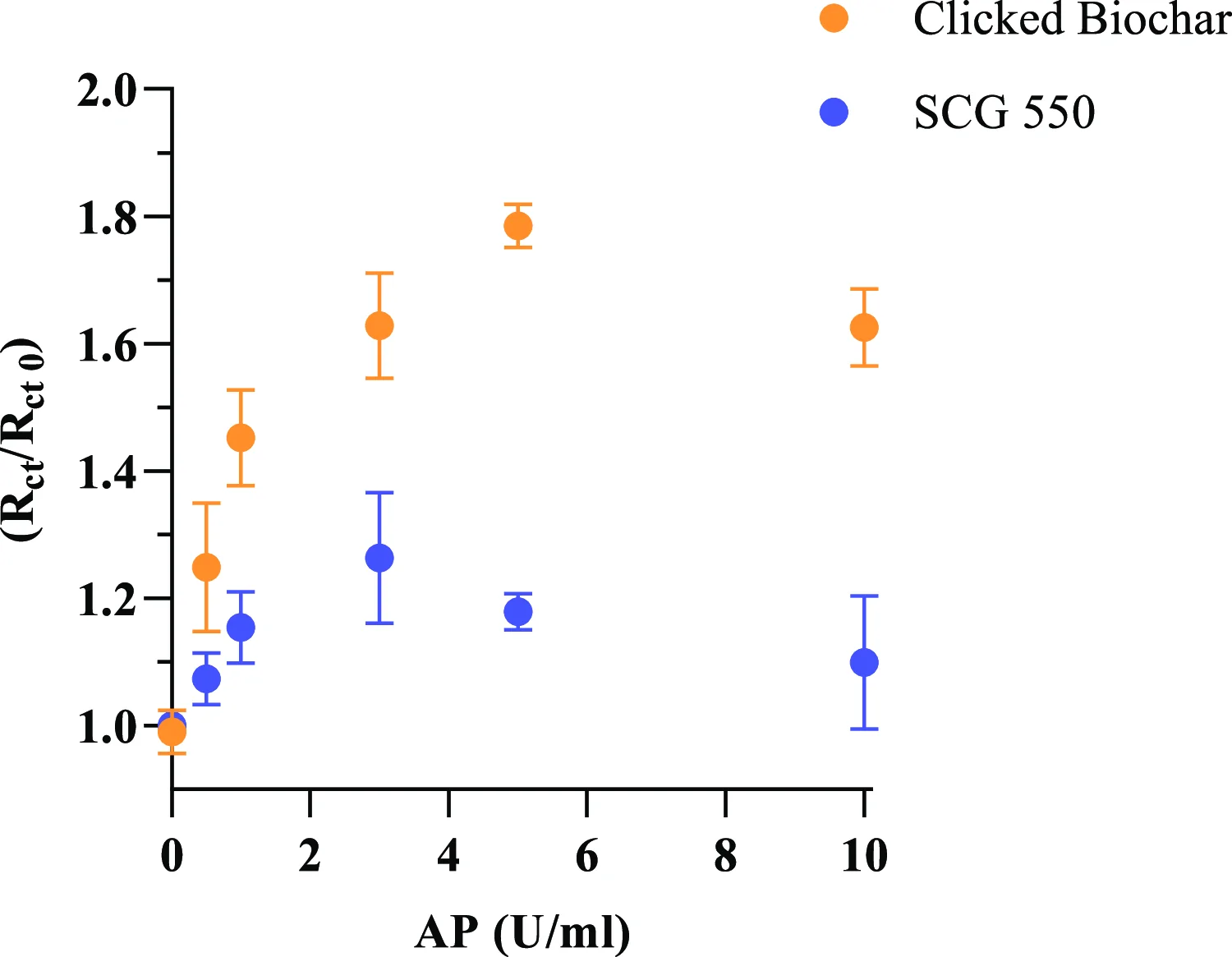
Normalized charge transfer resistance values (*R*_ct_/*R*_ct,0_) obtained by EIS as a function of the AP concentration used during immobilization (0, 0.5, 1, 3, 5, and 10 U/mL) on pristine SCG550 and clicked biochar electrodes. *R*_ct_ values were normalized against the corresponding 0 U/mL AP baseline for each material. Error bars represent standard deviation (*n* = 3).

In contrast, clicked biochar showed a much more pronounced increase in *R*_ct_/*R*_ct,0_ as the AP concentration increased from 0.5 to 5 U/mL, reaching the highest response at 5 U/mL. At 10 U/mL, the normalized *R*_ct_ decreased, indicating that high enzyme concentrations may result in an unfavorable interfacial arrangement of partial immobilized protein layer crowding or the hook effect. Overall, this trend indicates that the clicked spacer architecture allows the electrode interface to respond more effectively to increasing AP concentrations, with an optimal AP concentration of 5 U/mL under the investigated conditions.

To support the EIS interpretation with an independent method, BCA (bicinchoninic acid) protein quantification (spectrophotometric assay, carried out using a 96-well microplate) was performed after the same immobilization and washing procedure (see Figure S5.4). The BCA assay confirmed the same qualitative trend observed by EIS, showing higher AP retention on clicked biochar than on pristine SCG550, particularly at intermediate to high AP concentrations. As expected, the difference between the two materials clearly was more resolved by EIS because EIS is highly sensitive to local interfacial changes affecting charge transfer.

The immobilization trend was further evaluated by fixed-substrate chronoamperometric measurements using 5 mM 1-NPP (see Figure S5.5). The catalytic current increased with the AP concentration used during immobilization for both of the materials. However, clicked biochar consistently produced higher average current responses than pristine SCG550 at each AP concentration tested. This result indicates that the higher immobilization-associated response observed by EIS is accompanied by an increased functional enzymatic response. As a result, the clicking spacer design encourages the creation of an enzyme layer that is still catalytically accessible, in addition to protein retention at the surface.

All things considered, these additional findings show that clicked biochar offers a better interface than pristine SCG550 for AP immobilization. This improvement should be seen as a comparative electrochemical, colorimetric, and functional assessment of AP retention and immobilization-associated interfacial effects rather than an absolute quantification of enzyme loading by EIS.

### Catalytic Performance Evaluation

To evaluate catalytic performance, AP (1 U/mL) was immobilized on both biochar surfaces (SCG550 and clicked biochar, respectively) and tested through chronoamperometric measurements with different concentrations (0–0.1–0.25–0.5–1–2–5–7.5–10 mM) of 1-naphthyl phosphate (1-NPP) as a substrate (*n* = 3). After enzyme attachment via an EDC/NHS approach and ethanolamine surface blocking (see the Experimental Section), chronoamperometric responses were recorded over 2 min at the optimized potential. The steady-state current at 120 s provided an analytical signal for kinetic analysis.

Current responses were analyzed against different substrate concentrations using the standard Michaelis–Menten relationship *I* = *I*_max_ [*S*]/(*K*_m_ + [*S*]), after blank subtraction (0 mM 1-NPP) as depicted in Figure 6. *I*_max_ is the apparent maximum catalytic current, [*S*] is the 1-NPP concentration, and *K*_m_ is the apparent Michaelis–Menten constant. The kinetic parameters obtained from the fit were considered apparent parameters, as commonly done for surface-confined enzymatic systems. In electrode-based biosensors, the measured response is influenced not only by the intrinsic enzyme kinetics but also by substrate diffusion, interfacial mass transport, enzyme orientation, surface accessibility, and electron transfer-related processes at the electrode interface.

**Figure 6:**
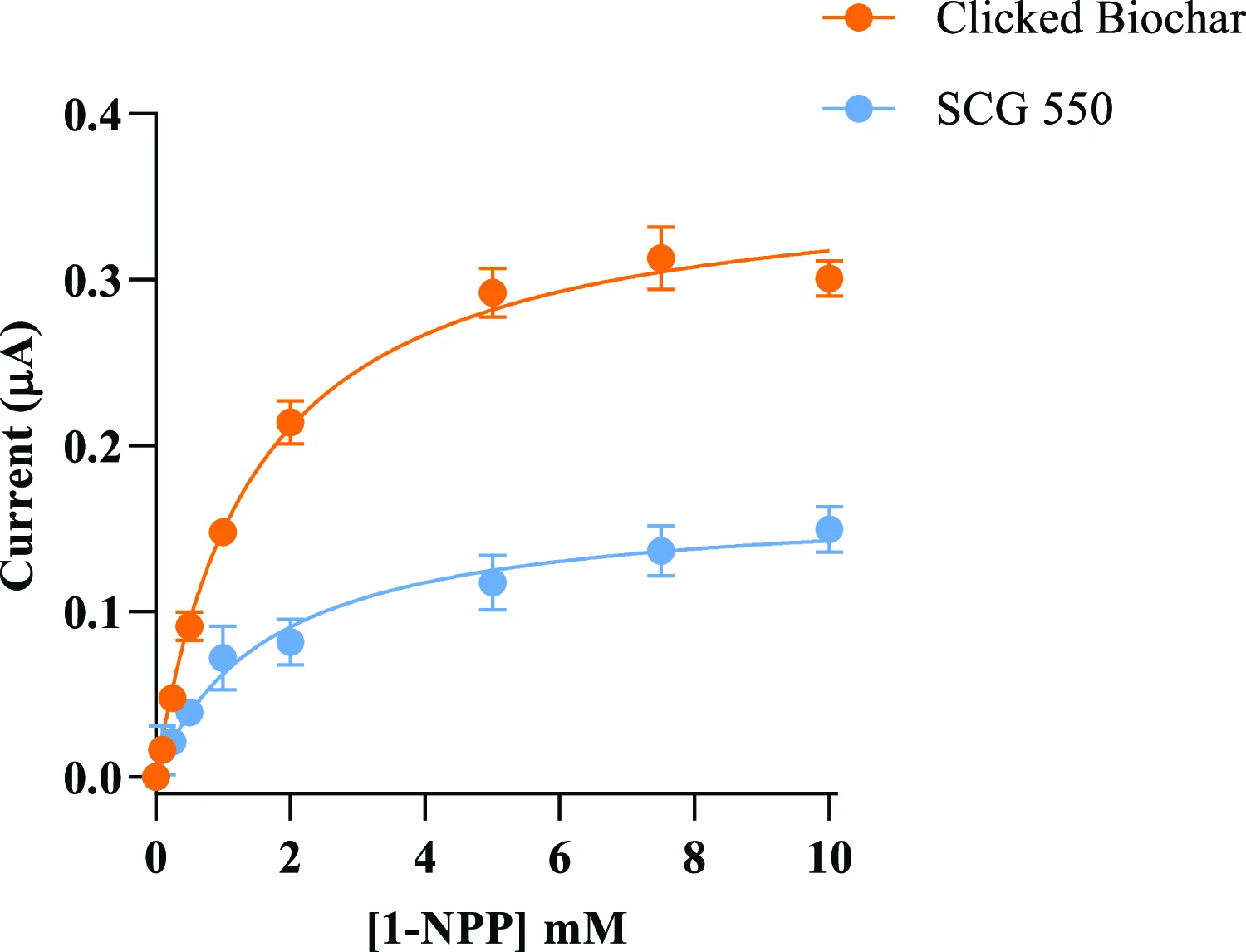
Michaelis–Menten kinetic curves for immobilized alkaline phosphatase on unmodified SCG biochar and material **VI**. Experimental conditions are described in the Experimental Section.

As shown in Figure 6, Material **VI** displayed a markedly higher catalytic response than pristine SCG550 over the whole substrate concentration range. The fitted *I*_max_ value increased from 0.167 μA for pristine SCG550 to 0.363 μA for clicked biochar, corresponding to an approximately 2.2-fold enhancement in the apparent maximum catalytic current.

In contrast, the apparent Michaelis–Menten constants were comparable within the confidence intervals, with *K*_m_ = 1.68 mM for SCG550 and *K*_m_ = 1.45 mM for Material VI. Therefore, the improved response of clicked biochar should not be interpreted as a major change in the apparent substrate affinity or in the intrinsic enzyme kinetics. Rather, the higher *I*_max_ indicates that a larger fraction of immobilized AP remains functionally accessible and able to contribute to the electrochemical signal.

These results align with the previously discussed EIS, BCA, and fixed-substrate amperometric data. The clicking spacer architecture facilitates the creation of an enzyme layer that is more efficiently accessible to the substrate and electrochemical transduction, as well as AP immobilization. Overall, clicked biochar enhances the immobilized AP layer’s functional catalytic response without significantly changing the apparent substrate affinity/transport regime, according to the Michaelis–Menten analysis.

### Antifouling Ability Evaluation

The antifouling characteristics of functionalized biochar electrodes were evaluated through an evaluation of their resistance toward nonspecific protein adsorption. After immobilizing enzymes (1 U/mL AP) and surface blocking with ethanolamine, a solution of 1% (w/v) bovine serum albumin (BSA) was incubated for 1 h on material **VI** and SCG550 electrodes. EIS measurements were performed in a 10 mM [Fe­(CN)_6_]^3–/4–^ solution before and after BSA exposure, and *R*_ct_ values were determined by fitting Randles equivalent circuits to Nyquist plots.

The relative increase in *R*_ct_ appears in Figure 7, calculated according to eq 1

1 $$increase\;R_{ct}\;\left(\%\right)=\frac{R_{ct,after\;BSA}-R_{ct,before\;BSA}}{R_{ct,before\;BSA}}$$

**Figure 7:**
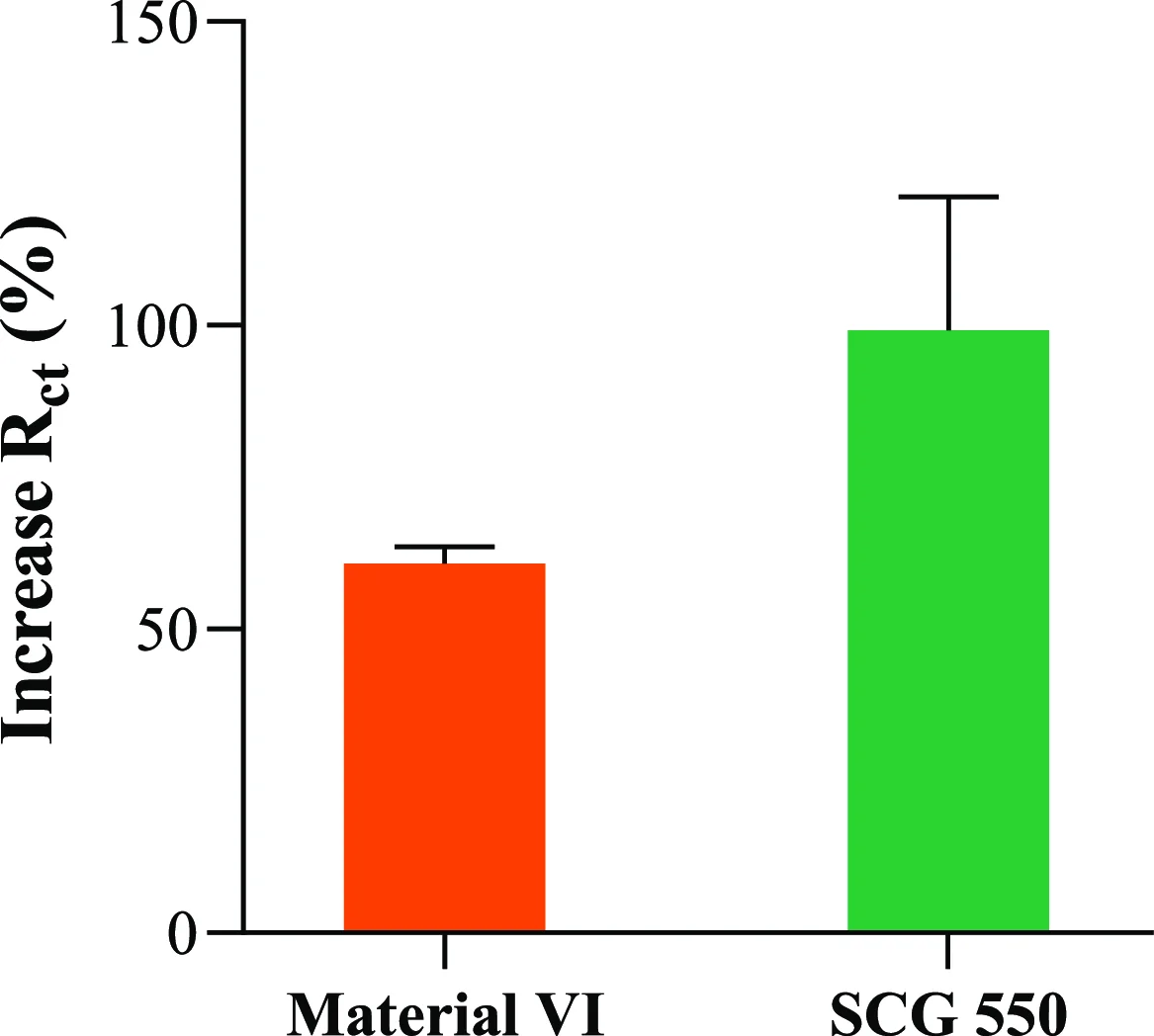
Relative charge transfer resistance increase (*R*_ct_) values (%) for unmodified SCG biochar and material VI electrodes following 1 h incubation in 1% (w/v) BSA solution (*n* = 3).

Figure 7 shows that unmodified SCG electrodes exhibited an *R*_ct_ increase of 99 ± 22%, after BSA treatment, demonstrating significant surface blockage from nonspecific BSA adsorption. The electrodes treated with materials **VI** displayed considerably reduced nonspecific adsorption, with only a 61 ± 3% increase in resistance representing a 39% fouling reduction relative to biochar SCG (*n* = 3).

The antifouling property can be attributed to two distinct factors: (i) the molecular spacer creates steric barriers that prevent large protein adsorption and (ii) the free alkyne-terminated groups and ethanolamine capping generate more hydrophilic surface characteristics, which lead to reduced protein adhesion. These modifications reduce the hydrophobic and electrostatic attraction that typically fuels fouling processes in carbon-based sensing platforms.^79^

Reducing fouling not only preserves the electroactive surface area necessary for optimal electron transfer kinetics but also could enhance measurement precision under physiologically relevant conditions, a critical factor for sensor operation in complex biological matrices.

## Conclusions

This work presents a comprehensive strategy for the chemical functionalization of biochar from fast pyrolysis of lignocellulosic residual biomass through a multistep synthetic approach, successfully addressing fundamental limitations that have hindered the widespread application of biochar in biosensing platforms. By introducing a covalently bound C_10_ alkyl spacer via azide–alkyne cycloaddition, we have obtained a reproducible method to enhance both the analytical performance and practical utility of biochar-based electrochemical biosensors.

The systematic characterization through spectroscopic, thermal, and electrochemical techniques confirms successful functionalization at each synthetic step while preserving the quasi-graphitic structure essential for electrochemical activity. Most importantly, the spacer-mediated strategy overcomes the longstanding trade-off between electrochemical performance and biofunctionalization capacity that has constrained the application of biochar in high-sensitivity analytical systems.

The final material showed improved performance compared to pristine SCG biochar in the enzymatic model system investigated here. EIS measurements revealed a more pronounced AP immobilization-associated interfacial response for clicked biochar, and this trend was independently supported by BCA protein quantification. Fixed-substrate amperometric measurements further confirmed that the increased AP retention translated to a higher functional enzymatic response.

Chronoamperometric Michaelis–Menten analysis showed an approximately 2.2-fold increase in the apparent maximum catalytic current for clicked biochar compared with pristine SCG biochar, while the apparent Michaelis–Menten constants remained comparable. These results indicate that the spacer-functionalized architecture improves the functional accessibility of immobilized AP without substantially altering the apparent substrate affinity or the overall reaction–transport regime.

The electrochemical evaluation of BSA-induced fouling also showed a lower charge transfer resistance increase for clicked biochar than for pristine SCG biochar, suggesting that the spacer-functionalized interface is less affected by nonspecific protein adsorption from an electrochemical point of view. This result should be interpreted as a comparative electrochemical index of fouling-induced interfacial blocking rather than as an absolute quantification of the adsorbed protein.

The use of abundant, low-cost coffee ground-derived biochar further enhances the economic viability of this approach for scalable biosensor production.

Overall, the proposed click functionalization strategy provides a chemically engineered biochar interface that combines suitable electrochemical behavior with improved enzyme immobilization and a functional catalytic response. The versatility of the spacer-based approach may enable its extension to other biorecognition elements, including enzymes, antibodies, and aptamers, supporting the development of sustainable biochar-based electrochemical biosensing platforms.

## Experimental Section

### Materials

The reagents employed in the preparation are commercially available and they were used with no further purification. The solvents used in the experiments were analytical grade. Ultrapure mQ water was used for all preparation steps illustrated in the present work. The original feedstock (spent coffee grounds) was collected from the cafeteria of the Industrial Engineering Department of the University of Rome Tor Vergata. Before the pyrolytic conversion, the raw feedstock was dried in a static oven for 12 h at 105 °C. A lab-scale screw-type reactor was employed for the fast pyrolysis experiments at 550 °C. Further details about the fast pyrolysis experiments can be found in previous investigations.^59,80,81^

A common colorimetric technique for figuring out the total amount of protein in a solution is the BCA (bicinchoninic acid) assay (Pierce, ThermoFisher Scientific, Milan, Italy). The analysis was performed according to the kit instructions for a few μL of samples.

Screen-printed electrodes (SPEs) were fabricated in-house at the University of Rome Tor Vergata using a DEK 245 screen-printing machine (Weymouth, U.K.) with a graphite working and counter electrode and a silver/silver chloride reference electrode. The graphite-based working electrode had 0.07 cm^2^ area.

### Instruments and General Methods

The FT-IR spectra were recorded using a pellet prepared by mixing 2–5 mg of powder with 100–120 mg of KBr. The acquisitions of the spectra were carried out using a Thermo Scientific instrument (model Is50) (Thermo Scientific Inc., Madison, WI USA). The spectra were elaborated with software OMNIC 9.16.159. The sonication of the carbon-based materials was carried out with the following instrument: ultrasonic UTA-35, power: 180 W, Falc srl BG, and probe sonicator (Hielscher UP200 St, Teltow, DE). The electrochemical measurements were carried out with a PalmSens4 instrument (PalmSens, Netherlands), and the data were elaborated with software PStrace version 5.11.

The SEM images were recorded with the following instrument: Zeiss Auriga 405, CNIS instrument infrastructure, La Sapienza.

The micro-Raman experiments, i.e., spectra and optical microscopy images, were carried out with a Horiba Scientific LabRam Odyssey instrument, X-Chem infrastructure, Tor Vergata.

All of the reactions were performed under a static nitrogen atmosphere and in a hermetically sealed flask.

### Preparation of Carbon-Based Materials

**Ia**: A solution was prepared by dispersing 1.01 g of biochar in 200 mL of nitric acid in a 500 mL three-neck round-bottom flask equipped with a magnetic stir bar and a splash guard. The mixture was sonicated for 1 h and then stirred for 4 h. The resulting suspension was vacuum-filtered and dried with a high-vacuum pump for 2 h, after thorough washing with water and methanol. Isolated material: 0.920 g.

**Ib** 200 mL of a solution of 0.1 M potassium permanganate was used to disperse 1.014 g of biochar. The suspension was sonicated for 1 h and then kept under stirring for 7 days at room temperature. Subsequently, the mixture was vacuum-filtered and dried using a high-vacuum oil pump for 3 h, after thorough washing with water and methanol. Isolated material: 0.765 g.

**II**: 1.03 g of **Ib** was added to a solution prepared by dissolution of 2.4 g of sodium azide in 2:1 v/v acetonitrile/water. The mixture was sonicated for 30 min and then refluxed for 7 days (temperature: 94 °C). Afterward, the reaction mixture was centrifuged at 10,000 rpm for 10 min. The solid was isolated from the supernatant and rinsed twice with the same solvent mixture. Isolated material: 0.890 g.

**III**: 0.202 g of **II** was dispersed in a solution prepared by addition of 4 mL of hydrazine monohydrate in 50 mL of water. The mixture, previously sonicated for 30 min, was stirred for 5 days at room temperature and subsequently refluxed for 24 h (temperature: 115 °C). Afterward, the precipitate was isolated via vacuum filtering and dried for 2 h under high vacuum after thorough washing with water and methanol. Isolated material: 0.154 g.

**IV**: 0.314 g of material **II** was dispersed in a 2:1 mixture of water and t-butanol and sonicated for 30 min. After that, 1.903 g of 10-undecynoic acid, 98 mg of ascorbate, and 23 mg of copper­(II) sulfate pentahydrate were added. The mixture was stirred for 14 days, then was vacuum-filtered, and washed three times with water and three times with dichloromethane. The isolated solid was dried for 4 h under high vacuum. Isolated material: 0.320 g.

### Preparation of Biochar Dispersions and Electrode Modification

Biochar powders were dispersed in an ethanol/water mixture (3:1, v/v) to yield a final concentration of 1 mg/mL. The suspensions were homogenized using a probe sonicator (Hielscher UP200 St, Teltow, DE) operated at 70–80% amplitude for 30 min, while maintaining the samples in an ice bath to prevent overheating or solvent evaporation.

Electrode modification was performed adding, by drop casting, 3 μL of the biochar suspension onto the SPE working electrode; this system was dried in an oven at 40 °C, and the process was repeated once (total 6 μL deposited). This procedure was used for both pristine and functionalized biochars.

### Enzyme Covalent Immobilization

#### Activation of Carboxyl Groups

SPEs modified with functionalized biochar were treated with an aqueous solution containing EDC (8 mM) and NHS (10 mM) in 100 mM MES buffer (pH 6.0) and incubated in the dark for 20 min at room temperature.

#### Enzyme Immobilization

Alkaline phosphatase (AP) was prepared at different concentrations in 10 mM PBS (Phosphate Saline Buffer) (pH 7.4). 6 μL was deposited on the working electrode and incubated overnight at 4 °C.

#### Blocking Step

To deactivate unreacted sites, 10 μL of 2% (w/v) ethanolamine solution in 10 mM PBS (pH 7.4) was added and incubated for 60 min at room temperature.

#### Washing

After each step, the electrode was rinsed three times with 50 μL of PBS buffer to remove unbound reagents.

### Enzymatic Assay

The enzymatic activity of immobilized AP was evaluated using 1-naphthyl phosphate (1-NPP) as a substrate. Measurements were performed in diethanolamine buffer (DEA, 0.97 M, pH 9.8) containing 0.08 M KCl and 1.0 mM MgCl_2_, which was freshly prepared before each experiment.

### Electrochemical Measurements

Electrochemical characterization was performed using ferri/ferrocyanide (5 mM K_3_[Fe­(CN)_6_] and 5 mM K_4_[Fe­(CN)_6_]) prepared in 0.1 M KCl. Cyclic voltammograms were recorded between −1 and +1 V at a scan rate of 50 mV/s. EIS was recorded over a frequency range of 10^5^–10^–1^ Hz, using 10 mV AC perturbation and a DC potential set at open-circuit potential. Chronoamperometric measurements were collected for 120 s at the optimized potential corresponding to the oxidation peak of the enzymatic substrate.

## Supplementary Material



## Notes

Additional comparative analysis of functionalization strategies for biosensing platform table, micro-Raman and scanning electron microscopy (SEM) images, electroanalytical measurements, and supporting discussions related to proposed click-functionalized biochar for electrochemical biosensing (PDF)
