# Supplementary material for: Click-Functionalized Biochar as a Spacer-Engineered Interface for Electrochemical Biosensing
Source: ACS Omega. 2026 Aug 14;11(33):49163–74. doi: 10.1021/acsomega.6c01575 (PMC13519924; doi:10.1021/acsomega.6c01575)
Supplement: Supplementary file 1 [file ao6c01575_si_001.pdf]

Supporting Information (SI)

Click-Functionalized Biochar as a Spacer-Engineered Interface for Electrochemical Biosensing

Antonio Licheri,[a] Veronica Mattarelli,[a] Lorenzo Bartolucci,[b] Rocco Cancelliere,[a] Claudia Mazzuca,[a] Pietro Mele,[b] Silvia Orlanducci,[a] Laura Micheli, [a,a]* Riccardo Salvioa,[a,]**

[a] Department of Chemical Science and Technologies, Tor Vergata University of Rome, Via Della Ricerca Scientifica 1, 00133, Roma, Italy

[b] Department of Industrial Engineering, Tor Vergata University of Rome, Via del Politecnico 1, 00133 Roma, Italy

* To whom the correspondence should be addressed laura.micheli@uniroma2.it, riccardo.salvio@uniroma2.it

Table of Contents

S1 Comparative Analysis of Functionalization Strategies for Biosensing Platforms…………..S2

S2 Scanning electron microscopy (SEM) images ……………………………………S3

S3 by energy-dispersive X-ray spectroscopy (EDX) experiments……………………………..S14

S4 Optical microscopy imaging from microRaman investigation ……………………………S18

S5 Electroanalytical Measurements ………………………………………………………...S23

S1 Comparative Analysis of Functionalization Strategies for Biosensing Platforms

Table S1. Comparison of biochar- with graphene/CNT nanomaterials and classical spacer-based immobilization strategies used to biosensing

Feature	Biochar	Graphene and Carbon Nanotubes	Spacer-based Immobilization (e.g., PEG, alkanethiols)
**Sustainability**	low-cost, renewable biomass by vegetable waste; environmentally sustainable.	synthetic production with limited sustainability.	depends on precursor chemistry.
**Surface Functionalization**	rich in groups containing oxygen; highly adjustable by pyrolysis conditions and biomass selection.	functionalization relies on defect engineering, oxidation, or π–π interactions; often heterogeneous	functional groups introduced using precisely defined chemistries but lack spatial control at the nanoscale.
**Spacer for probe**	tunable porosity and surface chemistry allow improved control over immobilization environment.	random distribution of reactive sites; limited control over probe orientation and density.	controlled pacer length is, but not probe spacing/orientation
**Reproducibility of modified interface**	high reproducibility with controlled pyrolysis parameters.	limited reproducibility by variability in defect density and oxidation patterns.	good chemical reproducibility
**Structural Tunability**	controlled by temperature, heating time and rate of pyrolysis, and biomass type	limited tunability; structure largely fixed by synthesis method.	limited tunability to terminal functional groups and molecular spacer length
**Porosity and Surface Area**	intrinsically porous improving the surface area	high surface area but non-porous unless modified	non-porous
**Cost**	very low cost	higher cost	moderate cost
**Suitability for Biosensing**	to facilitate improved signal transduction and customized immobilization conditions.	excellent conductivity but limited molecular-level control of biointerface.	To improve accessibility but lacks control needed for optimal hybridization

S2 Scanning Electron Microscopy (SEM) images

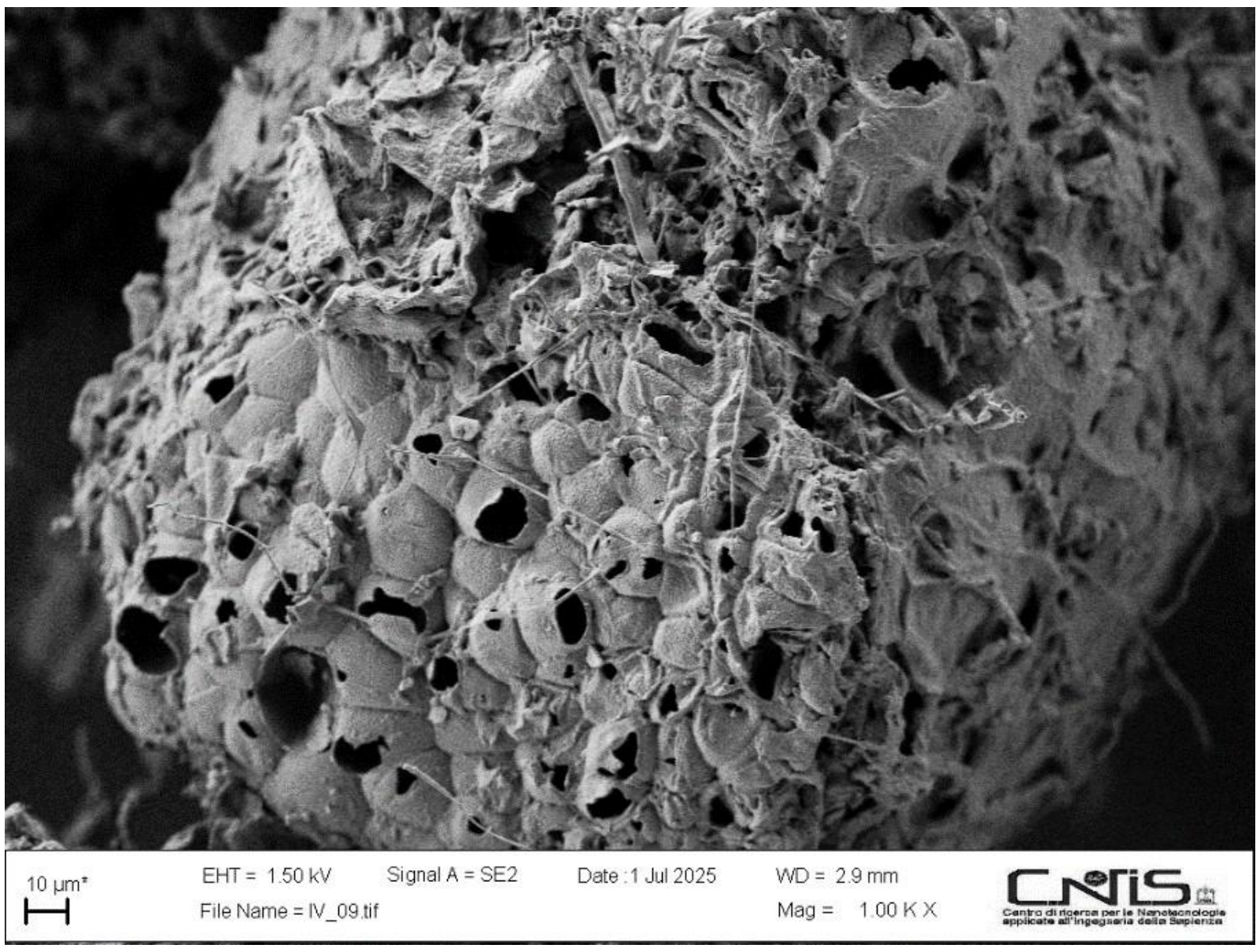

Figure S2.1 Biochar SCG550

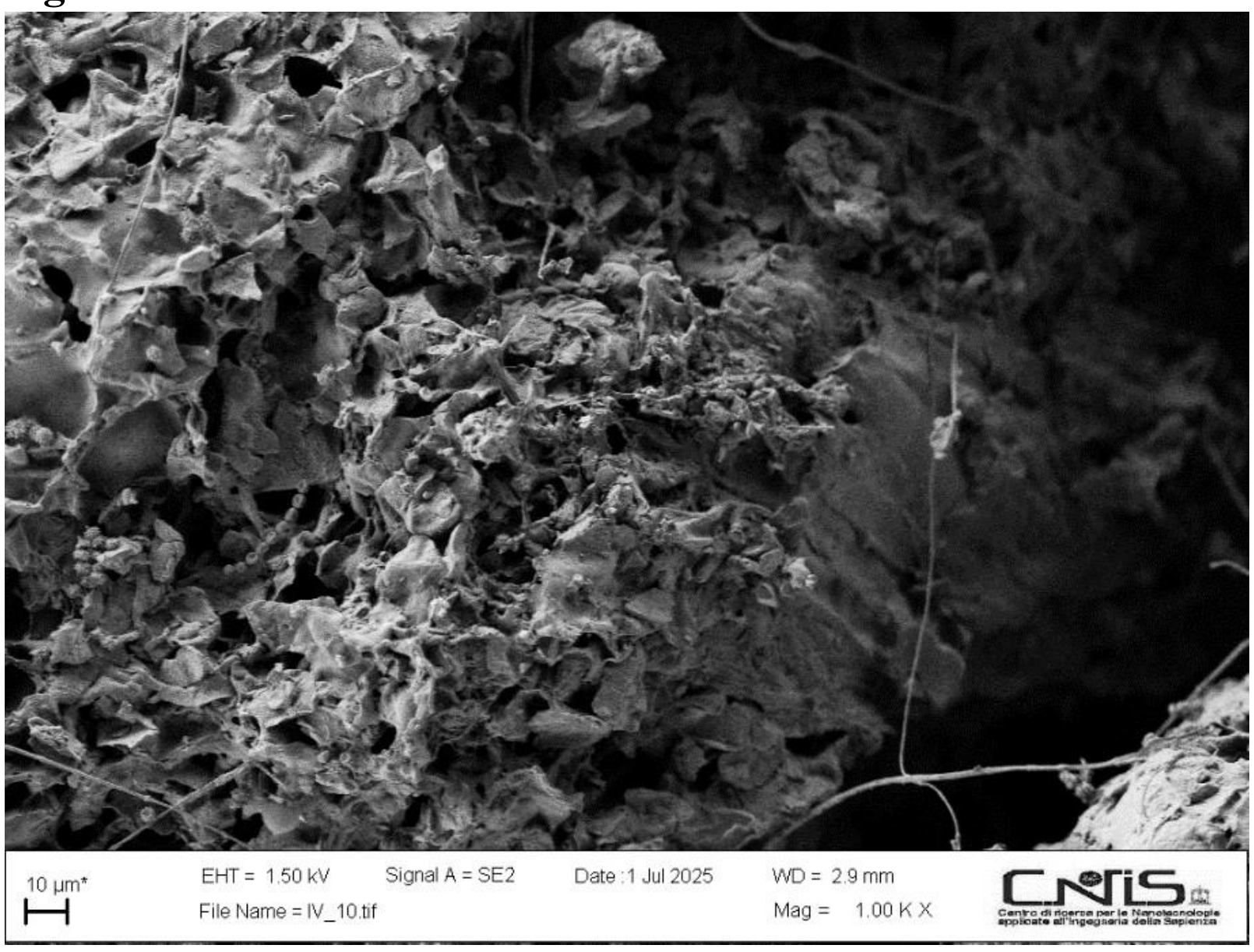

Figure S2.2 Biochar SCG550

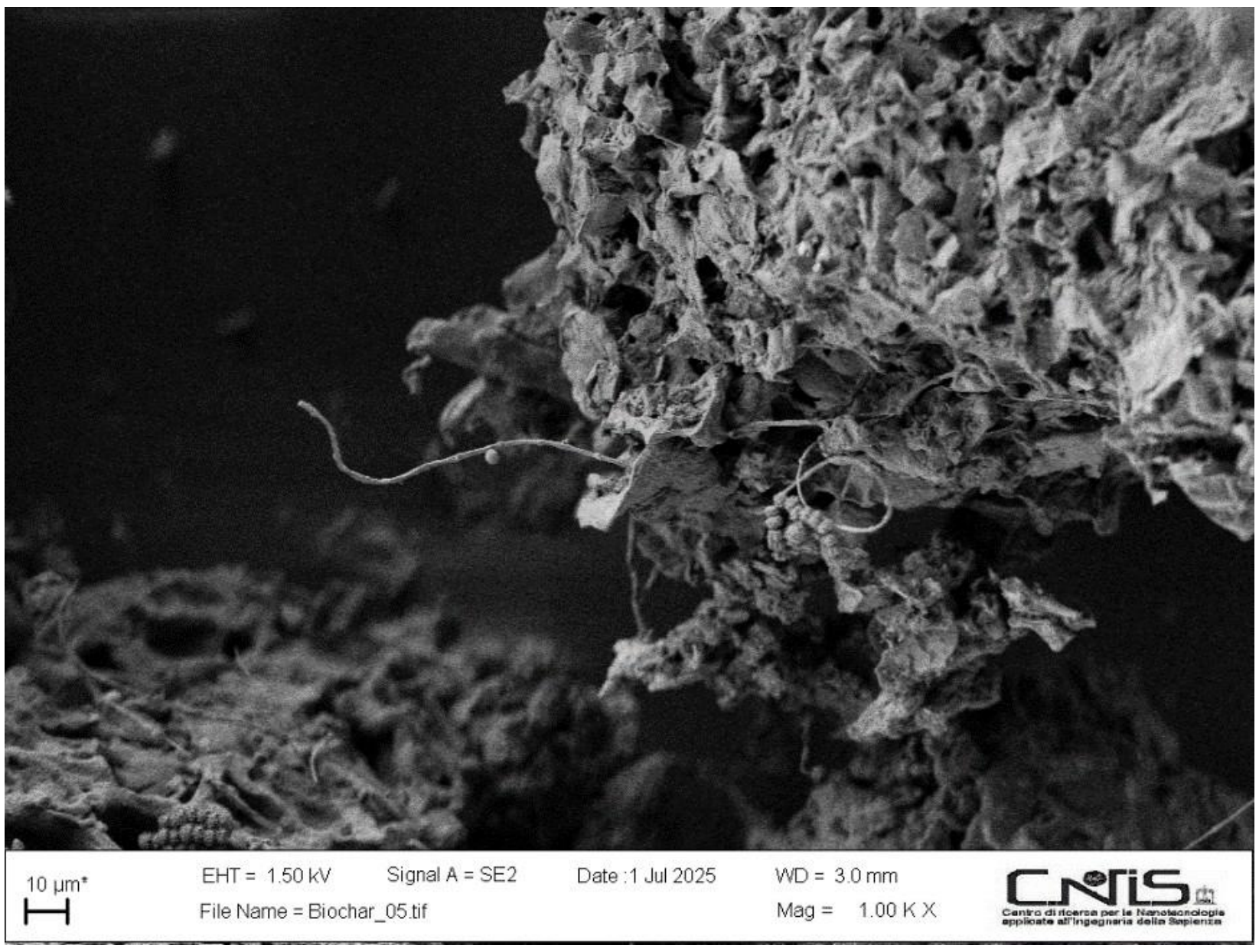

Figure S2.3 Biochar SCG550

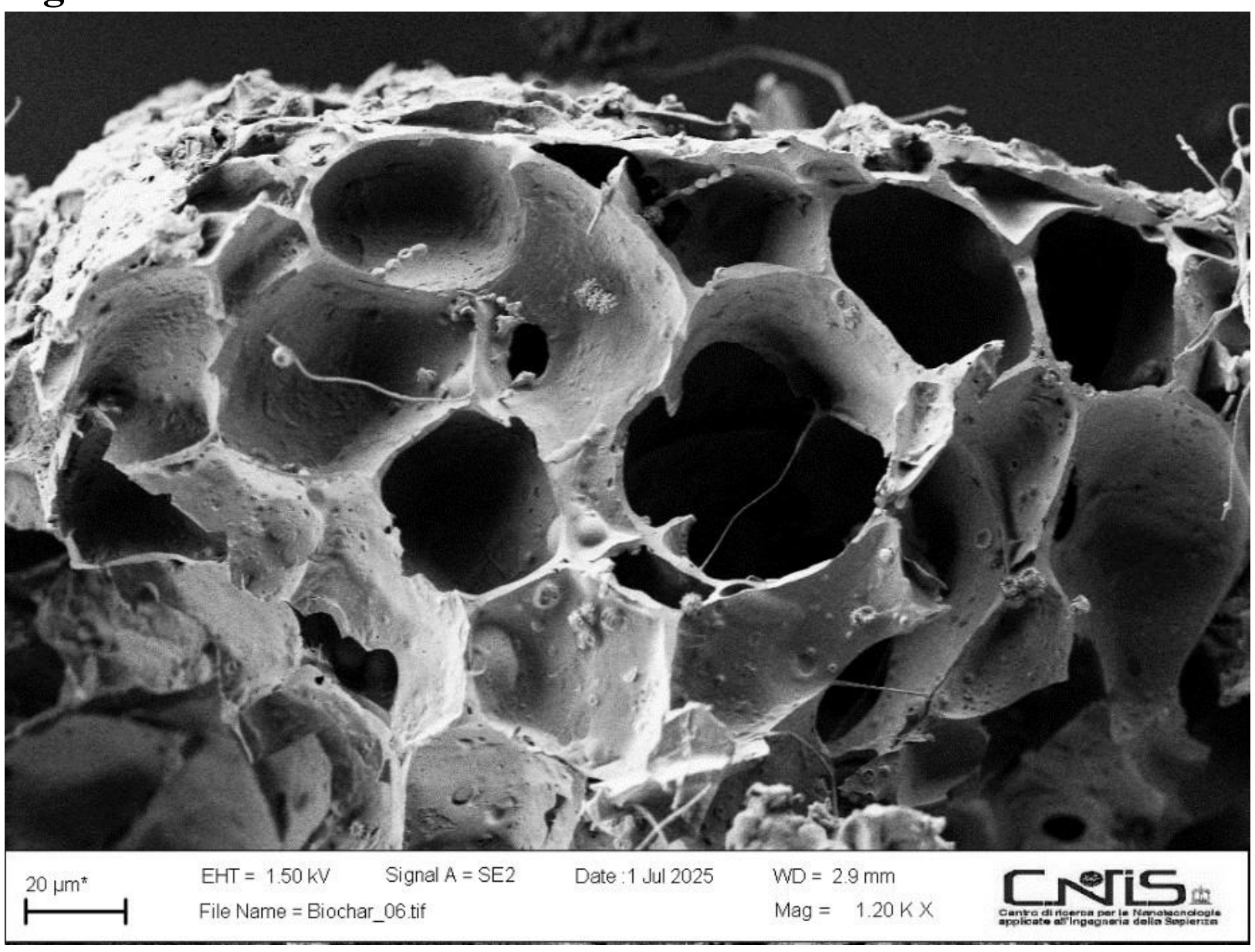

Figure S2.4 Biochar SCG550

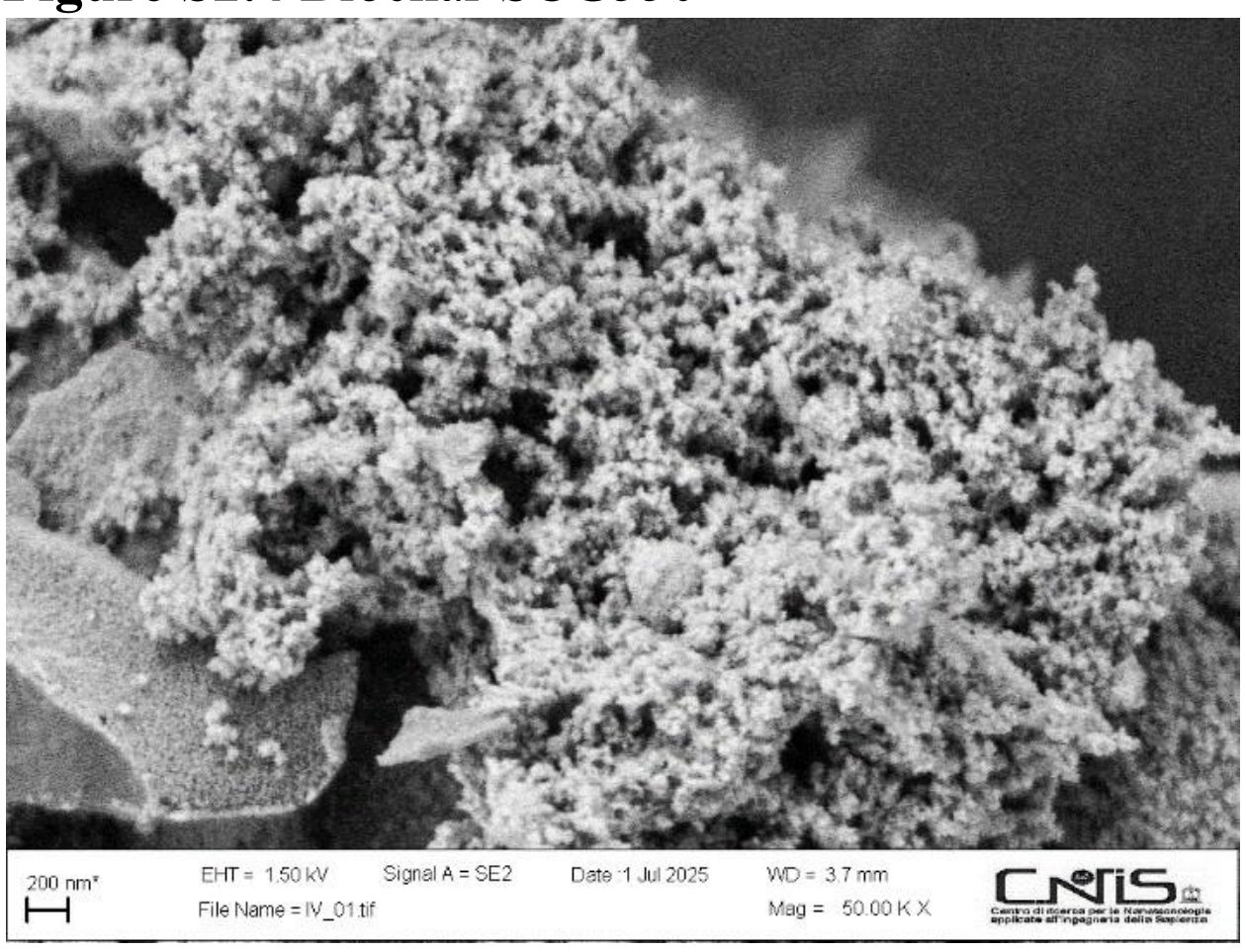

Figure S2.5 Material **Ib**

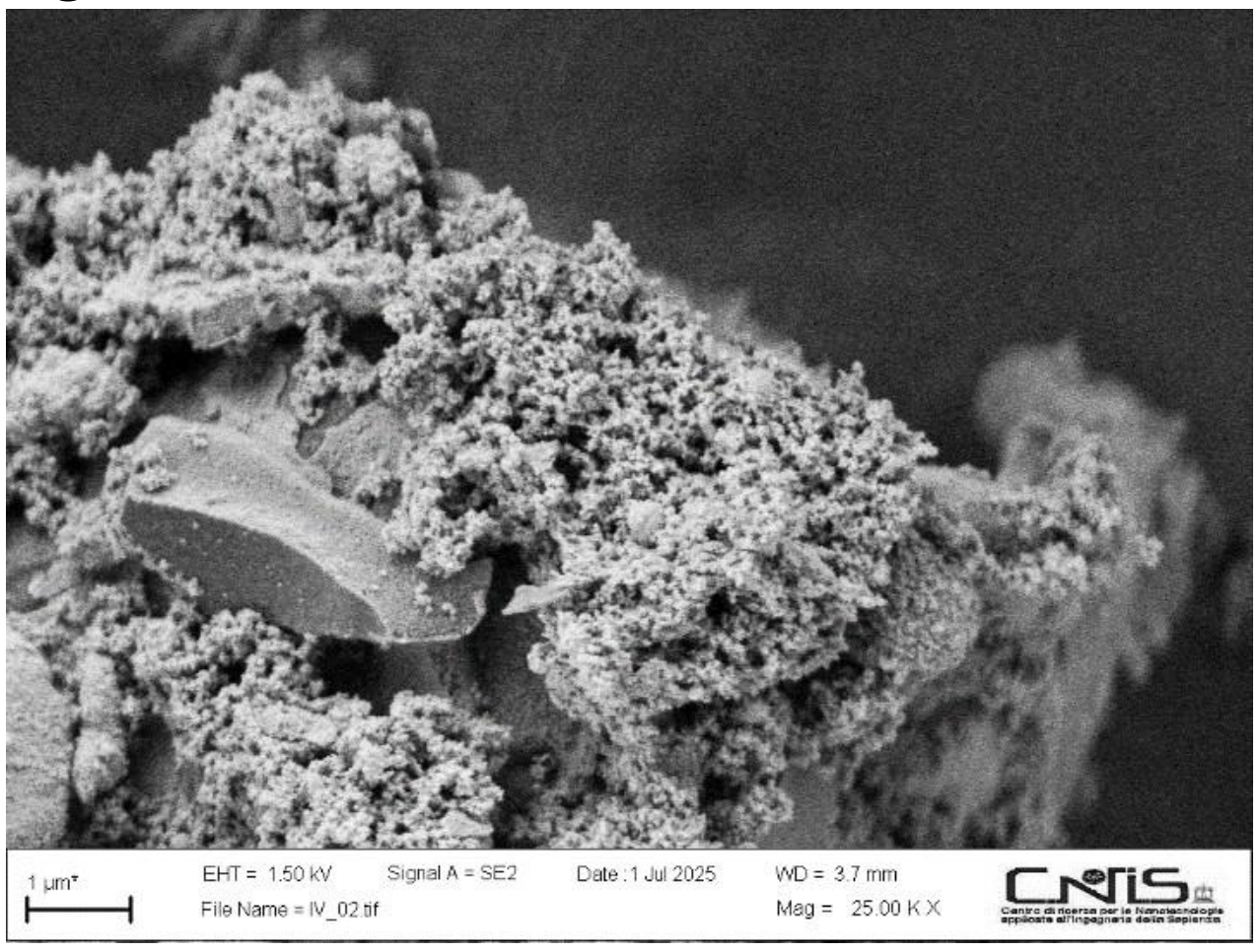

Figure S2.6 Material **Ib**

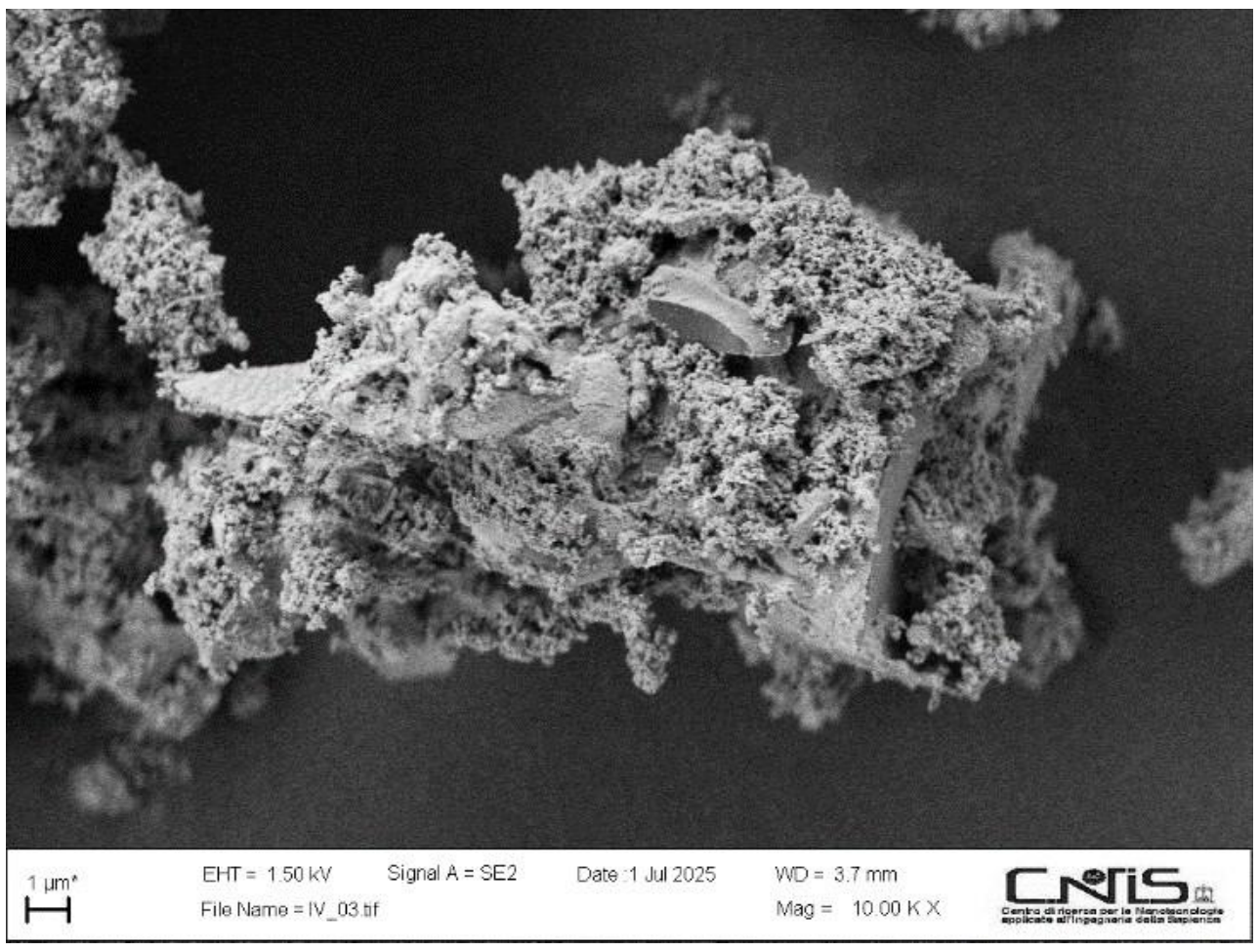

Figure S2.7 Material **Ib**

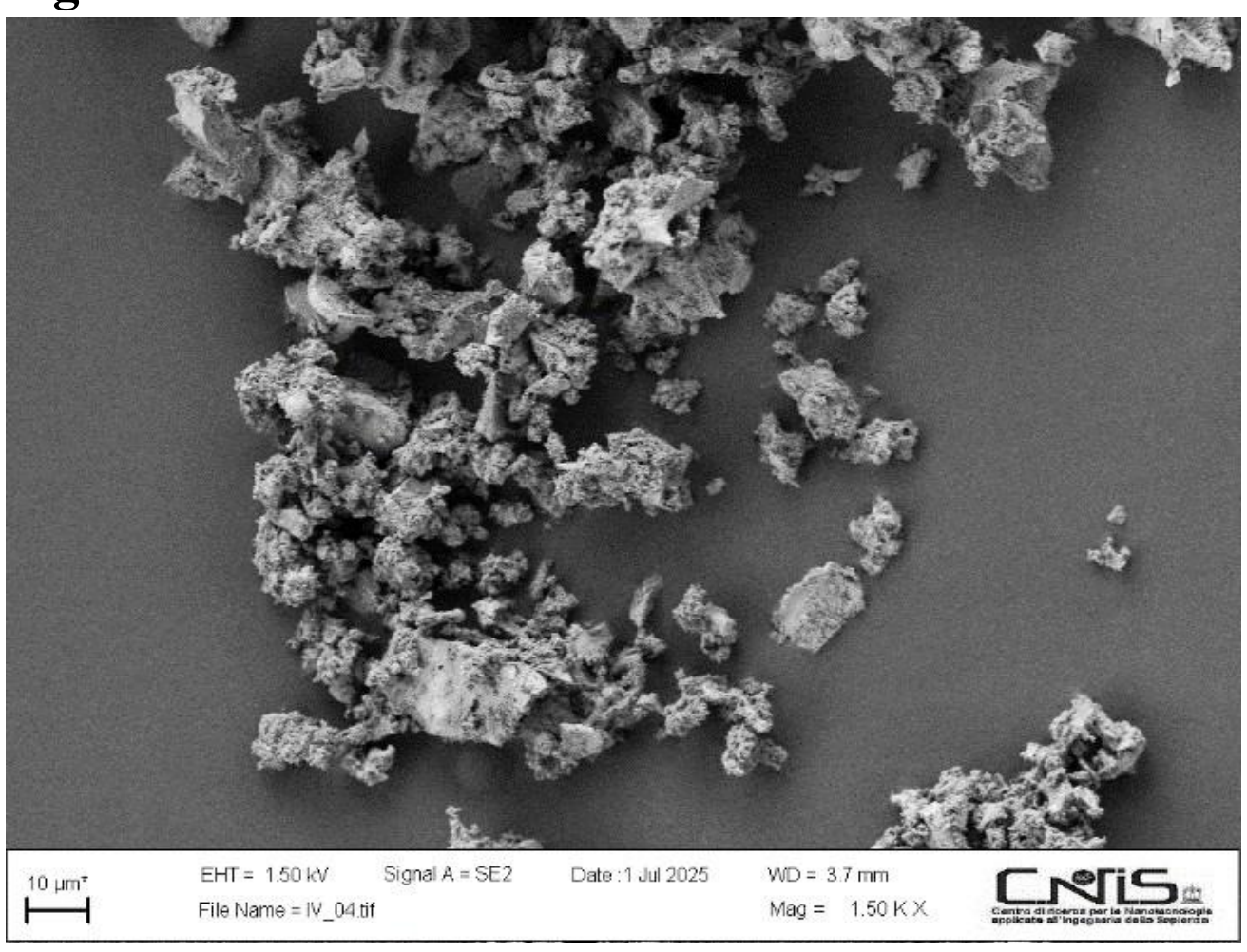

Figure S2.8 Material **Ib**

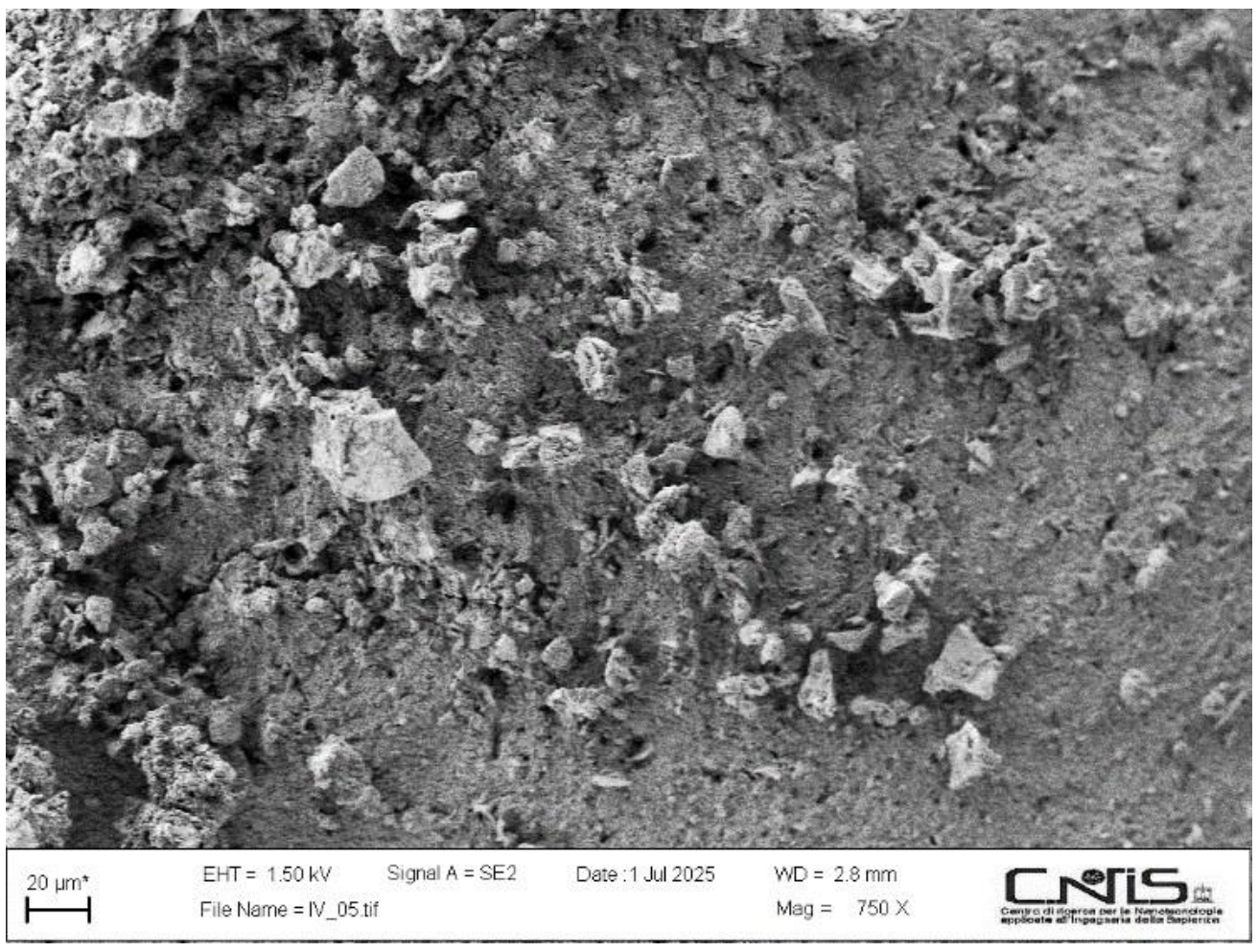

Figure S2.9 Material **Ib**

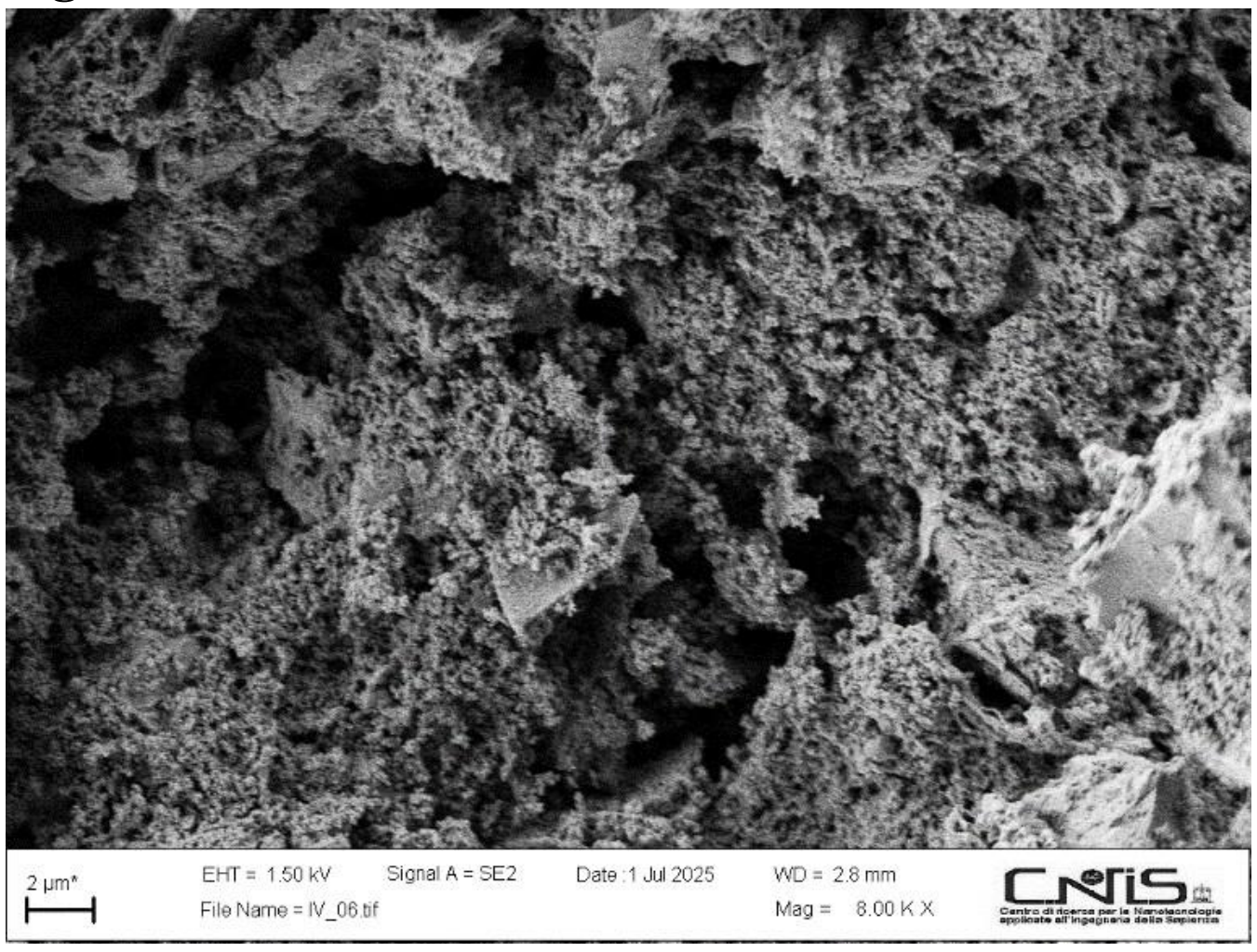

Figure S2.10 Material **Ib**

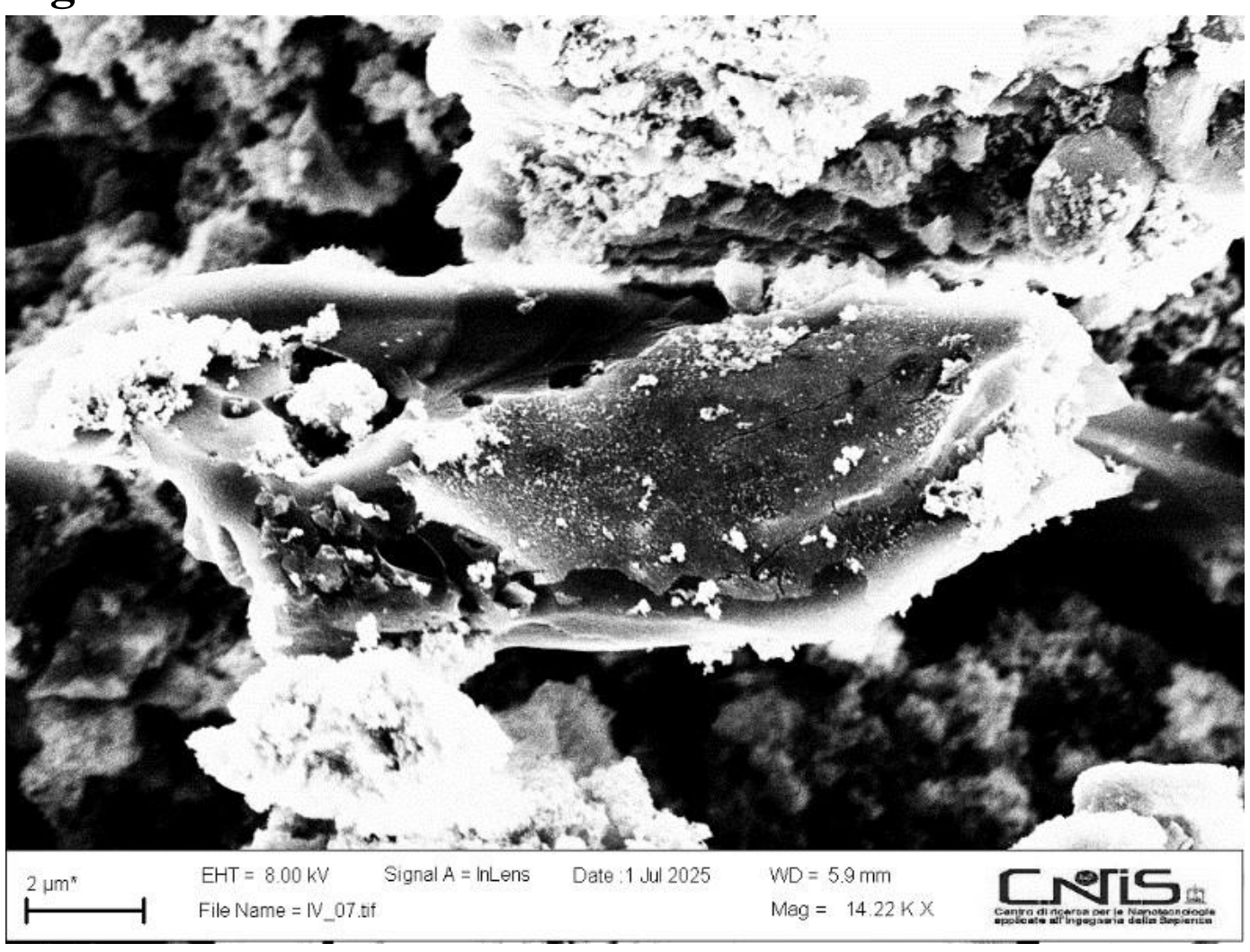

Figure S2.11 Material **Ib**

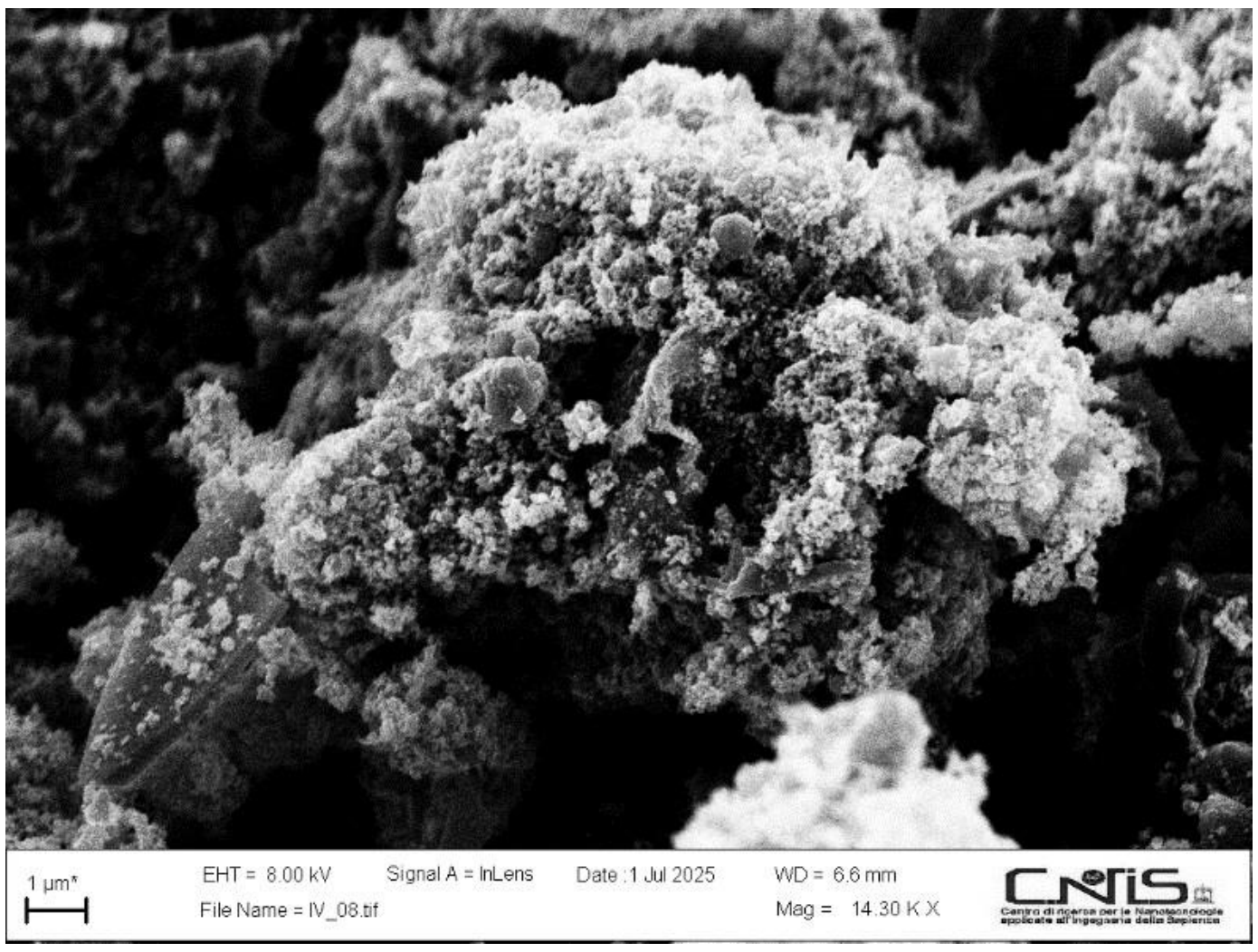

Figure S2.12 Material **Ib**

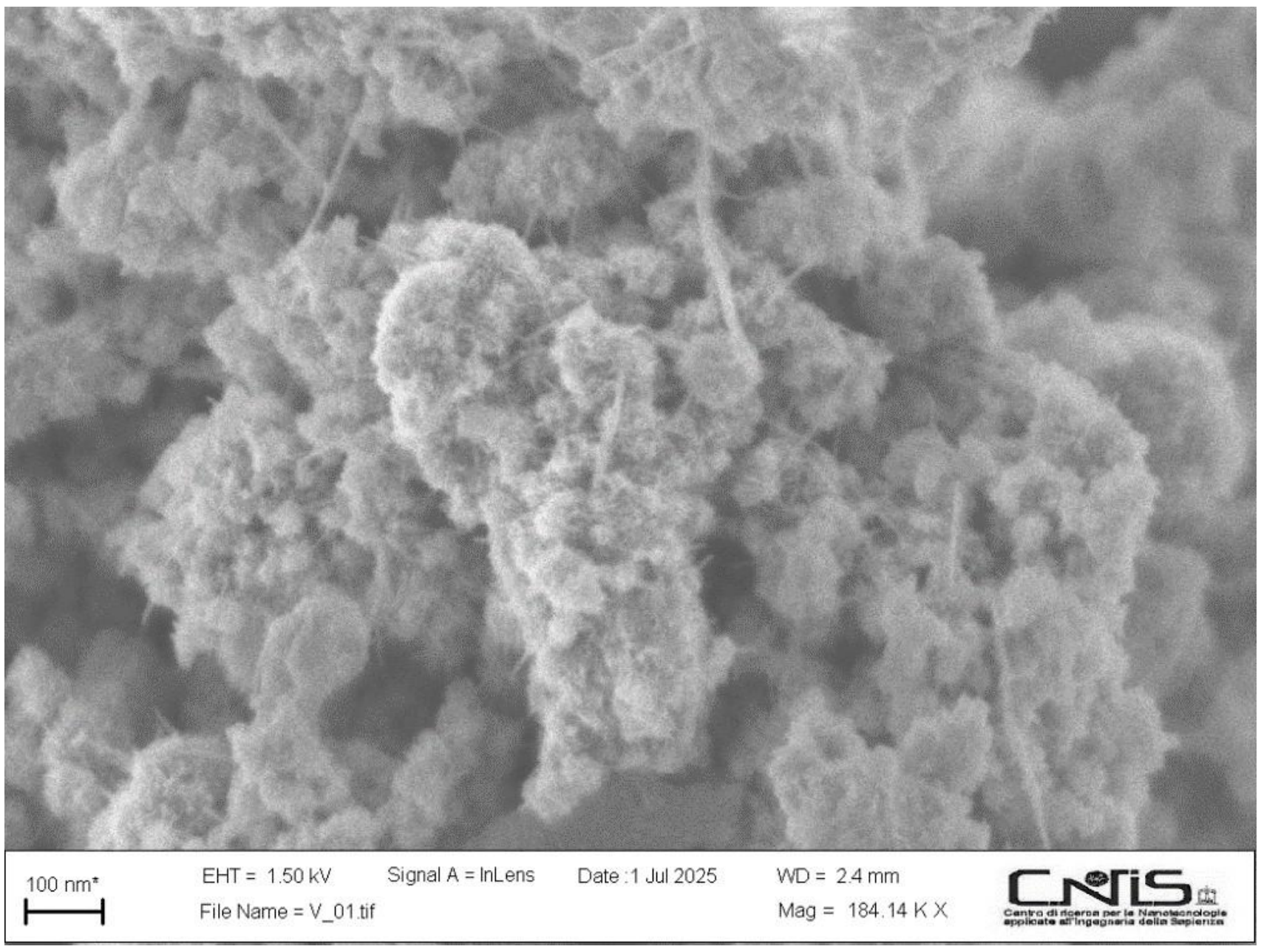

Figure S2.13 Material **II**

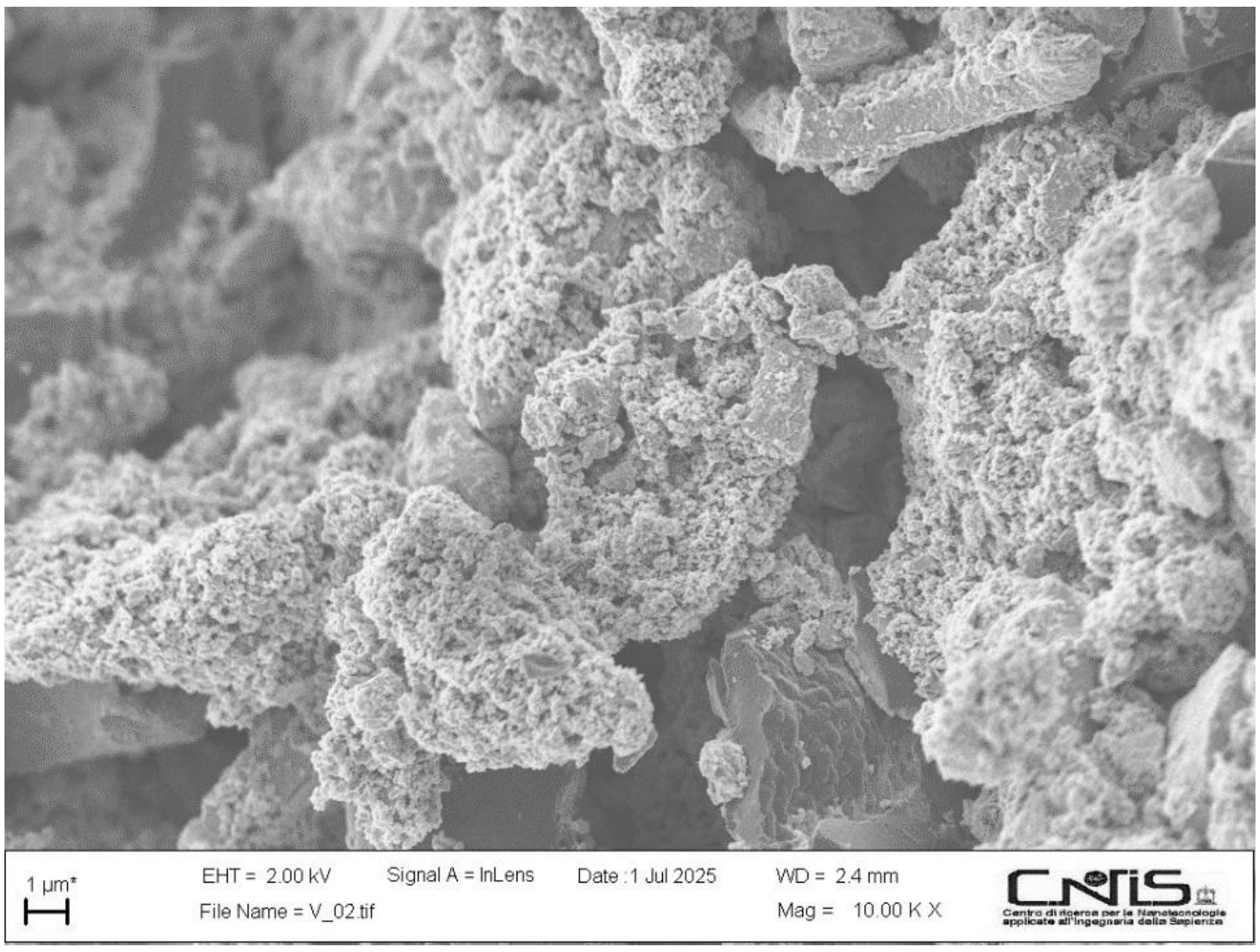

Figure S2.14 Material **II**

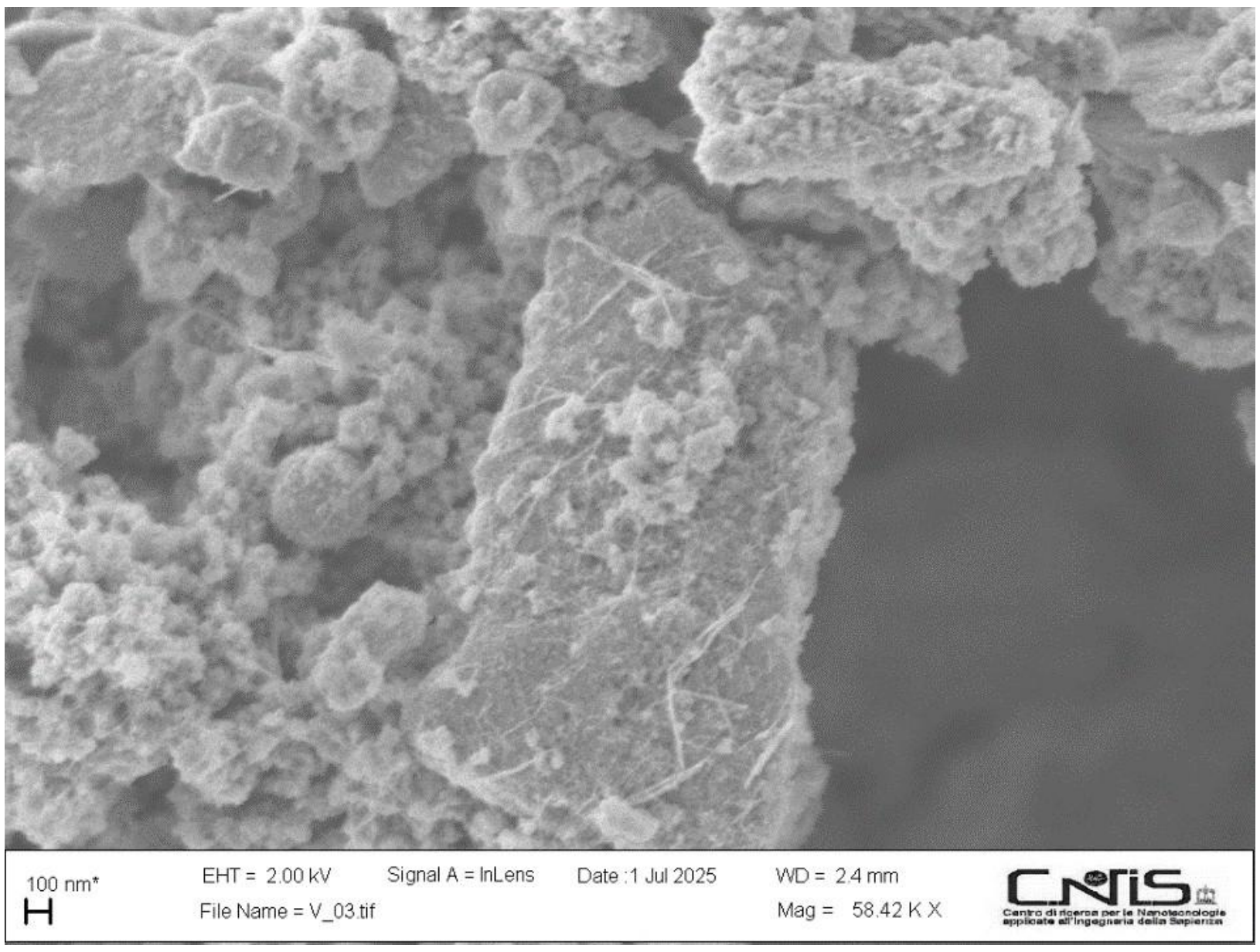

Figure S2.15 Material **II**

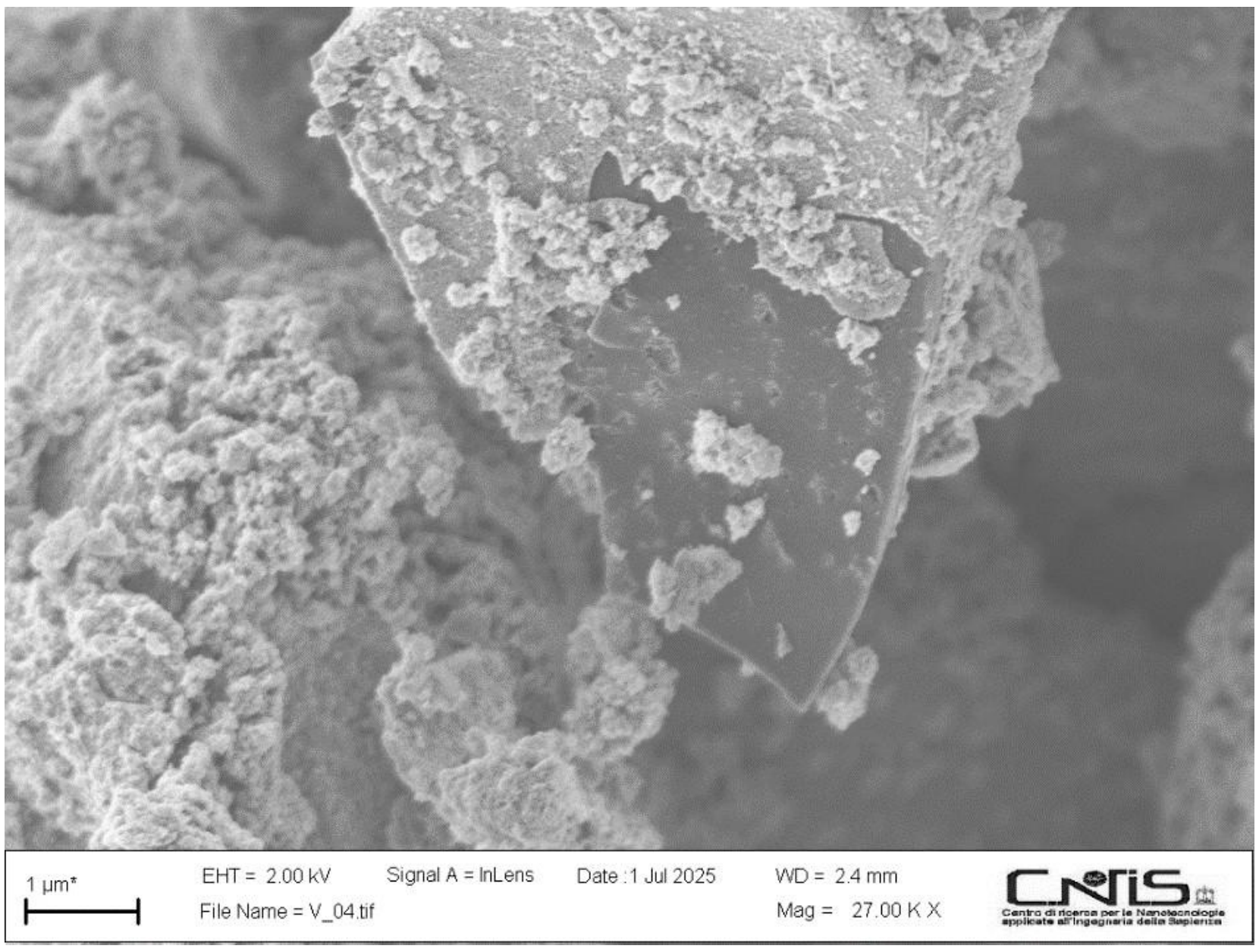

Figure S2.16 Material **II**

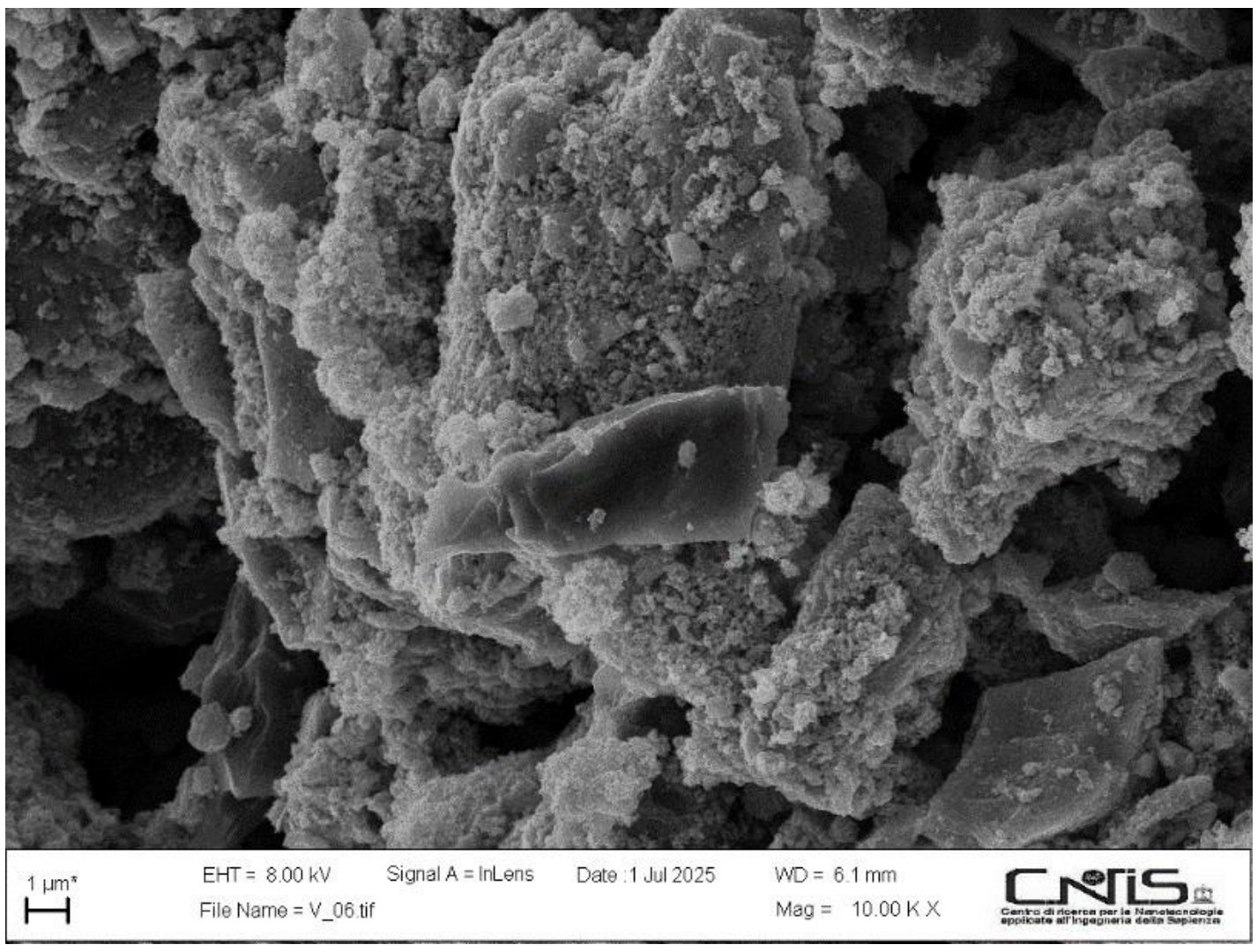

Figure S2.17 Material **II**

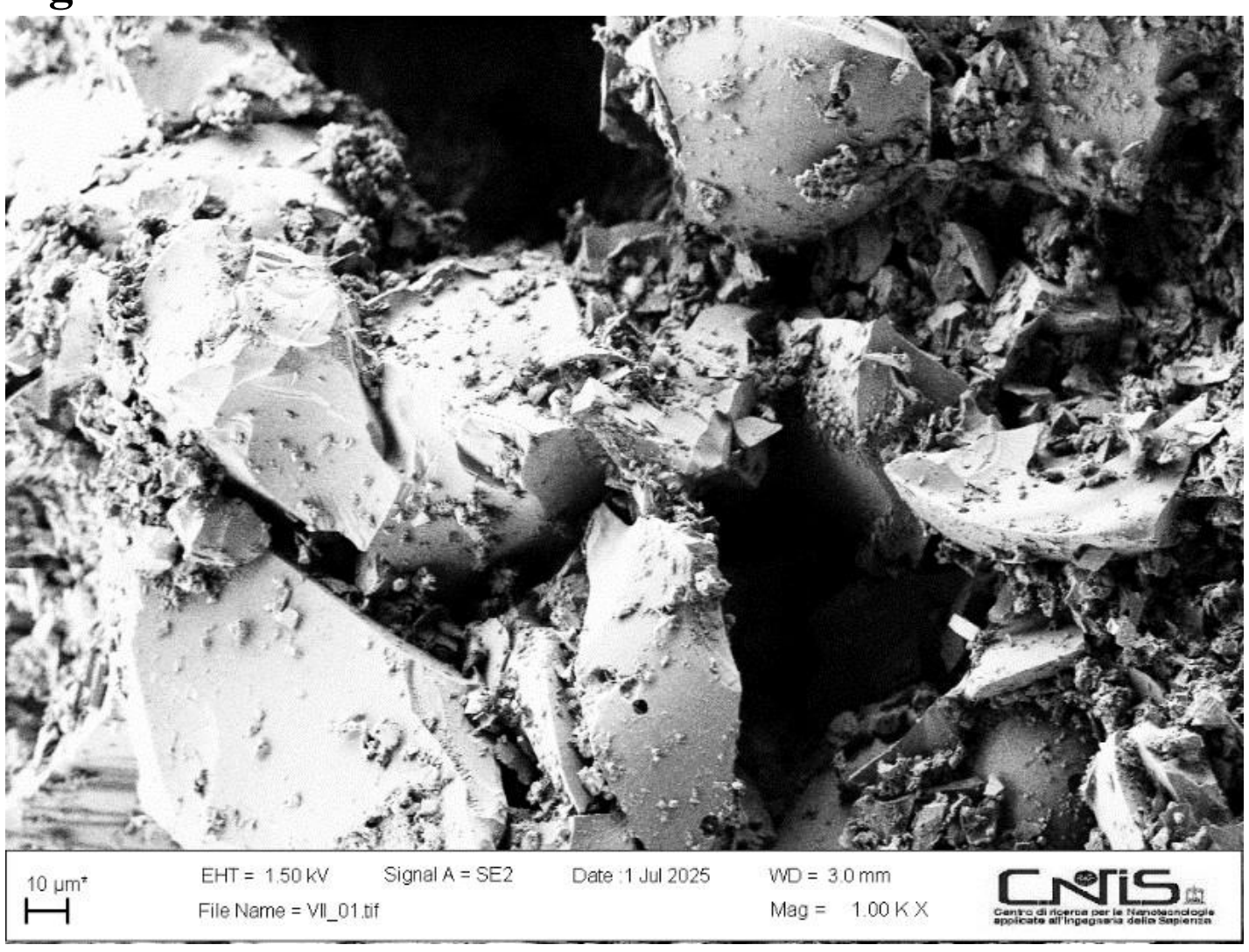

Figure S2.18 Material **IV**

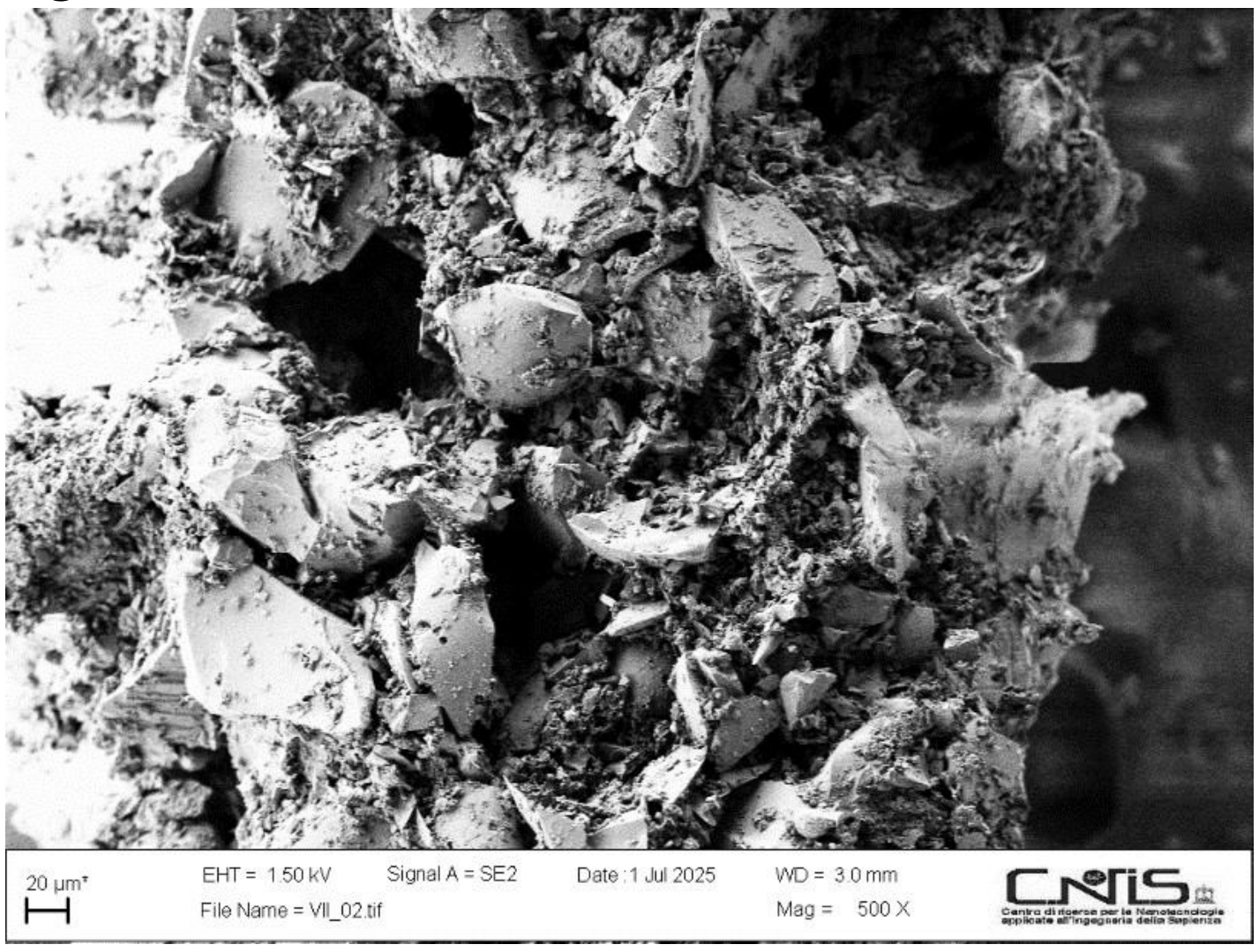

Figure S2.19 Material **IV**

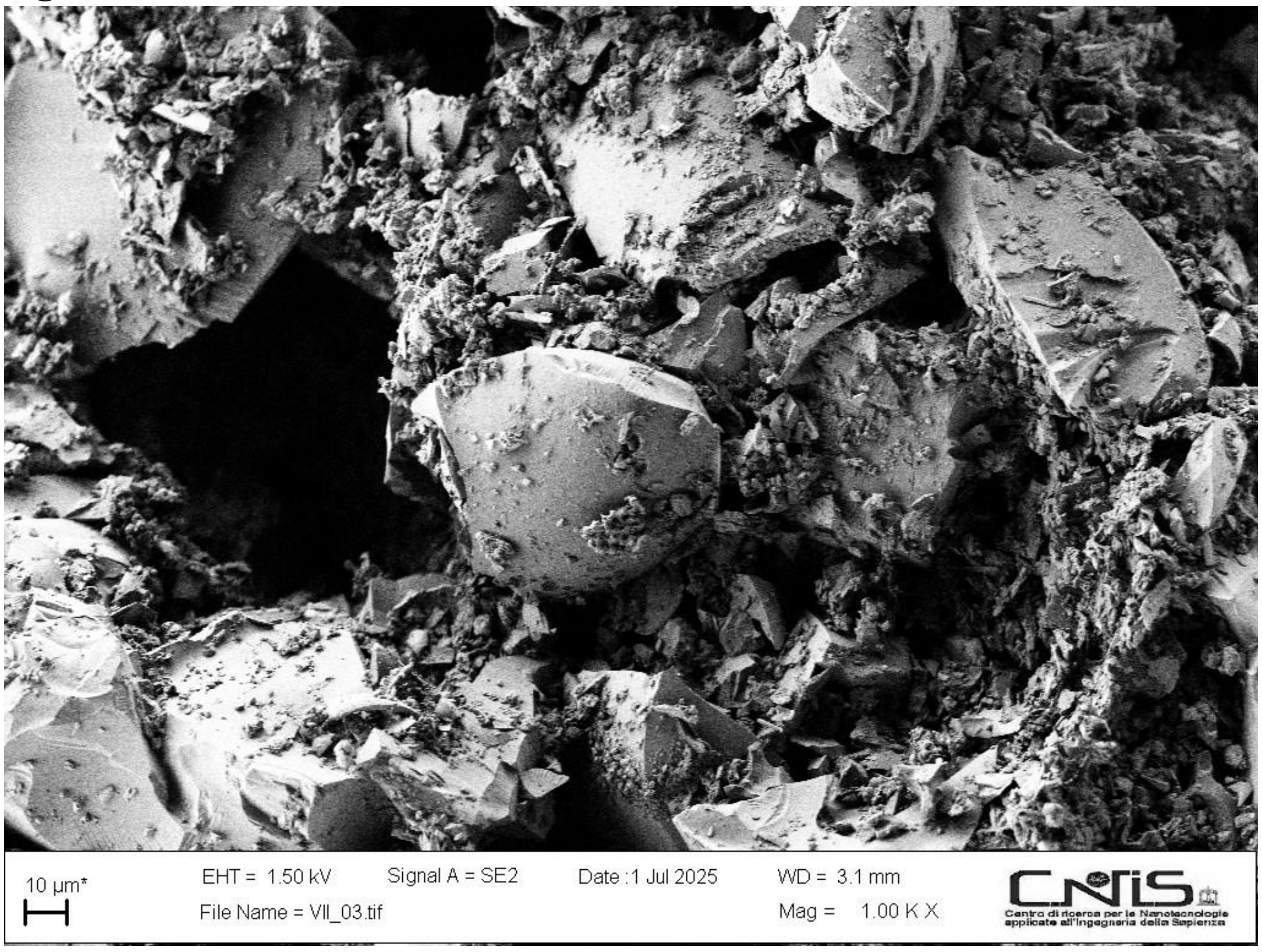

Figure S2.20 Material **IV**

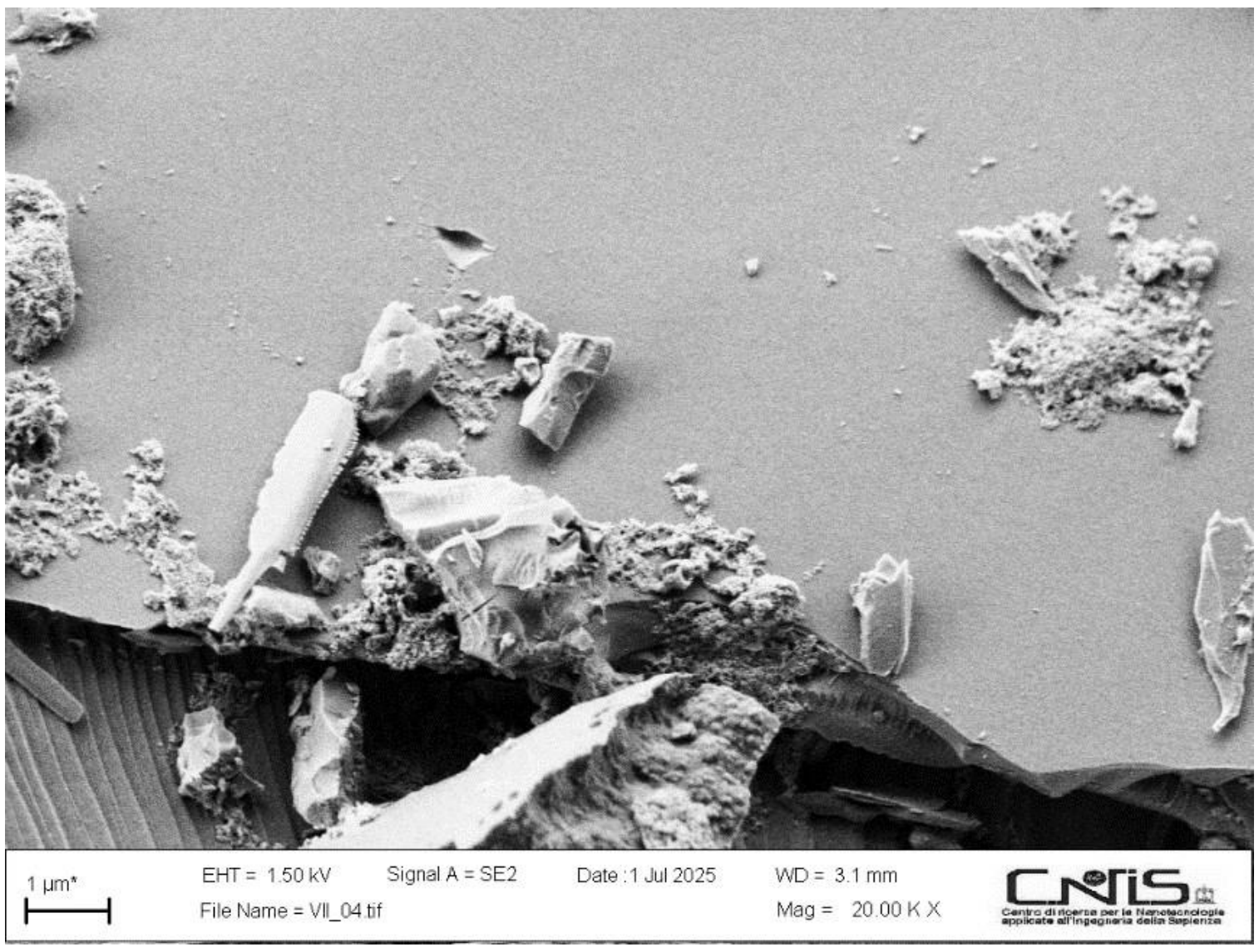

Figure S2.21 Material **IV**

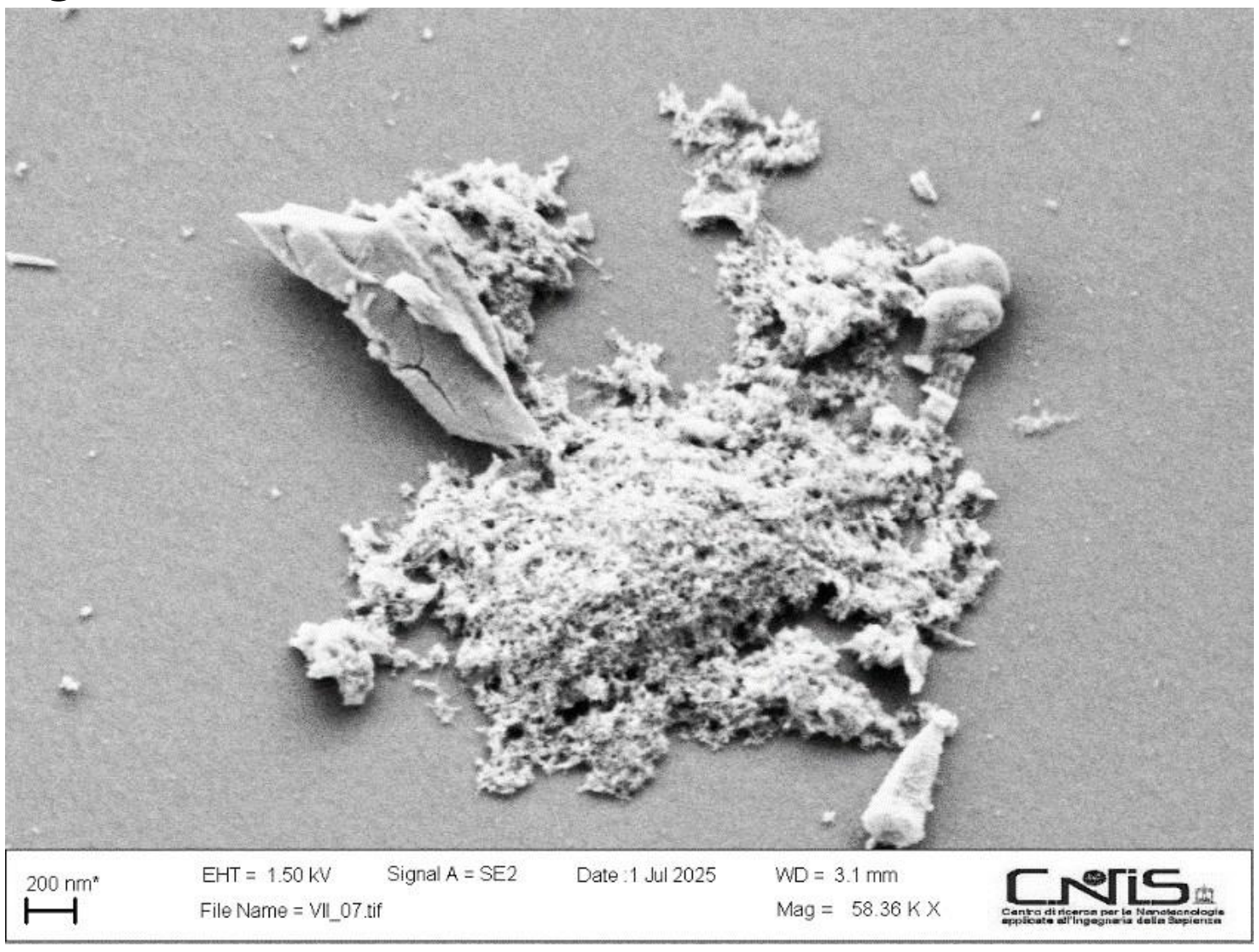

Figure S2.22 Material **IV**

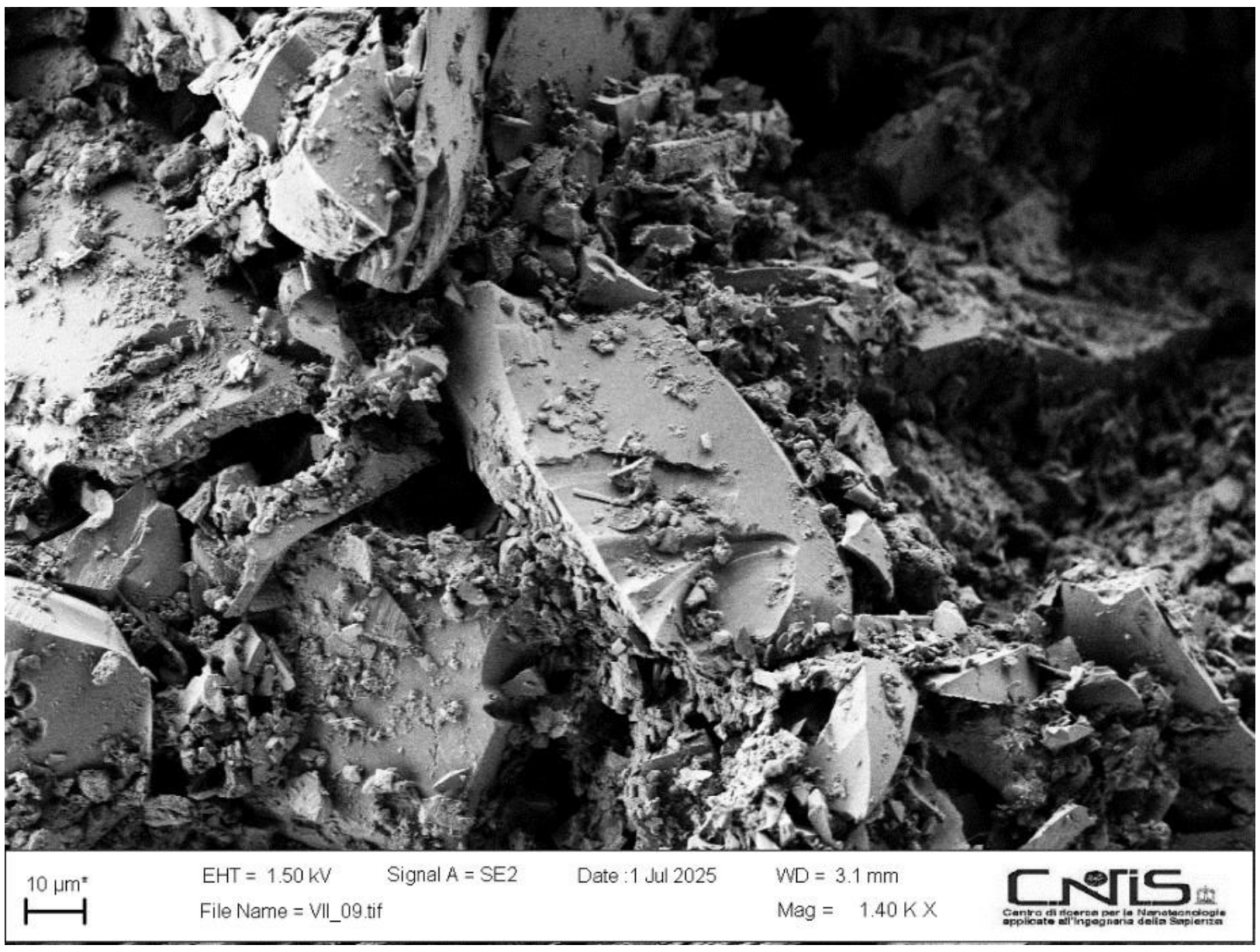

Figure S2.23 Material **IV**

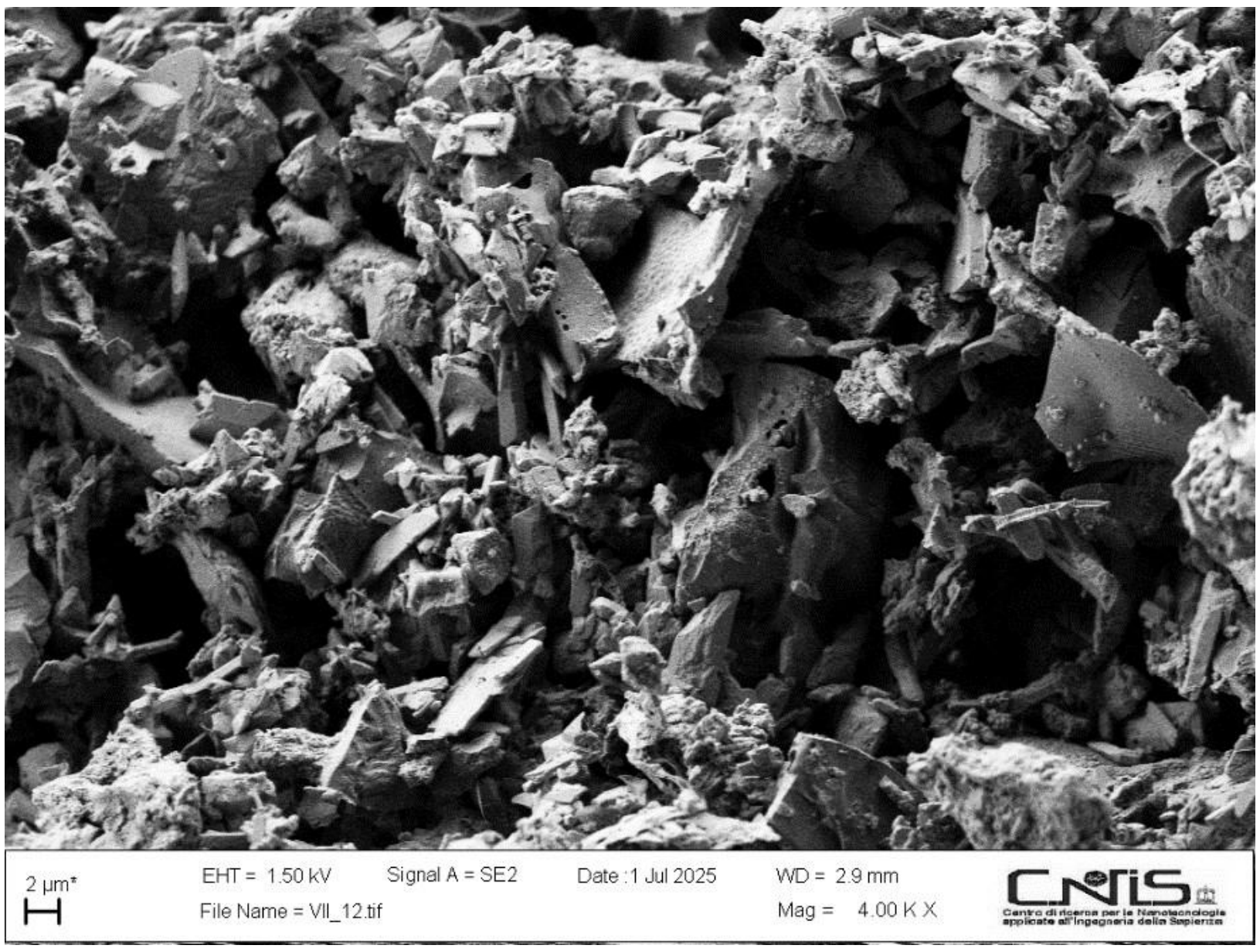

Figure S2.24 Material **IV**

S3 by energy-dispersive X-ray spectroscopy (EDX) experiments

In this section are listed the EDX reports for the indicated materials

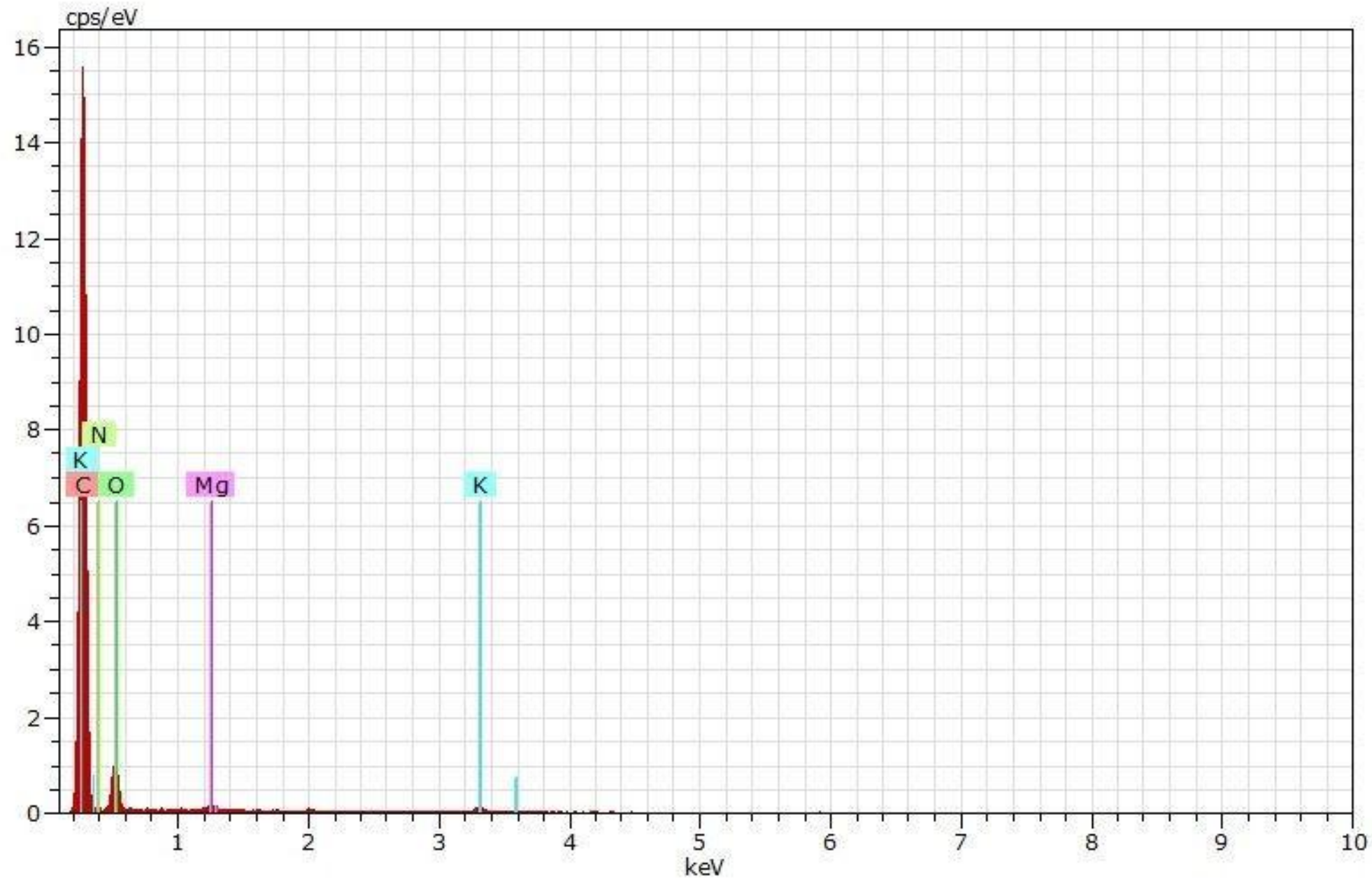

Spectrum: Biochar_ 6.xls

Element	unn. C [wt.%]	norm. C [wt.%]	Atom. C [at.%]	Error (3 Sigma) [wt.%]
Carbon	79.21	79.21	84.36	28.25
Oxygen	13.67	13.67	10.93	6.86
Potassium	2.68	2.68	0.88	0.55
Magnesium	0.57	0.57	0.30	0.22
Nitrogen	3.86	3.86	3.53	3.48
Total:	100.00	100.00	100.00	

Figure S3.1 Biochar SCG550

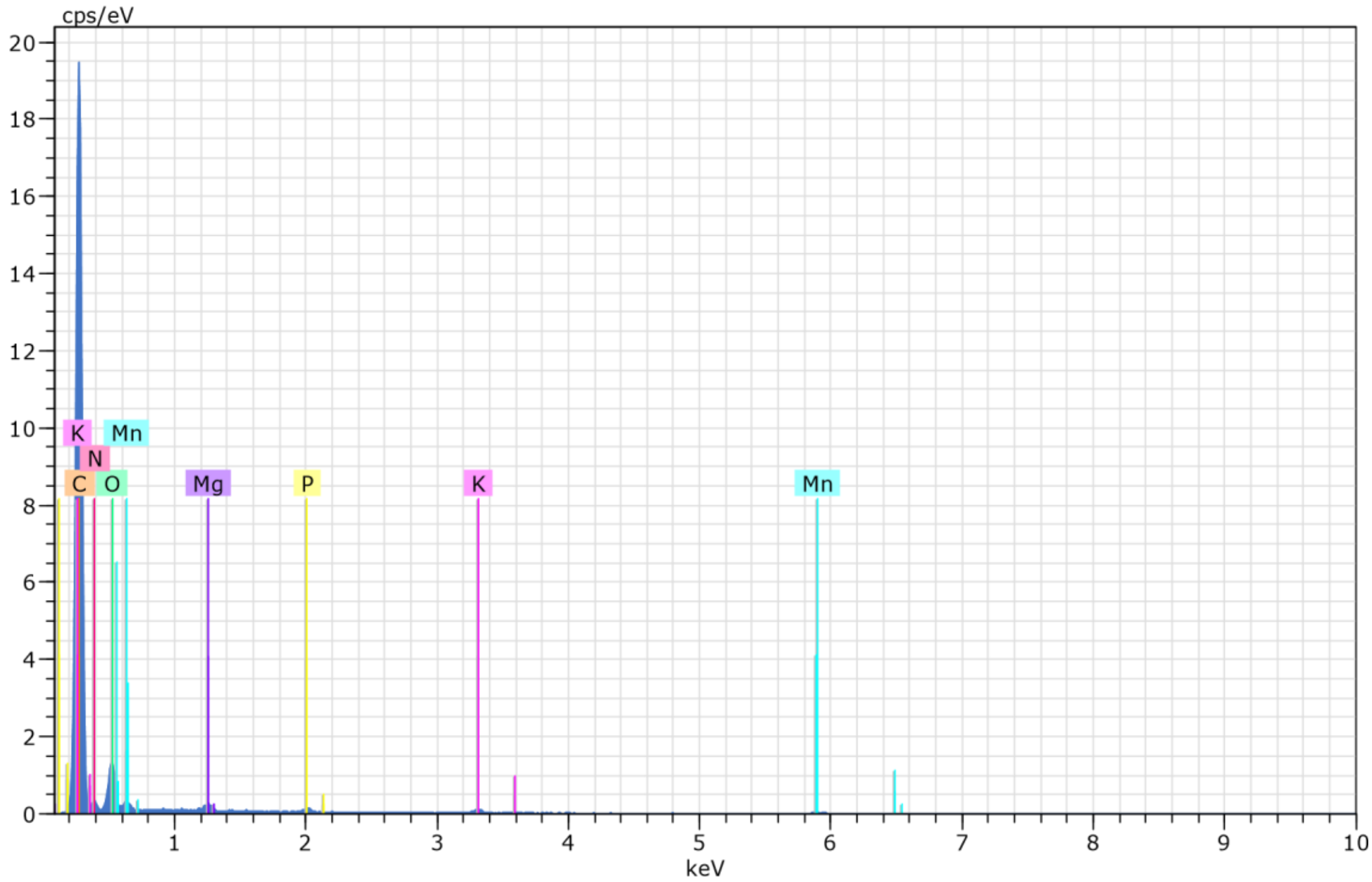

Spectrum: IV_ 3.xls

Element	unn. C [wt.%]	norm. C [wt.%]	Atom. C [at.%]	Error (3 Sigma) [wt.%]
Carbon	74.48	74.48	82.75	26.16
Oxygen	10.88	10.88	9.08	5.34
Magnesium	0.56	0.56	0.31	0.20
Nitrogen	5.91	5.91	5.63	4.11
Manganese	6.16	6.16	1.50	3.98
Potassium	1.36	1.36	0.47	0.34
Phosphorus	0.65	0.65	0.28	0.21
Total:	100.00	100.00	100.00	

Figure S3.2 Material **Ib**

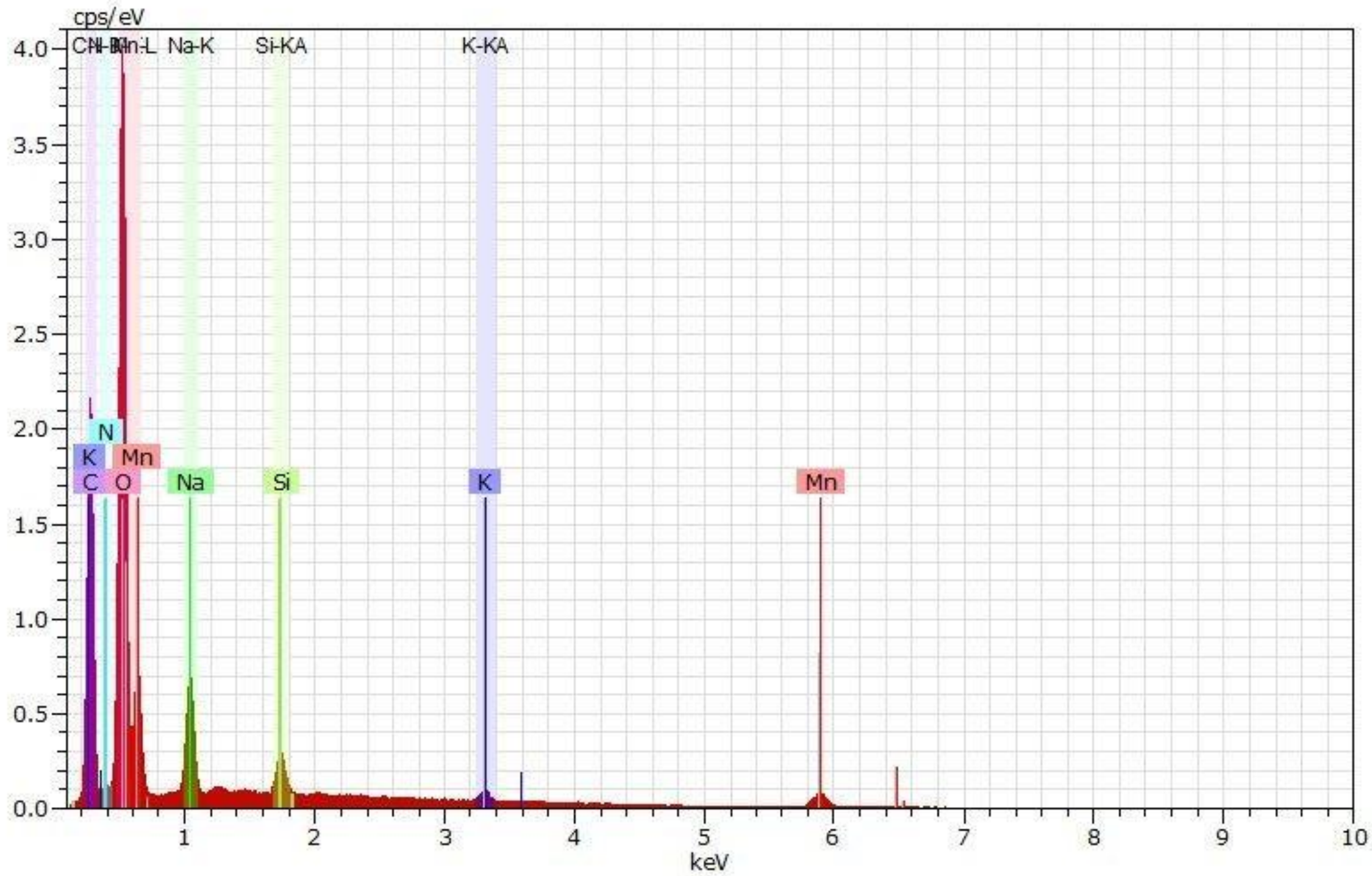

Spectrum: V_ 1.xls

Element	unn. C [wt.%]	norm. C [wt.%]	Atom. C [at.%]	Error (3 Sigma) [wt.%]
Carbon	66.67	66.67	72.44	24.39
Oxygen	20.10	20.10	16.39	9.37
Nitrogen	10.03	10.03	9.35	6.48
Sodium	3.20	3.20	1.82	0.71
Total:	100.00	100.00	100.00	

Figure S3.3 Material **II**

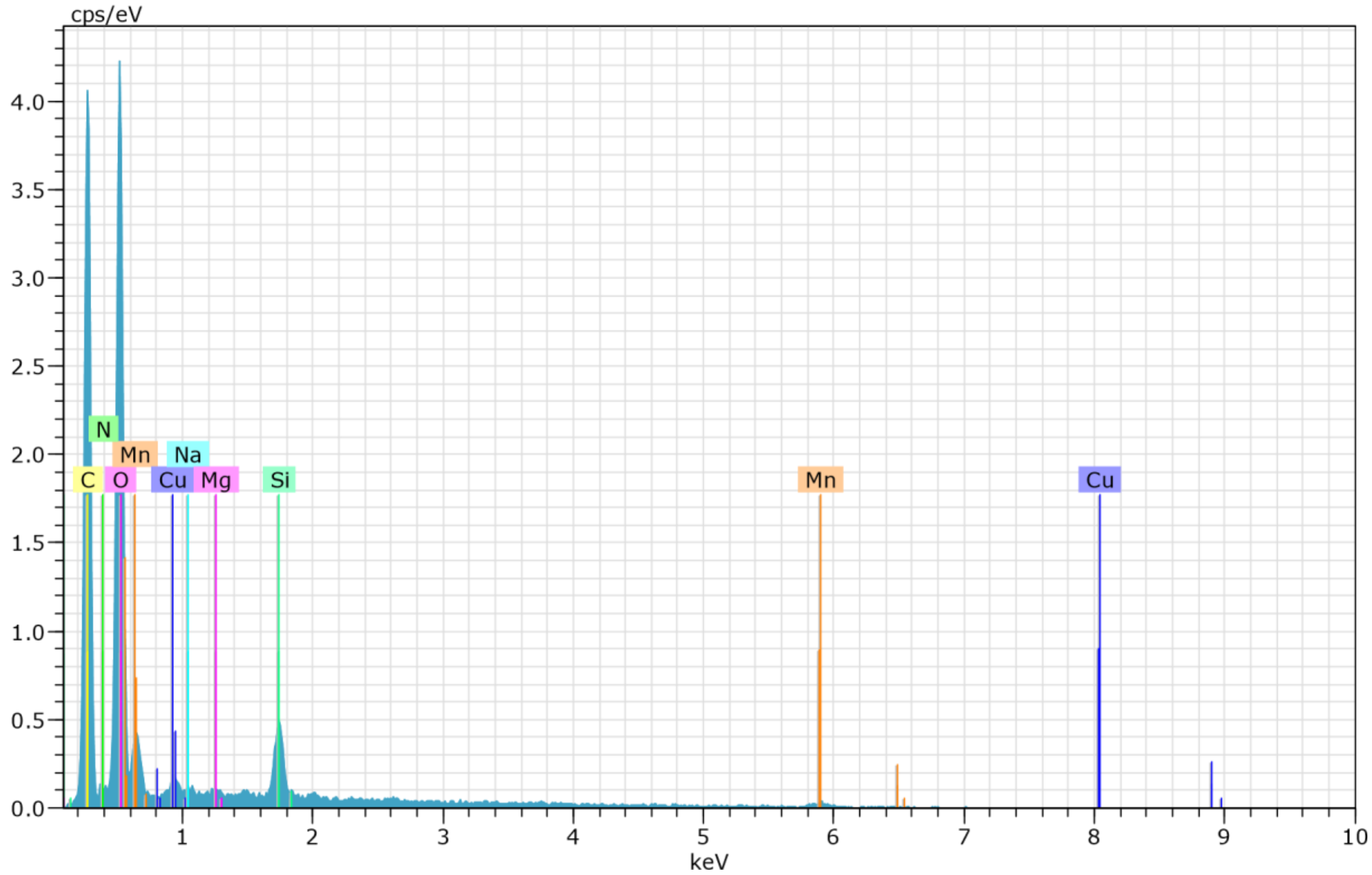

Spectrum: VII_ 1

Element	unn. C [wt.%]	norm. C [wt.%]	Atom. C [at.%]	Error (3 Sigma) [wt.%]
Oxygen	40.85	40.85	39.40	16.47
Carbon	36.05	36.05	46.32	14.73
Silicon	3.92	3.92	2.15	0.67
Manganese	8.88	8.88	2.49	9.03
Nitrogen	8.20	8.20	9.03	1.87
Copper	1.87	1.87	0.45	1.66
Sodium	0.17	0.17	0.11	0.14
Magnesium	0.06	0.06	0.04	0.11
Total:	100.00	100.00	100.00	

Figure S3.4 Material **IV**

S4 MicroRaman Investigation

In this section are reported the images shot during the microRaman investigation through optical microscopy.

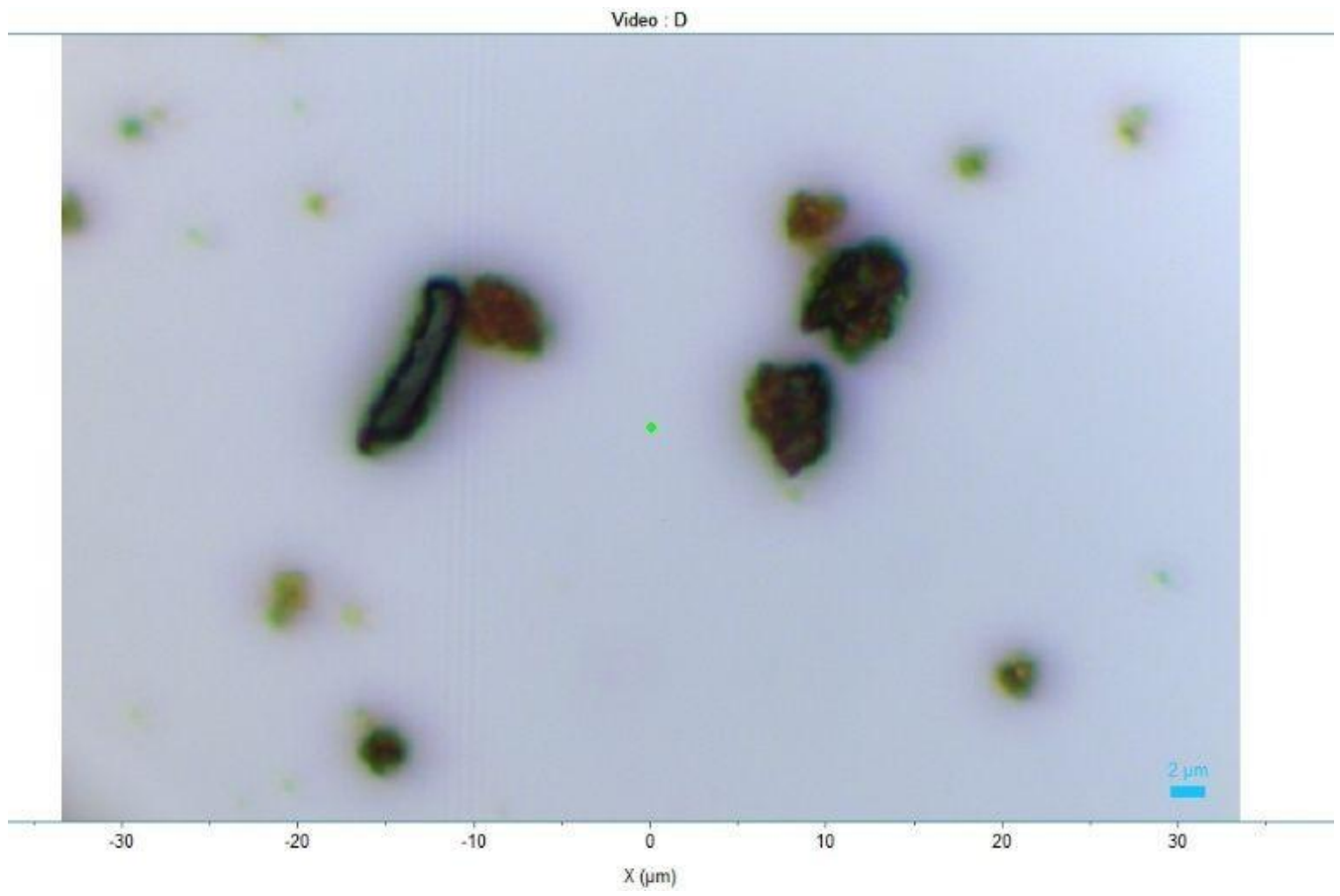

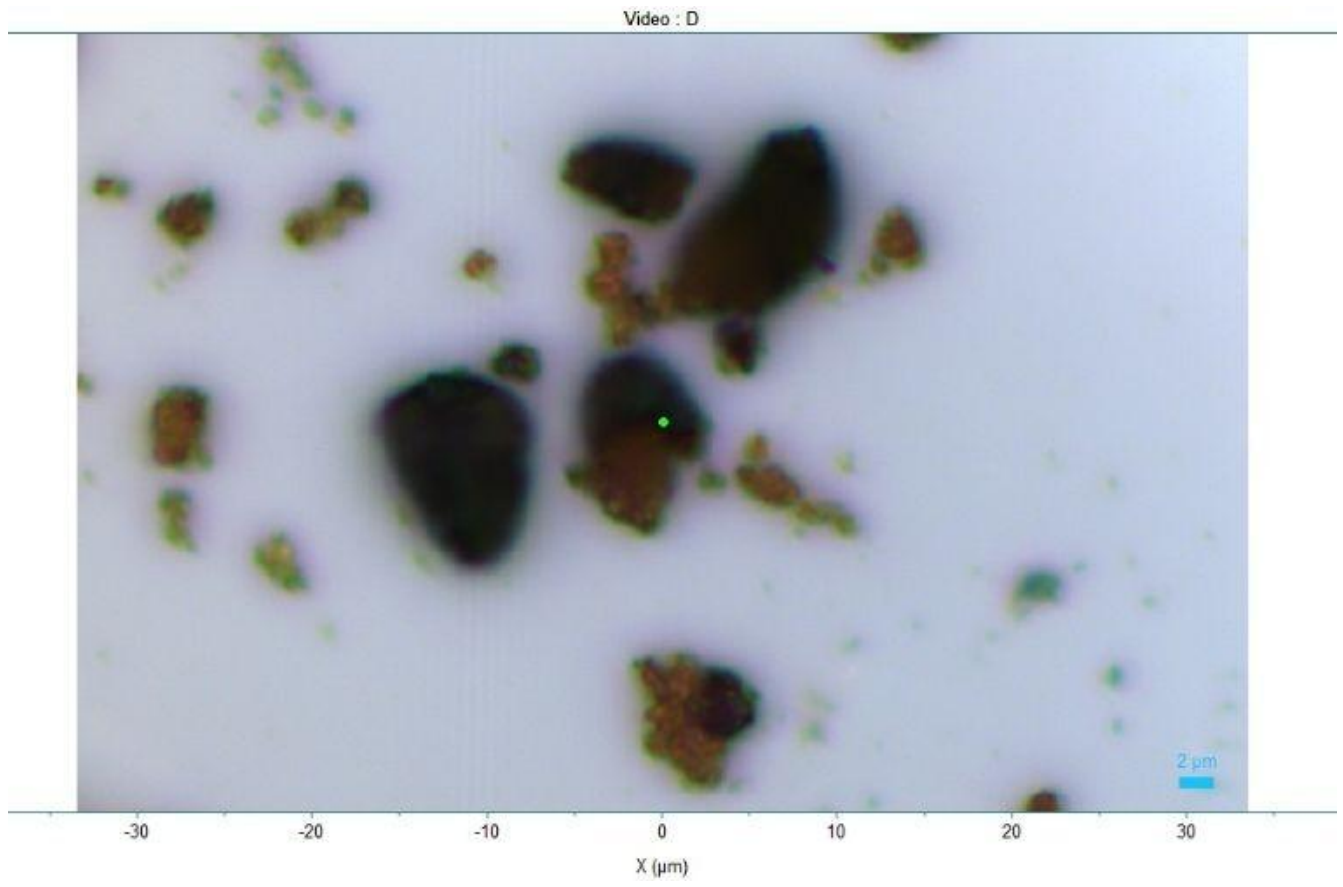

Figure S4.1 Material **Ib**

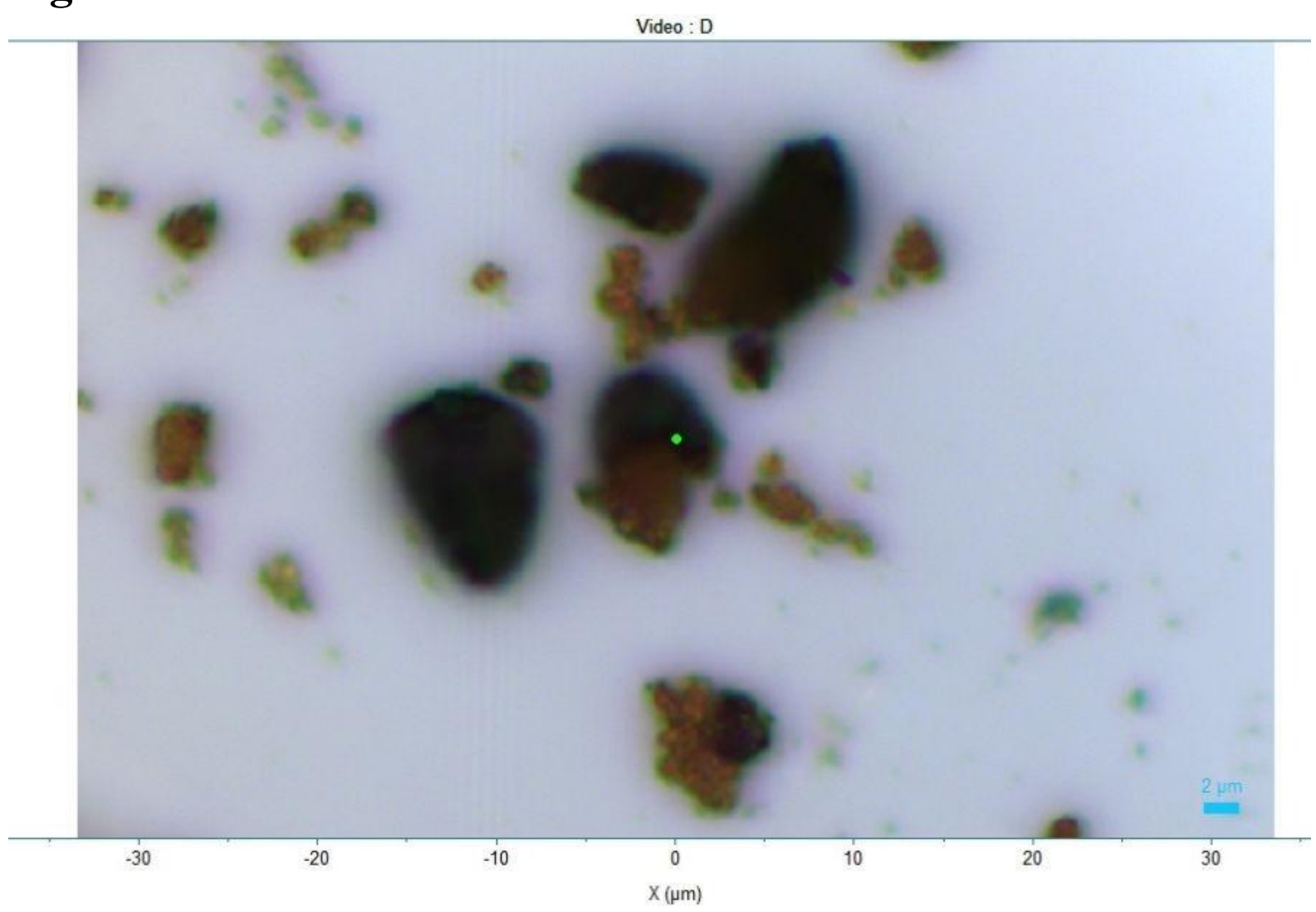

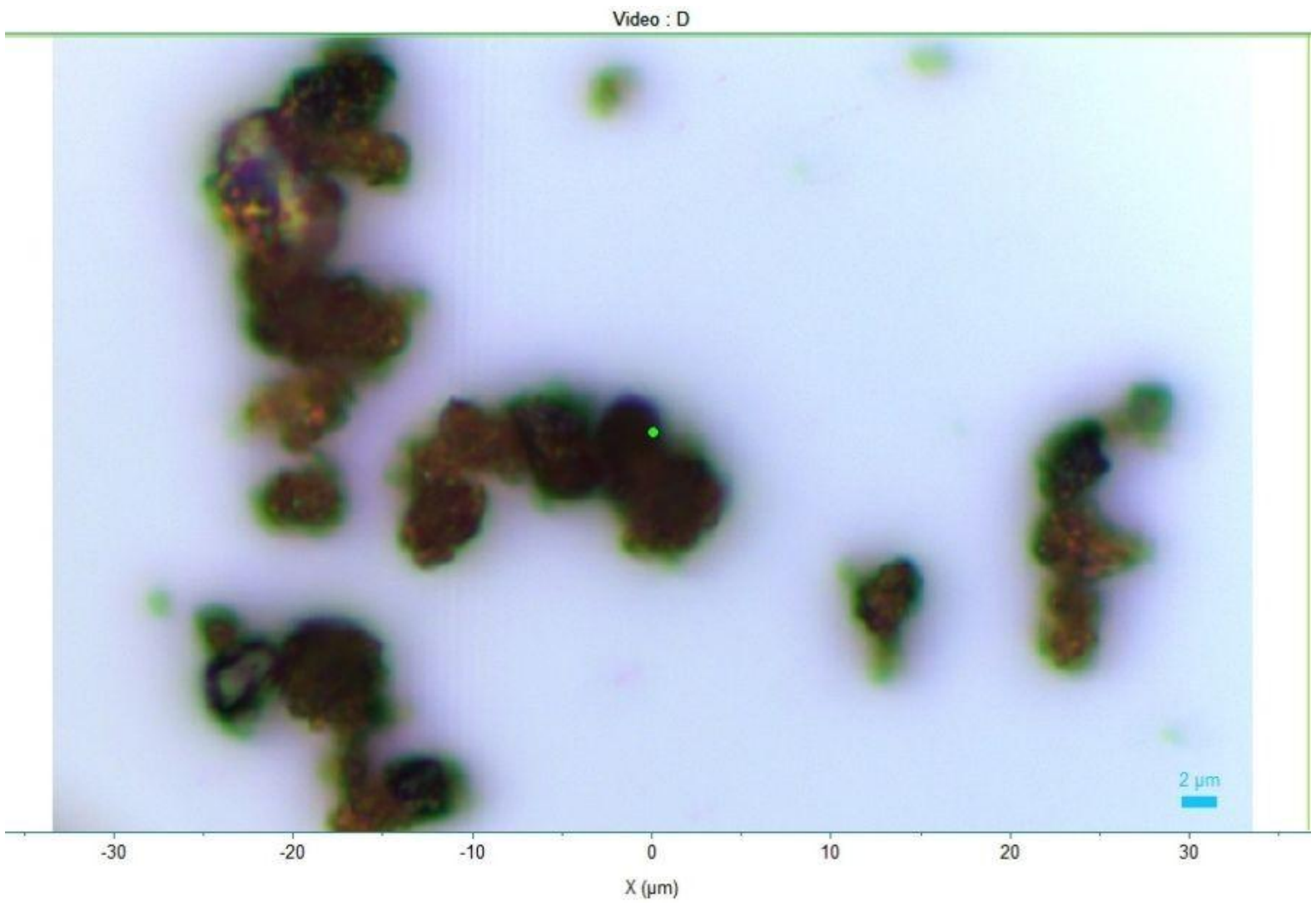

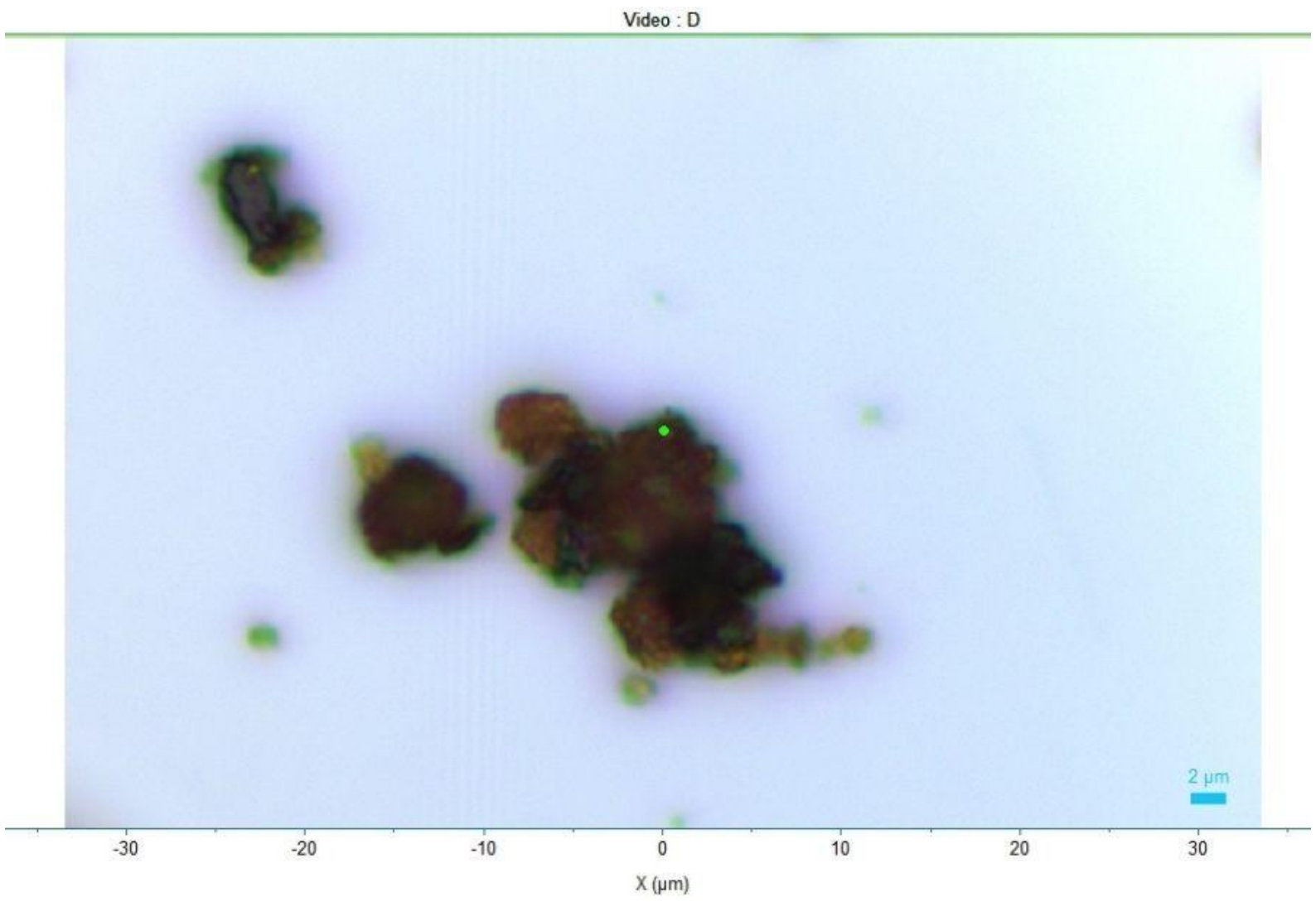

Figure S4.2 Material **II**

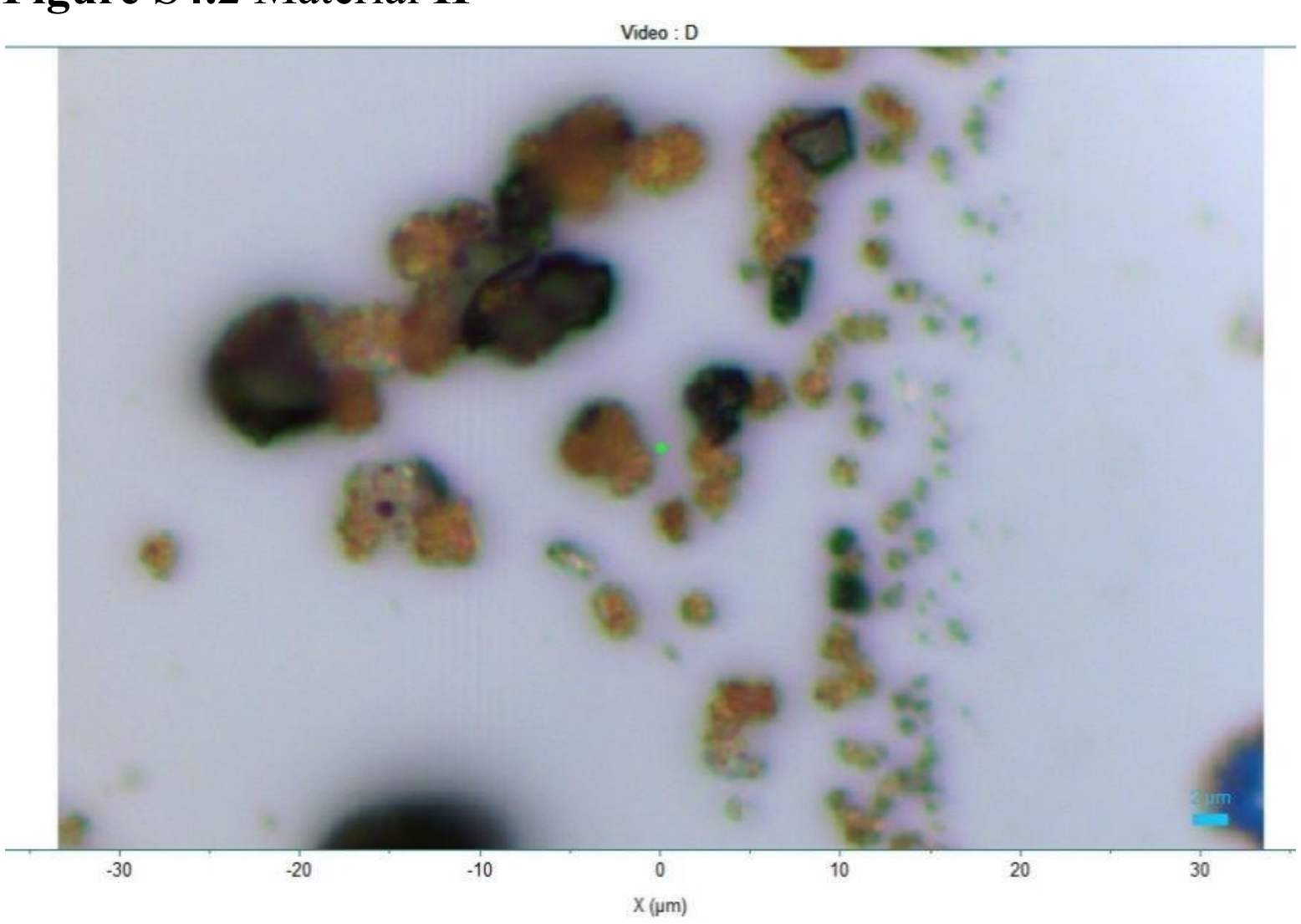

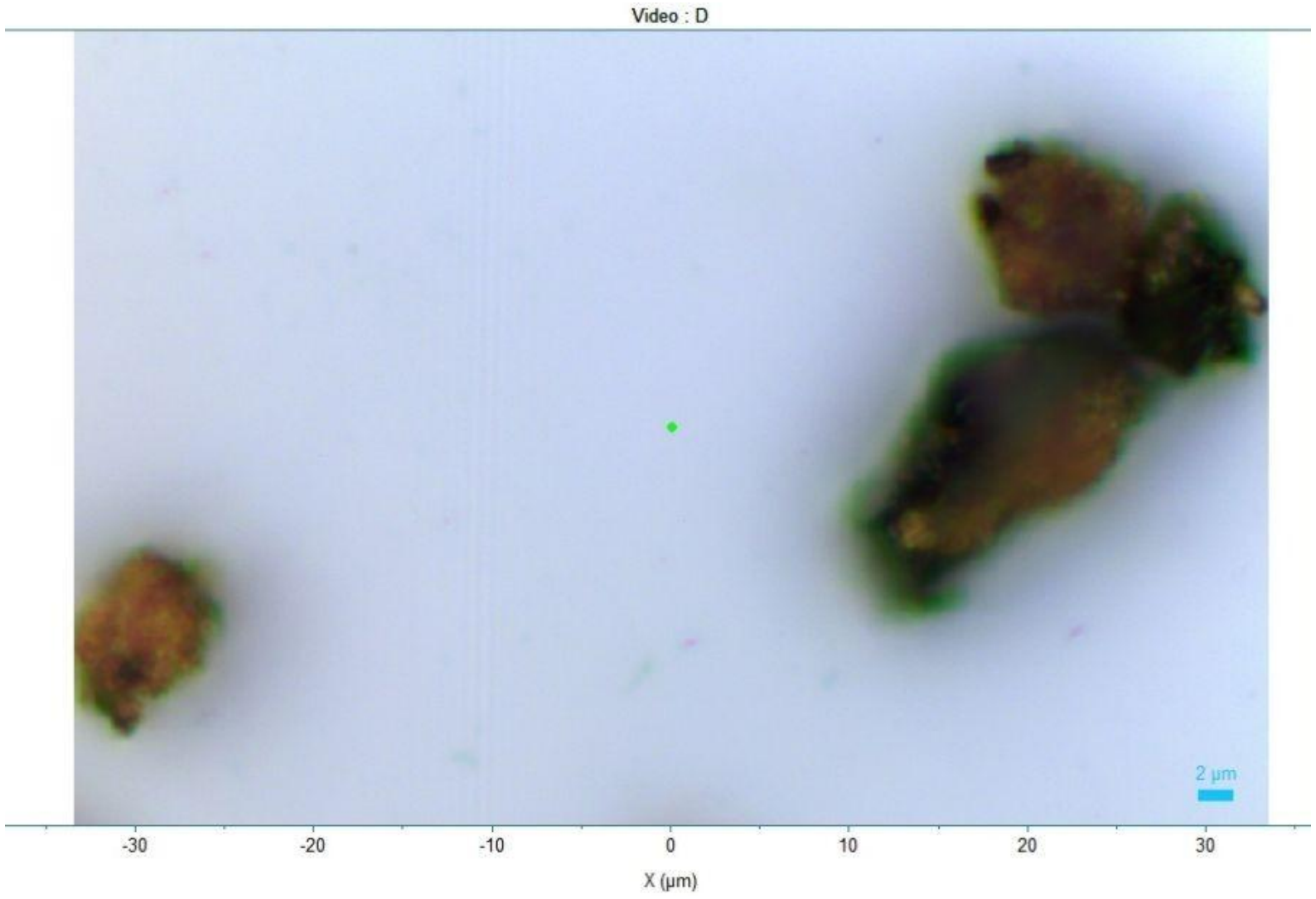

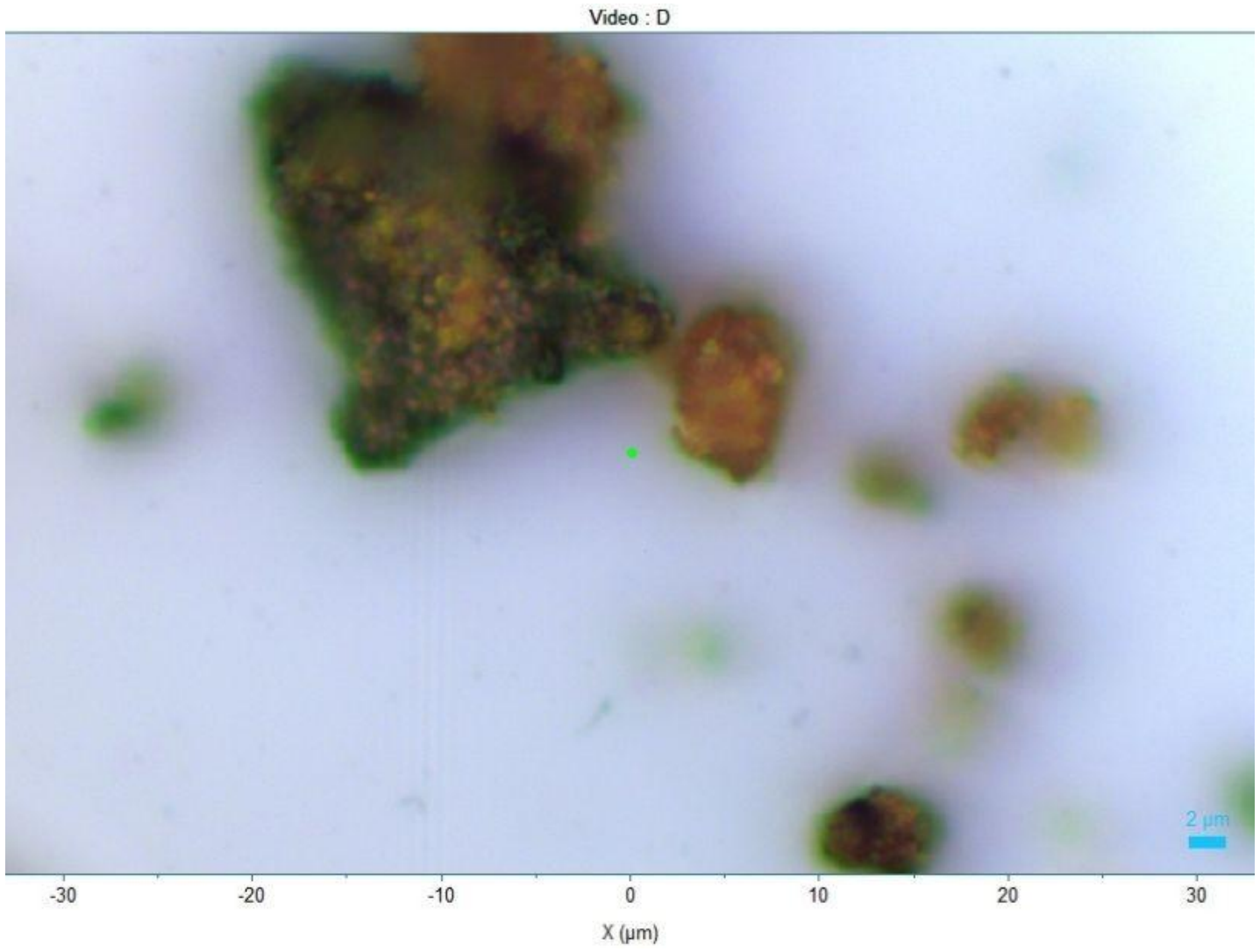

Figure S4.3 Material **III**

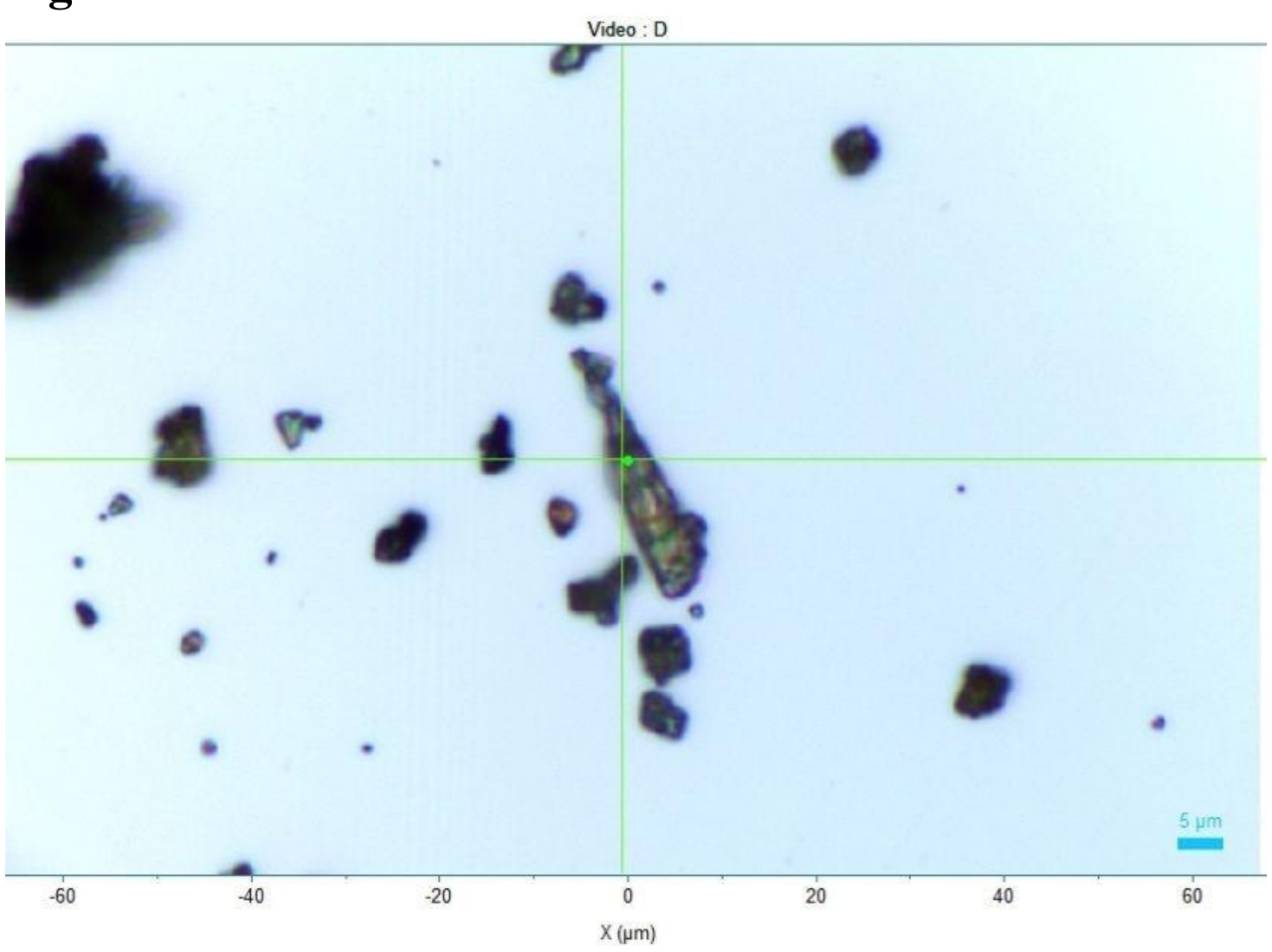

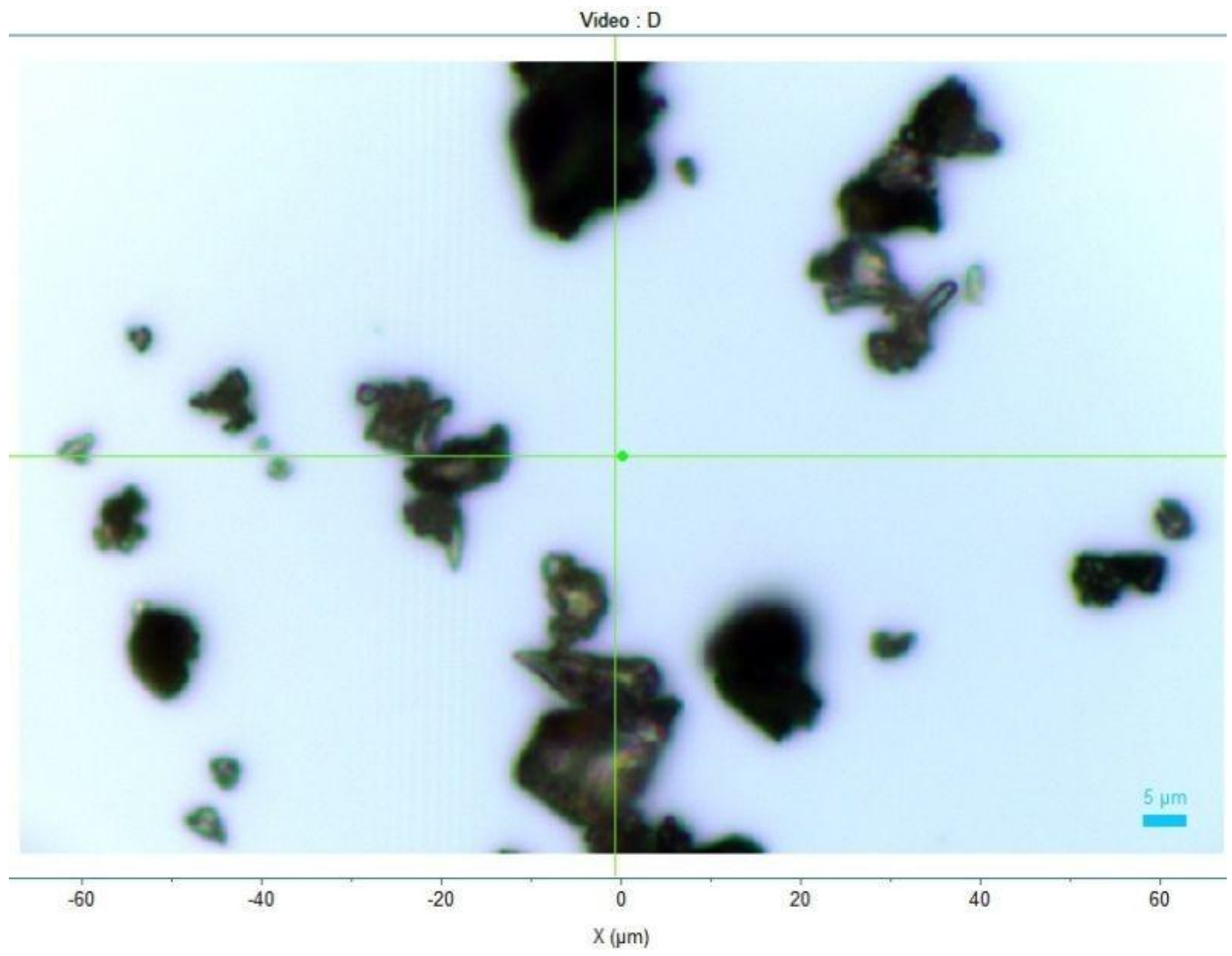
Video : D
5 µm
-60
-40
-20
0
20
40
60
X (µm)

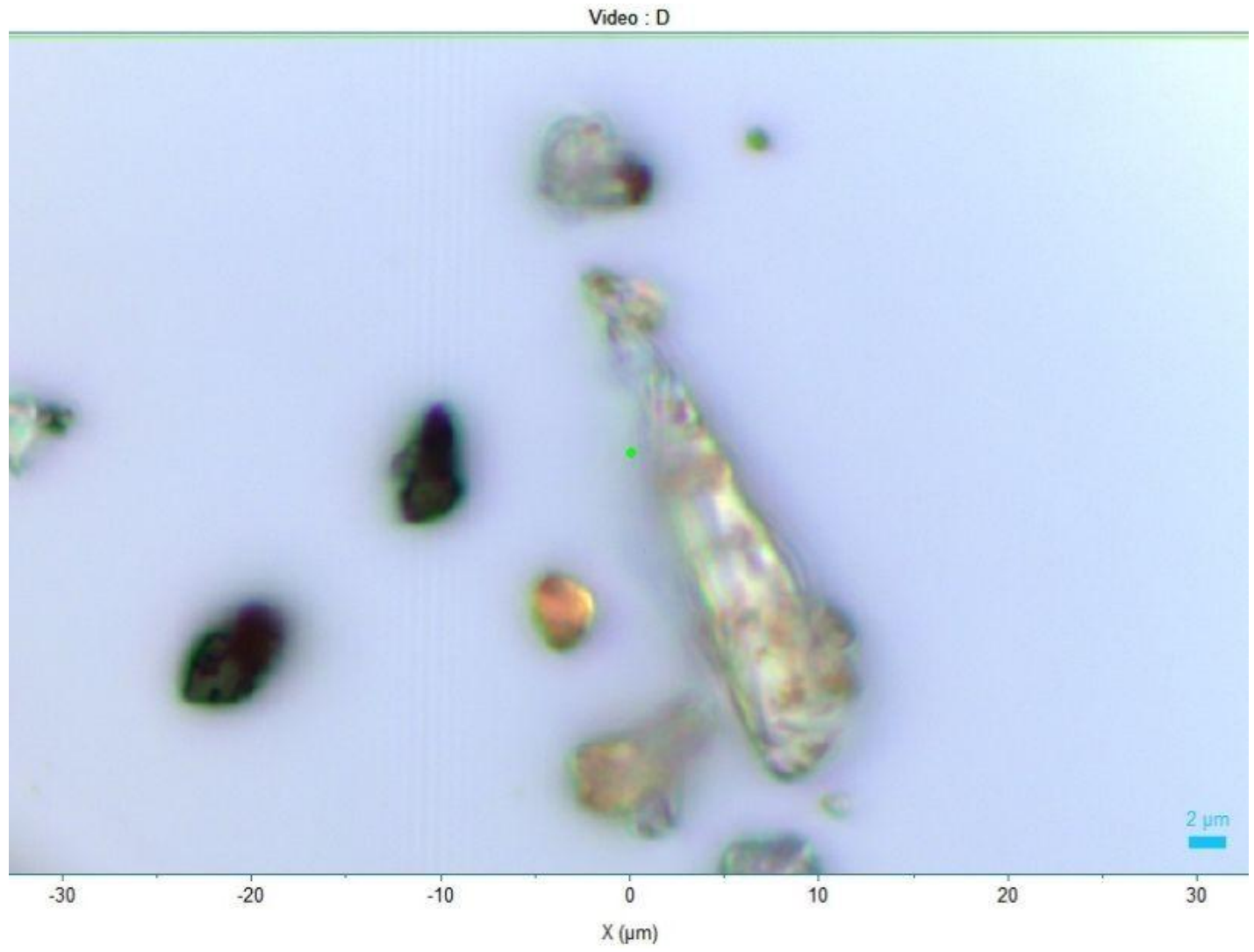
Video : D
2 µm
-30
-20
-10
0
10
20
30
X (µm)

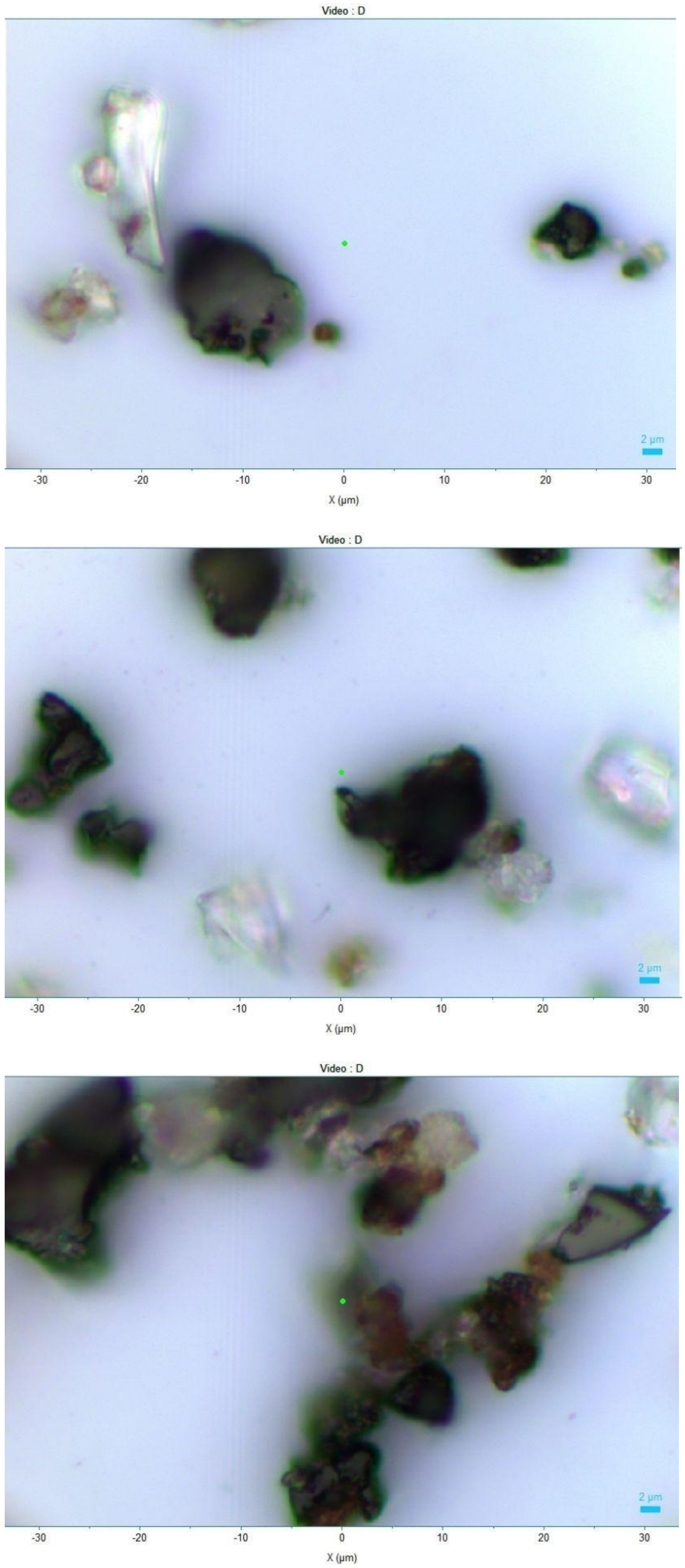

Figure S4.4 Material **IV**

S5 Electroanalytical Measurements

Randles Equivalent Circuit

Schematic representation of the Randles circuit used to fit EIS data and extract the charge transfer resistance (Rct). The model includes the solution resistance (Rs), the double-layer capacitance modeled as a constant phase element (CPE), the charge transfer resistance (Rct), and the Warburg diffusion element (W).

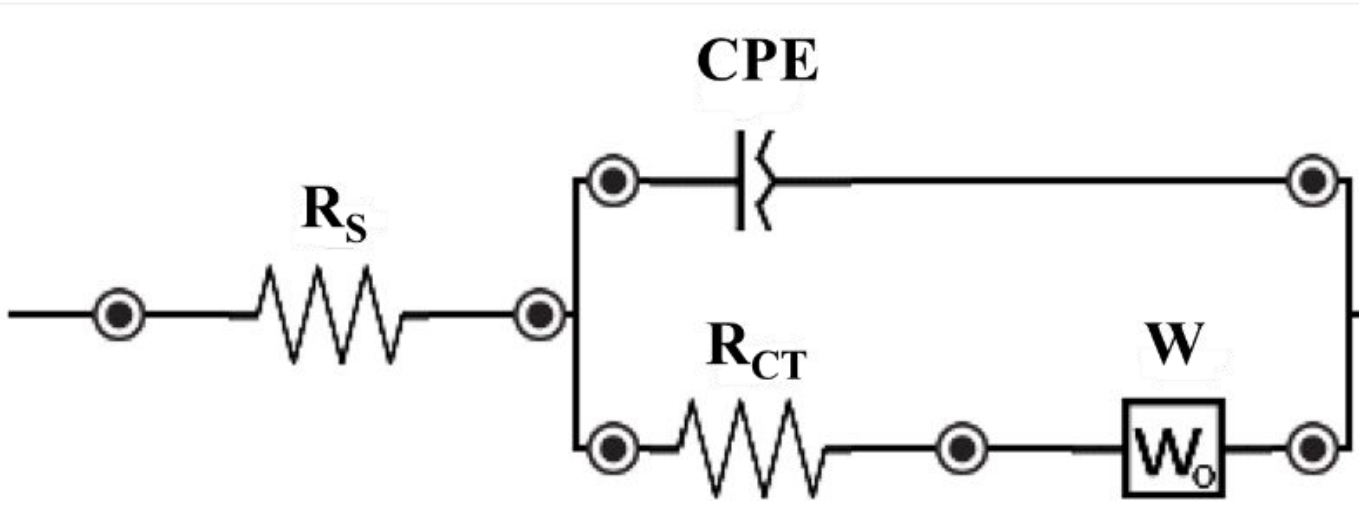

Figure S5.1 Randles Equivalent Circuit

Kramers–Kronig validity tests

Linear Kramers–Kronig validity tests were performed to verify the internal consistency of the EIS spectra used for Rct extraction. The analysis was carried out using the Linear Kramers–Kronig Validity Test software developed by KIT. Representative spectra recorded on Clicked Biochar electrodes after AP immobilization at increasing enzyme concentrations were analyzed. The residual plots showed low and randomly distributed real and imaginary residuals, generally within ±1%, confirming that the EIS datasets fulfilled the linearity, causality, and stability requirements needed for reliable impedance fitting.

a)

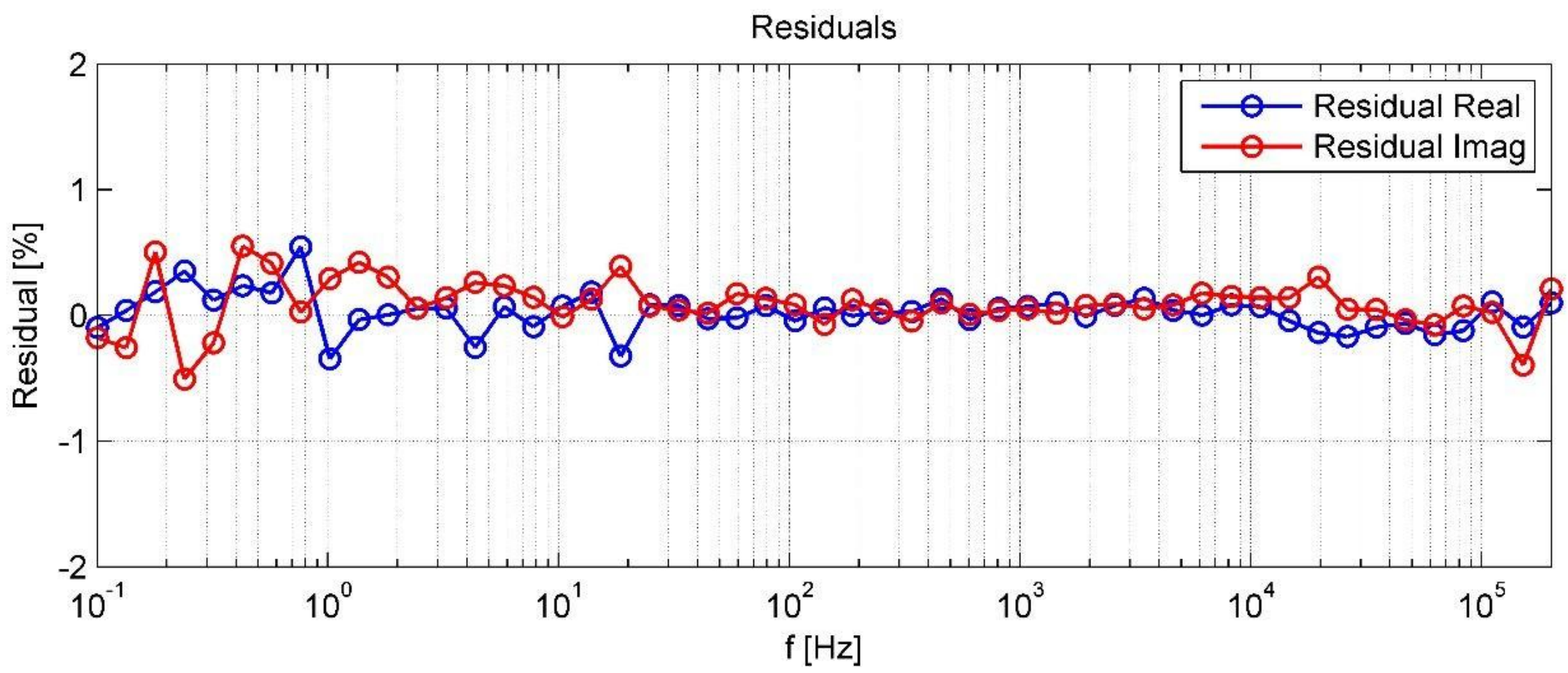

b)

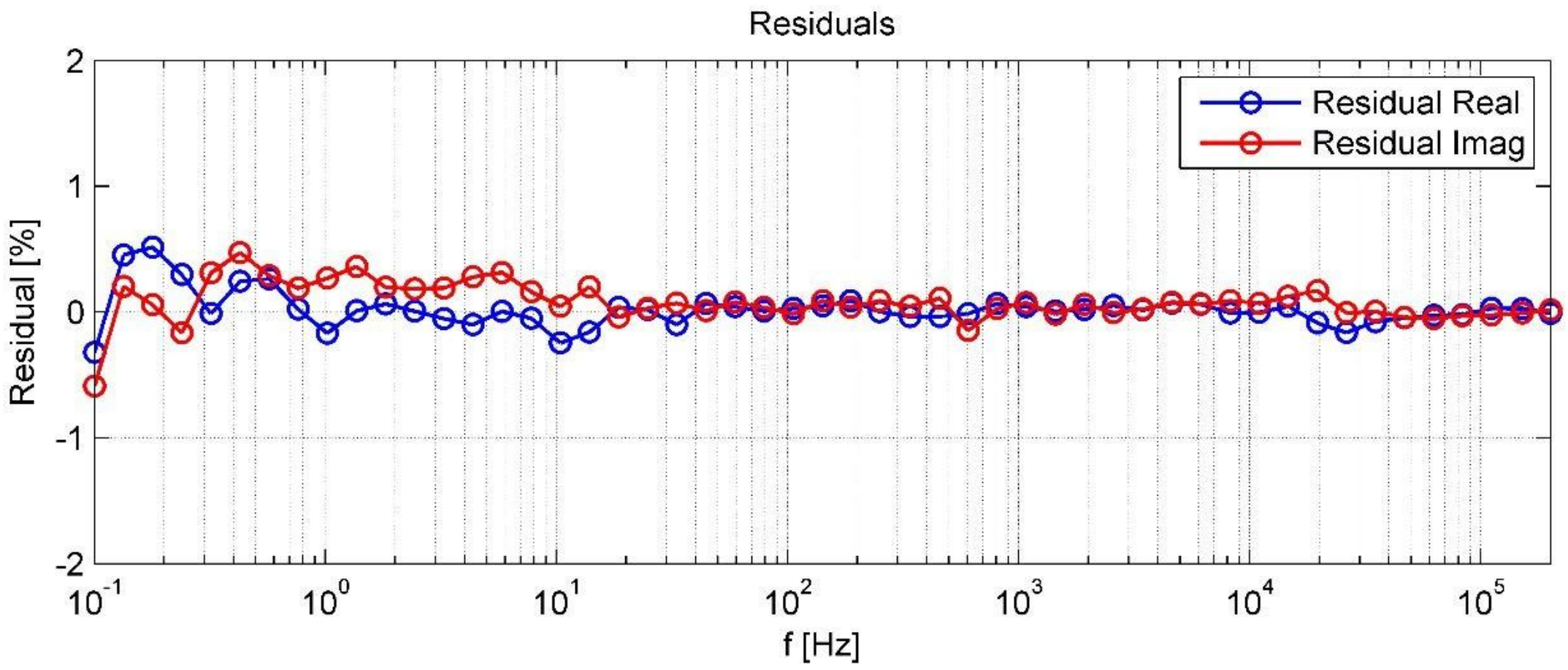

c)

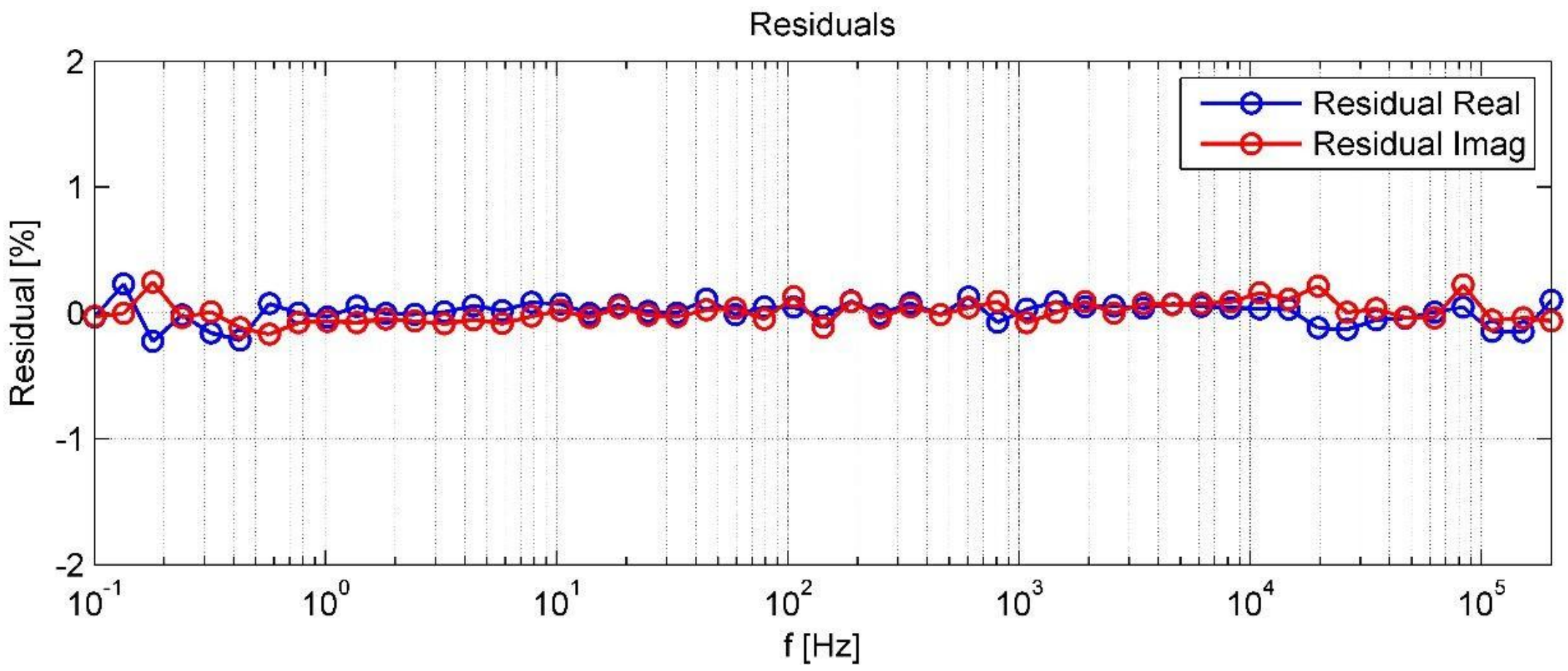

d)

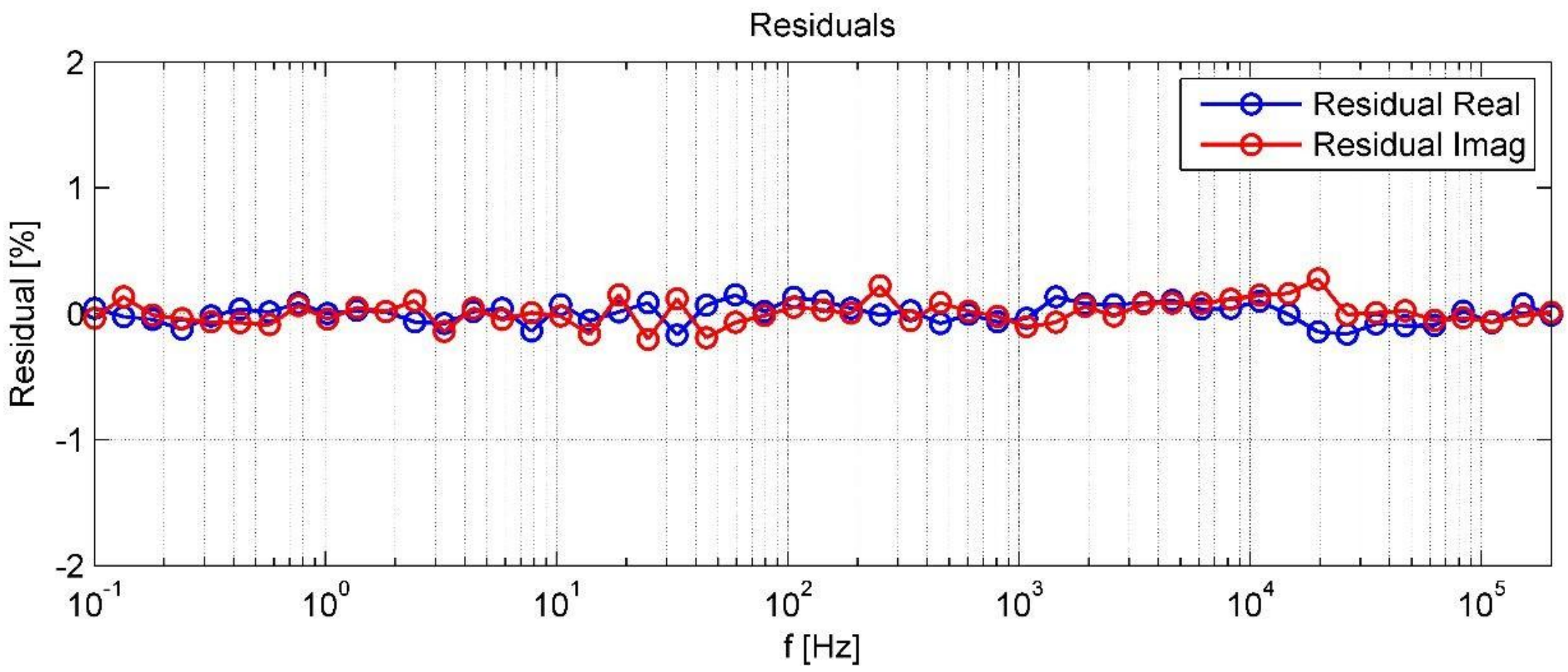

Figure S5.2. Linear Kramers–Kronig validity test of representative EIS spectra recorded on Clicked Biochar electrodes after AP immobilization at different enzyme concentrations: a) 0U/ml, b) 0.5U/ml, c) 1U/ml, d) 3U/ml. Residual plots for the real and imaginary components are reported.

BCA assay and EIS test

BCA protein quantification (Bicinchoninic Acid assay) was performed to provide an independent evaluation of AP retention on SCG550 and Clicked Biochar electrodes. The assay was carried out

following the manufacturer's instructions for µL of sample volume ,using the Sigma-Aldrich Bicinchoninic Acid Kit for Protein Determination. After AP immobilization and washing, the retained protein was evaluated by measuring the rection product absorbance signal at 570 nm and normalizing the response to the corresponding control. The BCA results qualitatively confirmed the EIS trend, showing higher AP retention on Clicked Biochar than on pristine SCG550, particularly at intermediate-to-high AP concentrations. The difference between the two materials was more clearly resolved by EIS, which is more sensitive to local interfacial changes affecting charge transfer.

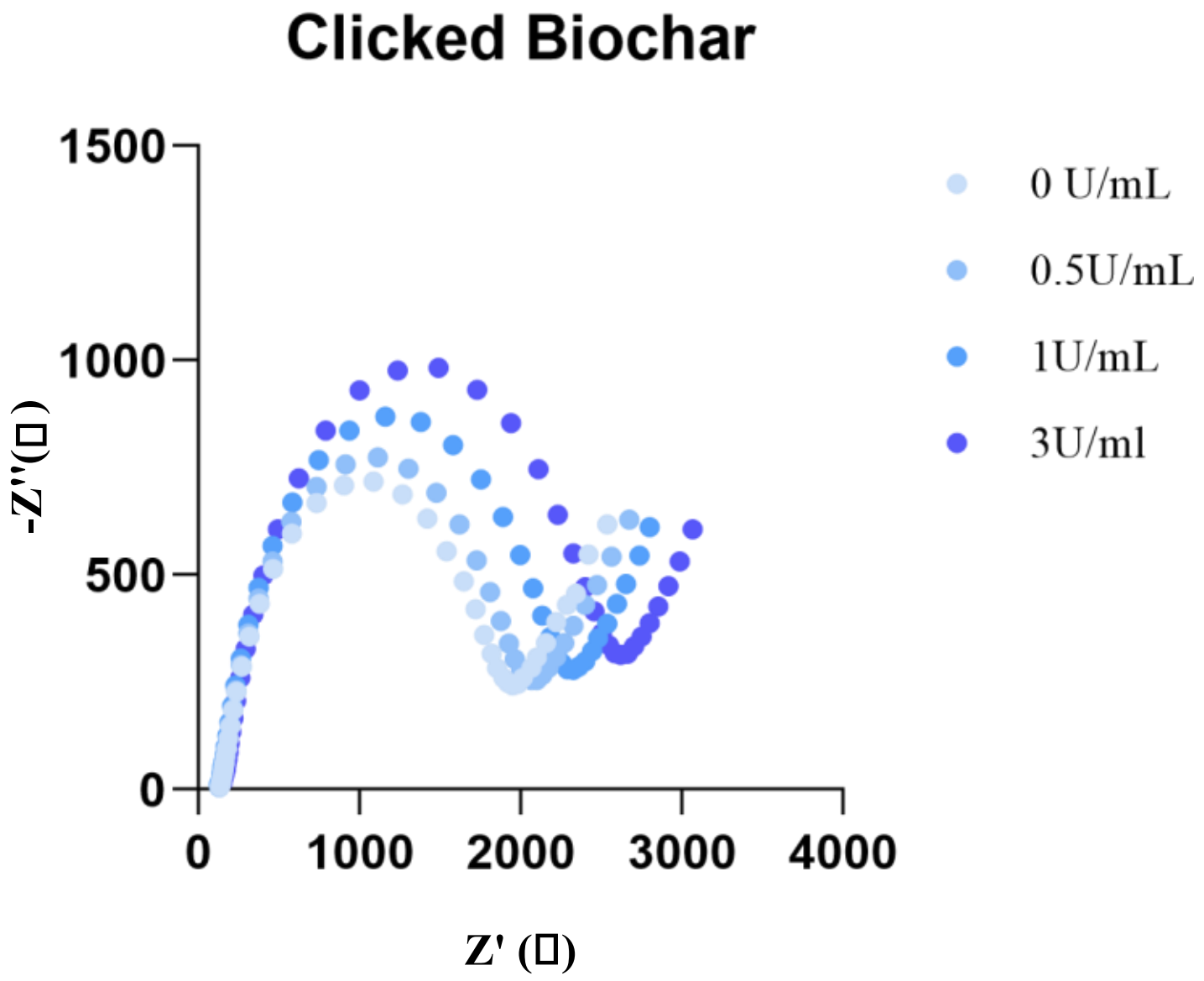

Figure S5.3. Representative Nyquist plots recorded for Clicked Biochar electrodes after AP immobilization at increasing AP concentrations using ferri/ferrocyanide as redox probe.

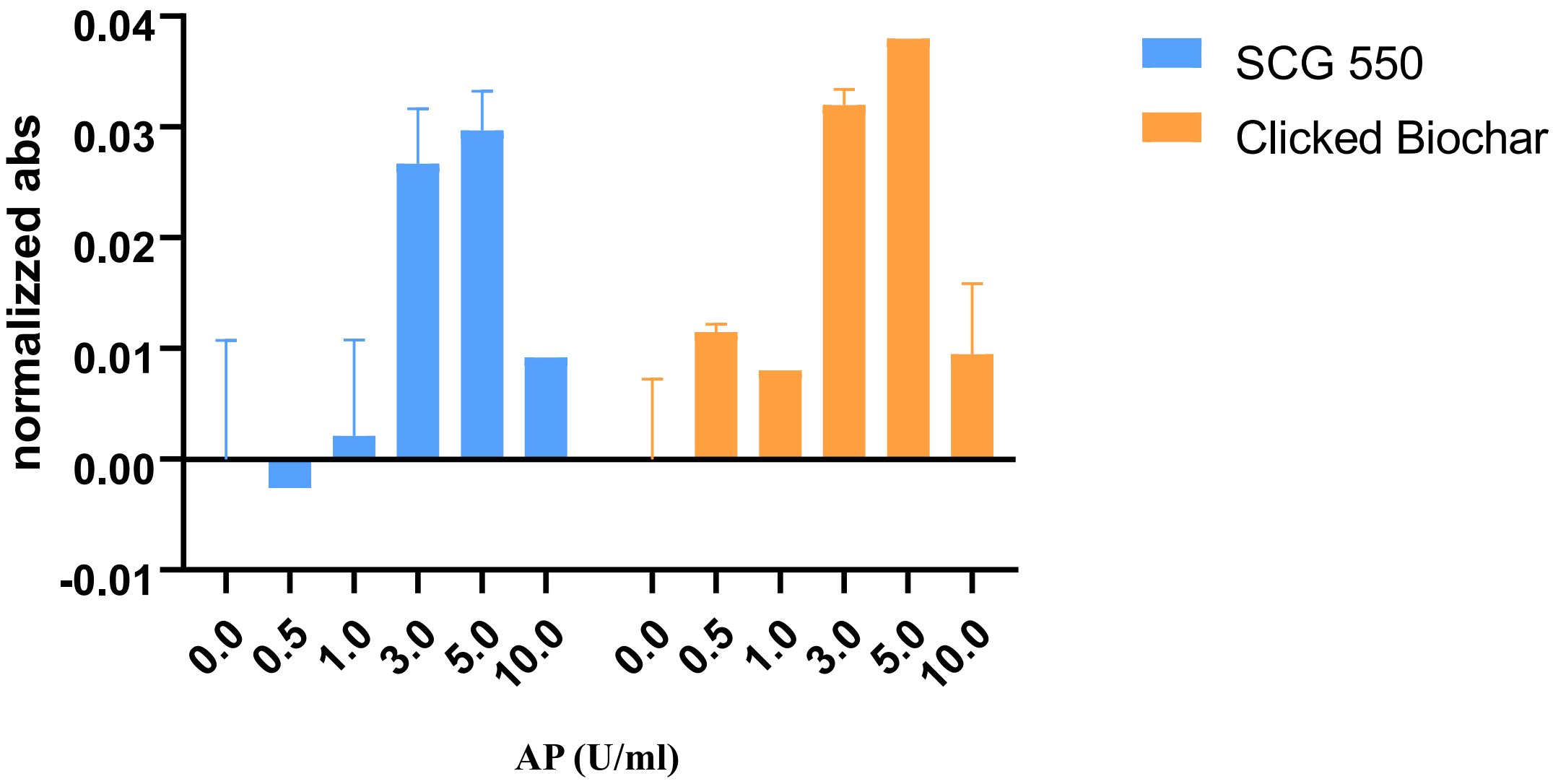

Figure S5.4. BCA assay performed after AP immobilization and washing on SCG550 and Clicked Biochar electrodes. Absorbance values were normalized to the corresponding control. Error bars represent standard deviation for n=3.

Fixed-substrate amperometric measurements

To evaluate whether the higher enzyme immobilization efficiency observed for material VI-AP also translates into preserved catalytic performance, amperometric experiments were performed using 5 mM 1-naphthyl phosphate (1-NPP) as substrate in 0.97 M DEA buffer (pH 9.8) with 0.08 M KCl and 1.0 mM $MgCl_2$. Measurements were carried out under chronoamperometric conditions at the enzyme redox potential identified via CV, applying a constant potential for 120 s (n = 3).

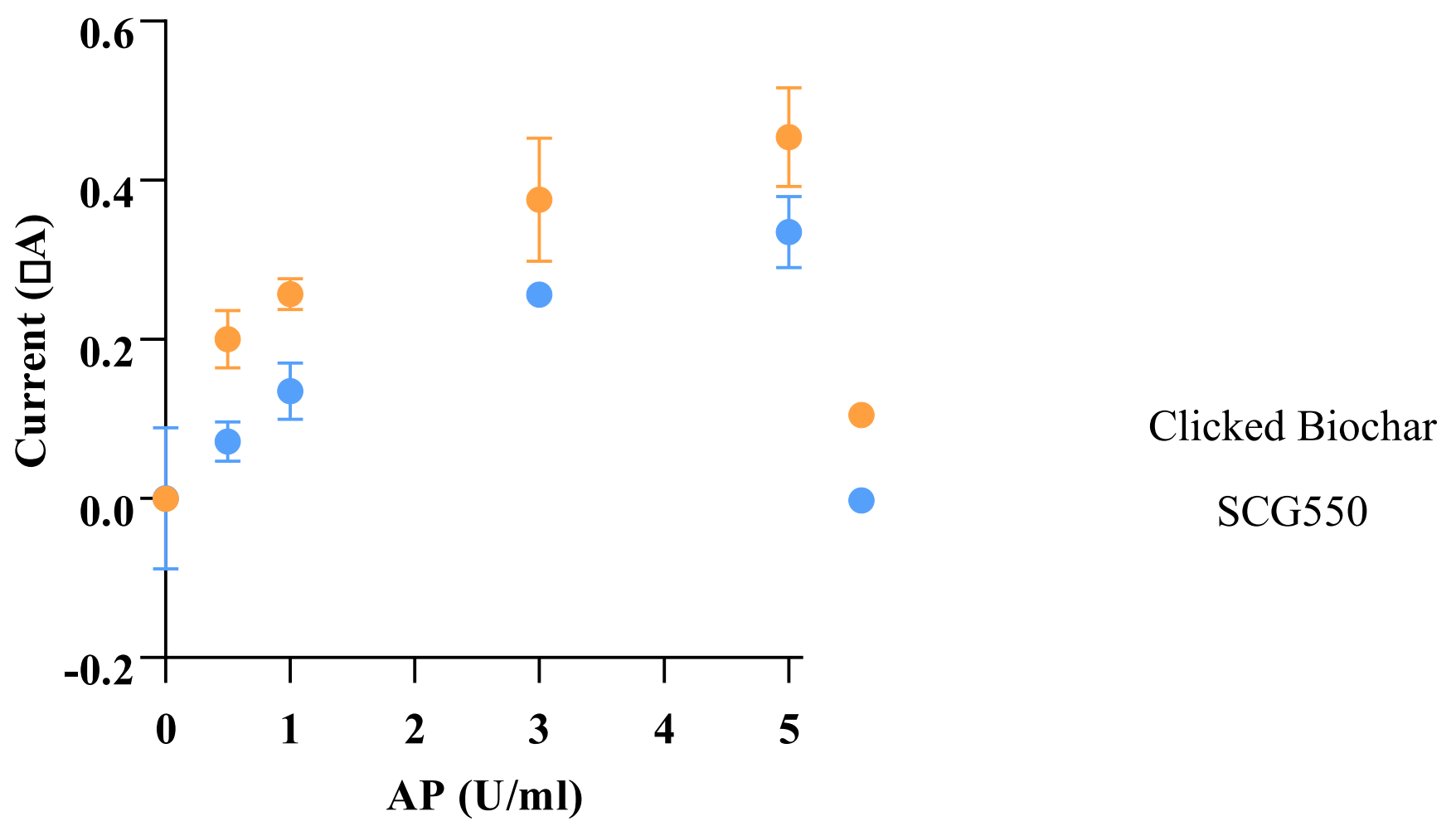

Figure S5.5 Fixed-substrate amperometric response as a function of enzyme loading. Chronoamperometric current values recorded after 120 s at increasing concentrations of immobilized alkaline phosphatase (AP) on SCG550 electrodes and material VI-AP electrodes. Error bars represent standard deviation.

The catalytic current increased with the AP concentration used during immobilization for both materials. However, Clicked Biochar consistently produced higher average current responses than pristine SCG550 at each AP concentration tested. This result indicates that the higher immobilization-associated response observed by EIS is accompanied by an increased functional enzymatic response. Therefore, the clicked spacer architecture does not merely promote protein retention at the surface, but also favors the formation of an enzyme layer that remains catalytically accessible.

These results support the proposed mechanism whereby the C10 molecular spacer alleviates steric hindrance and mass transport limitations, maintaining catalytic efficiency even at higher enzyme densities.
